# Ureteroscopy vs Shockwave Lithotripsy to Remove Kidney Stones in Children and Adolescents: A Nonrandomized Clinical Trial

**DOI:** 10.1001/jamanetworkopen.2025.25789

**Published:** 2025-08-07

**Authors:** Gregory E. Tasian, David I. Chu, Caleb P. Nelson, W. Robert DeFoor, Justin B. Ziemba, Jing Huang, Xianqun Luan, Michael Kurtz, Christina B. Ching, Pankaj Dangle, Anthony J. Schaeffer, Renea Sturm, Wayland Wu, Christopher Bayne, Nicolas Fernandez, Michael E. Chua, Romano DeMarco, Pamela Ellsworth, Brian Augelli, Jing Bi-Karchin, Rebecca D. McCune, Seth Vatsky, Susan Back, Zi Wang, Hunter Beck, Anna Kurth, Laura Kurth, Annabelle Pleskoff, Christopher B. Forrest, Jonathan S. Ellison, and the PKIDS Care Improvement Network, Kyle Rove, Scott Sparks, Eric Nelson, Bruce Schlomer, Aaron Krill, Ching Man Carmen Tong, Abby Taylor, Puneeta Ramachandra, Andrew Stec, Pasquale Casale, Douglas Coplen, Nicolette Janzen, Krystal Bagley, Michelle R. Denburg, Kimberley Dickinson, Rosemary Laberee, Matt Lorenzo, Antoine Selman-Fermin, Joana Dos Santos, Campbell Grant, Kate Kraft, Bhalaajee Meenakshi-Sundaram

**Affiliations:** 1 Division of Urology, Department of Surgery, The Children’s Hospital of Philadelphia, Philadelphia, Pennsylvania; 2 Department of Biostatistics, Epidemiology, and Informatics, Perelman School of Medicine at the University of Pennsylvania, Philadelphia; 3 Division of Urology, Department of Surgery, Ann & Robert H. Lurie Children’s Hospital of Chicago, Chicago, Illinois; 4 Department of Urology, Boston Children’s Hospital, Boston, Massachusetts; 5 Division of Urology, Cincinnati Children’s Hospital Medical Center, Cincinnati, Ohio; 6 Division of Urology, Department of Surgery, Perelman School of Medicine at the University of Pennsylvania, Philadelphia; 7 Department of Pediatric Urology, Kidney and Urinary Tract Center, Nationwide Children’s Hospital, Columbus, Ohio; 8 Department of Pediatric Urology, Riley Hospital for Children, Indianapolis, Indiana; 9 Division of Urology, Department of Surgery, University of Utah, Salt Lake City; 10 Department of Pediatric Urology, Mattel Children’s Hospital at UCLA, Los Angeles, California; 11 Department of Pediatric Urology, Bristol-Myers Squibb Children’s Hospital at Robert Wood Johnson University Hospital, New Brunswick, New Jersey; 12 Department of Pediatric Urology, Carilion Children’s Pediatric Urology, Roanoke, Virginia; 13 Division of Pediatric Urology, Seattle Children’s Hospital, Seattle, Washington; 14 Division of Urology, The Hospital for Sick Children, Toronto, Ontario, Canada; 15 Division of Pediatric Urology, Department of Urology, University of Florida, Gainesville; 16 Nemours Children’s Hospital, University of Central Florida College of Medicine, Orlando; 17 Department of Radiology, The Children’s Hospital of Philadelphia, Philadelphia, Pennsylvania; 18 Patient and Family Research Partners, The Children’s Hospital of Philadelphia, Philadelphia, Pennsylvania; 19 Department of Pediatrics, Applied Clinical Research Center, The Children’s Hospital of Philadelphia, Philadelphia, Pennsylvania; 20 Division of Pediatric Urology, Department of Urology, Medical College of Wisconsin, Milwaukee; 21 Department of Pediatric Urology, Children’s Hospital Colorado, Aurora; 22 Division of Urology, Children’s Hospital Los Angeles, Los Angeles, California; 23 Division of Urology, Children’s Hospital of Richmond, Virginia Commonwealth University, Richmond; 24 Department of Urology, University of Texas Southwestern, Dallas; 25 Department of Urology, Children’s National Hospital of Washington, Washington, DC; 26 Department of Urology, University of Alabama, Birmingham; 27 Department of Urology, Vanderbilt University Medical Center, Nashville, Tennessee; 28 Division of Urology, Nemours A. I. DuPont Hospital for Children, Wilmington, Delaware; 29 Division of Urology, Nemours Children’s Health, Jacksonville, Florida; 30 Division of Urology, Nemours Children’s Health, Delaware Valley, Wilmington, Delaware; 31 Division of Urology, St Louis Children’s Hospital, St Louis, Missouri; 32 Division of Urology, Texas Children’s Hospital, Houston; 33 Division of Urology, Children’s Hospital of Philadelphia, Philadelphia, Pennsylvania; 34 Applied Clinical Research Center, Children’s Hospital of Philadelphia, Philadelphia, Pennsylvania; 35 Department of Urology, University of Kentucky, Lexington; 36 Department of Urology, University of Michigan, Ann Arbor; 37 Department of Urology, University of Oklahoma Health Sciences, Oklahoma City

## Abstract

**Importance:**

Most children and adolescents with kidney and ureteral stones are treated with ureteroscopy, despite the uncertainty and equal weight of guideline recommendations for ureteroscopy or shockwave lithotripsy.

**Objective:**

To compare stone clearance and patient-reported outcomes among children and adolescents after ureteroscopy or shockwave lithotripsy.

**Design, Setting, and Participants:**

This nonrandomized clinical trial enrolled patients between March 16, 2020, and July 31, 2023, at 31 medical centers in the US and Canada. Patients aged 8 to 21 years with kidney stones, ureteral stones, or both were included. Follow-up was completed on October 15, 2023.

**Interventions:**

Ureteroscopy or shockwave lithotripsy.

**Main Outcomes and Measures:**

The primary outcome was stone clearance assessed by standardized ultrasonography 6 (±2) weeks after surgery. Using inverse probability weighting and random intercepts for site, stone clearance was evaluated per kidney or ureter using logistic regression and estimated stone clearance rates were generated for each procedure.

**Results:**

This study included 1142 patients (690 females [60.4%]), with a median age of 15.6 years (IQR, 12.6-17.3 years). In terms of race and ethnicity, 41 patients (3.6%) were Black, 130 (11.4%) were Hispanic, and 884 (77.4%) were White. A total of 124 urologists treated 1069 and 197 kidneys or ureters with ureteroscopy and shockwave lithotripsy (n = 953 and n = 189 patients), respectively, with a median stone size of 6.0 mm (IQR, 4.0-9.0 mm). Ureteral stents were placed at time of index surgery for 841 procedures for 767 patients (80.4%) receiving ureteroscopy and for 6 procedures for 5 patients (2.6%) receiving shockwave lithotripsy. Stone clearance occurred in 474 patients who underwent ureteroscopy (71.2% [95% CI, 63.8%-78.5%]) and in 105 patients who underwent shockwave lithotripsy (67.5% [95% CI, 61.0%-74.1%]), a difference that was not statistically significant (risk difference, 3.6% [95% CI, −6.2% to 13.5%]). Compared with shockwave lithotripsy, ureteroscopy resulted in greater pain interference (T-score difference, 5.0 [95% CI, 2.3-7.8]) and urinary symptoms (symptom score difference, 3.9 [95% CI, 1.2-6.7]) 1 week after surgery. Patients who had ureteroscopy missed more school (risk difference, 21.3% [95% CI, 9.7%-32.8%]) and caregivers missed more work (risk difference, 23.0% [95% CI, 11.0%-35.0%]) in the week after surgery.

**Conclusions and Relevance:**

In this study of 1142 children and adolescents with kidney and ureteral stones, there was no clinically meaningful difference in stone clearance with ureteroscopy vs shockwave lithotripsy. Shockwave lithotripsy was associated with better patient-reported outcomes. These findings raise questions about the preference for ureteroscopy in practice.

**Trial Registration:**

ClinicalTrials.gov Identifier: NCT04285658

## Key Points

**Question:**

What are the outcomes following ureteroscopy vs shockwave lithotripsy for kidney stone removal in children and adolescents?

**Findings:**

In this nonrandomized clinical trial including 1142 children and adolescents who underwent kidney stone surgery at 31 North American centers, stone clearance occurred in 71% of patients who underwent ureteroscopy compared with 68% of those who underwent shockwave lithotripsy, but this difference was not statistically significant. Ureteroscopy was associated with greater pain interference, urinary symptoms, and missed school 1 week after surgery.

**Meaning:**

There was no clinically meaningful difference in kidney stone clearance with ureteroscopy vs shockwave lithotripsy, but shockwave lithotripsy was associated with better lived experience.

## Teaser

This nonrandomized clinical trial compares kidney stone clearance and patient-reported experiences among children and adolescents following ureteroscopy or shockwave lithotripsy.

## Introduction

Kidney stones affect individuals across their lifespan and often necessitate surgical intervention.^1,2^ Ureteroscopy, an endoscopic surgery, and shockwave lithotripsy, a noninvasive procedure, are the most frequently used procedures to remove kidney and ureteral stones in children and adolescents.^3^ For children, both procedures are typically performed as outpatient surgeries under general anesthesia, but they differ in invasiveness and recovery. Ureteral stents, a driver of pain and urinary symptoms,^4^ may be placed with either modality but are used more commonly following ureteroscopy.^5^ After surgery, residual stone fragments often remain,^6,7^ which may move or grow, resulting in pain and additional surgery.

Clinical guidelines recommend ureteroscopy or shockwave lithotripsy for pediatric patients with ureteral stones and kidney stones smaller than 20 mm.^8,9^ This recommendation is graded C due to consistently low-quality evidence from observational studies and 3 randomized clinical trials. Most studies have demonstrated higher stone clearance for ureteroscopy than shockwave lithotripsy.^10^ None have considered patient experiences after surgery. Despite the uncertainty^10^ and equal weight of the recommendations for ureteroscopy and shockwave lithotripsy, 70% to 80% of youths in the US undergo ureteroscopy to remove kidney stones.^5,11^

The Pediatric Kidney Stone (PKIDS) Care Improvement Network was established to strengthen the evidence base supporting the treatment of children with nephrolithiasis. The PKIDS study arose from patients’ expressed need for improved understanding of the effectiveness of different stone surgeries and the impact of these procedures on their lives. We hypothesized that ureteroscopy would be associated with higher stone clearance^12^ and with lower physical, emotional, and social health compared with shockwave lithotripsy.

## Methods

### Study Design and Setting

The PKIDS study (NCT04285658) was an investigator-initiated, nonrandomized clinical trial embedded in the clinical care of children and adolescents undergoing kidney stone surgery between March 16, 2020, and July 31, 2023, at 31 medical centers in 22 US states and 1 Canadian province that participate in the PKIDS Network (eFigure 1 in Supplement 1). PKIDS was designated a National Patient-Centered Clinical Research Network (PCORnet) study due to the inclusion of medical centers that contribute data to 5 PCORnet clinical research networks. Institutional review board approval was obtained from the Children’s Hospital of Philadelphia, which served as the data coordinating center. Patients provided informed consent, with caregivers providing consent for children younger than 18 years who assented to participate and for patients who were unable to provide consent or assent. The trial followed the Transparent Reporting of Evaluations With Nonrandomized Designs (TREND) reporting guideline for nonrandomized clinical trials.

### Trial Population

We screened English- or Spanish-speaking patients aged 8 to 21 years undergoing ureteroscopy or shockwave lithotripsy for unilateral or bilateral kidney stones, and we enrolled eligible patients. The only exclusion criterion was a clinical situation in which delay to surgery would increase risk (eg, an obstructing stone and fever).

The lower age limit of 8 years was chosen to decrease heterogeneity of patient experiences introduced by using parent-proxy reports for younger children.^13^ We included patients up to age 21 years to align with pediatric care age ranges from the American Academy of Pediatrics.^14^ We ascertained characteristics of participants (preoperatively) and of surgeons and medical centers (before trial opening) that could influence choice of surgery, outcomes, or both.^15^ Participants self-reported race (Asian, Black, White, multiple races, or other race [not further specified]) and ethnicity (Hispanic or non-Hispanic). These data were collected to determine the representativeness of the trial population relative to patients treated with ureteroscopy and shockwave lithotripsy in the US. Three adolescents with nephrolithiasis and 4 parents of children with nephrolithiasis selected patient-reported outcomes (PROs), informed recruitment and retention efforts, and contextualized results.

### Outcomes

The primary outcome was stone clearance, defined as the absence of any stone larger than 4 mm in the operated kidney or ureter on ultrasonography 6 (±2) weeks after surgery. We collected all postoperative ultrasonographic images to ascertain stone clearance over longer time periods in sensitivity analyses. Ultrasonography is the preferred imaging for children with kidney stones, given its high sensitivity for kidney stones and lack of ionizing radiation.^16,17^ We chose 4 mm as the cut point because ultrasonography overestimates stone size by approximately 2 mm, a value commonly used to define stone clearance on computed tomography,^18,19,20^ and the risk of future surgery is greater for residual fragments larger than 2 mm.^21^ Ultrasonographic images were obtained in routine care and were interpreted by radiologists at each site (eAppendix 1 in Supplement 1). Results were reported using a standardized template (eAppendix 2 in Supplement 1). A pediatric radiologist (S.B.) blinded to the treatment groups interpreted a random 10% sample of preoperative and postoperative images. A second pediatric radiologist (S.V.) blinded to the treatment groups independently interpreted images with discordant local and central outcomes, with the final determination of stone clearance made by consensus.

The secondary outcomes were PROs at 1 week after surgery. Patient stakeholders selected pain intensity, pain interference (pain that disrupts daily functioning), anxiety, psychological stress, sleep disturbance, peer relationships, and urinary symptoms as the most meaningful postoperative experiences. Questionnaires were administered preoperatively, at the primary PRO end point of 1 week postoperatively, and at 3, 6, and 12 weeks postoperatively. All PROs except urinary symptoms were measured using English and Spanish versions of Patient-Reported Outcomes Measurement Information System (PROMIS) questionnaires administered through REDCap using computer adaptive testing.^22,23^ These questionnaires are validated for children aged 8 to 17 years and are recommended for studies with small numbers of young adults.^24^ We calibrated scores to the PROMIS T scale.^25,26^ Urinary symptoms were measured with the Dysfunctional Voiding Symptoms Score (DVSS),^27^ which is validated in children with bowel and bladder dysfunction, and the Questionnaire for Urinary Issues—Kidney Stone Surgery (QUIKSS) (eAppendix 3 in Supplement 1). The QUIKSS was developed by PKIDS investigators and patient partners for the PKIDS study using PROMIS methodology for item construction^28^ to capture urinary symptoms experienced after stone surgery. Additional outcomes included missed school and work up to 6 weeks postoperatively and emergency department visits, hospitalizations, and surgical reintervention within 3 months of index surgery. Health care encounters were obtained through medical record review and self-report, which were independent sources of outcomes to capture care received at different health systems from where the index surgery occurred.

### Comparators

The comparators were ureteroscopy and shockwave lithotripsy. Treatment type was a clinical decision made by the urologist and patient or caregiver (or both) and was performed per the urologist’s discretion. We examined heterogeneity of treatment effect (HTE) for the following prespecified categories: stone clearance by stone size (<7, 7-10, >10-15, or >15 mm) and stone location (ureter or ureteropelvic junction, non–lower pole kidney, or lower pole kidney). Stone size was the linear measurement of the largest stone in the operated kidney or ureter and was classified as a categorical variable to be more interpretable to clinicians. We examined HTE for PROs by sex (female or male) and age (8-11, 12-14, 15-18, or 19-21 years).

### Statistical Analysis

The enrollment target of 1290 kidneys or ureters with an expected 4:1 ureteroscopy to shockwave lithotripsy ratio had 80% power with α = .05 to detect a 15% difference^29^ in stone clearance between treatments, a difference that patients and caregivers expressed would change treatment decisions. The study was not powered for HTE analyses. Intention-to-treat analysis was performed based on the index surgery selected. The primary analysis used multiple imputation for the outcome (eTable 1 in Supplement 1).

We used propensity score methods to reduce confounding. The propensities for surgery type were estimated using a multivariable logistic regression model that included patient (eg, stone size and location and kidney anomaly), surgeon (eg, strength of preference for ureteroscopy) (eTable 2 in Supplement 1), and institutional (eg, patient volume) (eTable 3 in Supplement 1) characteristics. The propensity score model was constructed using covariates without incorporating the outcome. The weights remained consistent across all imputed datasets.

All analyses used inverse probability weighting and random intercepts for site. Stone clearance was evaluated per kidney or ureter using logistic regression analysis, and estimated stone clearance rates were generated for each procedure. We used linear regression analysis to evaluate PROs at the participant level at 1 week and other time points after surgery, adjusting for baseline scores. We assessed minimal important change by quantifying the differences in PROMIS T scores between treatments^30^ and the proportion of participants whose urinary symptom scores at 1 week exceeded 50% of the SD of baseline scores.^31^ To explore recovery trajectory, separate models were fit for 3, 6, and 12 weeks after surgery. HTE was analyzed using stratified analyses.

We performed the following sensitivity analyses: (1) complete data, (2) different assumptions for missing data, (3) stone clearance considering ultrasonographic images obtained up to 16 weeks and closest to 6 weeks after surgery, (4) weighting by misclassification of stone clearance based on central imaging review, (5) exclusion of patients older than 18 years, and (6) exclusion of patients with kidney anomalies reported in Table 1. We also compared PROs excluding patients who had a stent placed at time of index surgery.

**Table 1.** Characteristics of Patients Undergoing Ureteroscopy or Shockwave Lithotripsy^a^

Characteristic	All patients (N = 1142)	Treatment group	
		Ureteroscopy (n = 953)	Shockwave lithotripsy (n = 189)
Age, y			
Median (IQR)	15.6 (12.6-17.3)	15.6 (12.6-17.2)	15.6 (12.5-17.6)
Group			
8-11	235 (20.6)	191 (20.0)	44 (23.3)
12-15	397 (34.8)	341 (35.8)	56 (29.6)
16-18	402 (35.2)	338 (35.5)	64 (33.9)
19-21	108 (9.5)	83 (8.7)	25 (13.2)
Sex			
Female	690 (60.4)	590 (61.9)	100 (52.9)
Male	452 (39.6)	363 (38.1)	89 (47.1)
Race			
Asian	15 (1.3)	14 (1.5)	1 (0.5)
Black	41 (3.6)	38 (4.0)	3 (1.6)
White	884 (77.4)	738 (77.4)	146 (77.2)
Other race^b^	39 (3.4)	29 (3.0)	10 (5.3)
Multiple races	53 (4.6)	46 (4.8)	7 (3.7)
Missing or unknown	110 (9.6)	88 (9.2)	22 (11.6)
Ethnicity			
Hispanic	130 (11.4)	110 (11.5)	20 (10.6)
Non-Hispanic	902 (79.0)	750 (78.7)	152 (80.4)
Missing or unknown	110 (9.6)	93 (9.8)	17 (9.0)
BMI, median (IQR)	22.2 (18.6-27.4)	22.2 (18.7-27.7)	21.6 (17.5-26.5)
No. of stones in treated kidney, median (IQR)	1 (1-2)	1 (1-2)	1 (1-2)
Cumulative stone size in treated kidney, median (IQR), mm	7.0 (5.0-11.0)	7.0 (4.0-11.0)	8.8 (6.0-11.2)
Largest stone size in treated kidney, median (IQR), mm	6.0 (4.0-9.0)	6.0 (4.0-9.0)	7.6 (6.0-9.9)
Largest stone size group, mm			
No stone detected	57 (5.0)	55 (5.8)	2 (1.1)
<7	516 (45.2)	451 (47.3)	65 (34.4)
7 to <10	334 (29.2)	246 (25.8)	88 (46.6)
10 to <15	124 (10.9)	100 (10.5)	24 (12.7)
≥15	51 (4.5)	46 (4.8)	5 (2.6)
Missing size	45 (3.9)	41 (4.3)	4 (2.1)
Missing or unknown	15 (1.3)	14 (1.5)	1 (0.5)
Stone location			
No stone	57 (5.0)	55 (5.8)	2 (1.1)
Lower pole kidney	236 (20.7)	170 (17.8)	66 (34.9)
Non–lower pole kidney	351 (30.7)	257 (27.0)	94 (49.7)
Ureter (includes ureteropelvic junction)	483 (42.3)	457 (48.0)	26 (13.8)
Missing or unknown	15 (1.3)	14 (1.5)	1 (0.5)
History of prior stone surgery	198 (17.3)	162 (17.0)	36 (19.0)
Primary indication for stone surgery			
Elective	164 (14.4)	97 (10.2)	67 (35.4)
Pain	706 (61.8)	635 (66.6)	71 (37.6)
Urinary tract infection	226 (19.8)	178 (18.7)	48 (25.4)
Other	46 (4.0)	43 (4.5)	3 (1.6)
Presurgical drainage			
None	836 (73.2)	658 (69.0)	178 (94.2)
Stent	280 (24.5)	271 (28.4)	9 (4.8)
Nephrostomy tube	10 (0.9)	10 (1.0)	0
Other	5 (0.4)	3 (0.3)	2 (1.1)
Missing or unknown	11 (1.0)	11 (1.2)	0
Presentation to emergency department before index surgery	718 (62.9)	643 (67.5)	75 (39.7)
Structural kidney abnormality in treated side			
Any abnormality	122 (10.7)	112 (11.8)	10 (5.3)
Horseshoe kidney	3 (0.3)	3 (0.3)	0
Malrotation	7 (0.6)	7 (0.7)	0
Pelvic kidney	5 (0.4)	5 (0.5)	0
Other	103 (9.0)	93 (9.8)	10 (5.3)
Comorbid condition			
Neurogenic bladder	90 (7.9)	80 (8.4)	10 (5.3)
Ventilator dependent	12 (1.1)	12 (1.3)	0
Neuromuscular disorder	149 (13.0)	118 (12.4)	31 (16.4)
Hematologic disorder	50 (4.4)	42 (4.4)	8 (4.2)
Oxygen support	26 (2.3)	25 (2.6)	1 (0.5)
Cardiac risk factors	36 (3.2)	32 (3.4)	4 (2.1)
Structural central nervous system abnormality	32 (2.8)	26 (2.7)	6 (3.2)
Developmental delay	138 (12.1)	115 (12.1)	23 (12.2)
Epilepsy	71 (6.2)	66 (6.9)	5 (2.6)
Food insecurity			
Often true	11 (1.0)	9 (0.9)	2 (1.1)
Sometimes true	69 (6.0)	59 (6.2)	10 (5.3)
Never true	917 (80.3)	764 (80.2)	153 (81.0)
Missing or unknown	145 (12.7)	121 (12.7)	24 (12.7)

Abbreviation: BMI, body mass index (calculated as weight in kilograms divided by height in meters squared).

^a^ Unless specified otherwise, values are reported as No. (%) of participants.

^b^ Categories were not further specified.

### Assessment of Generalizability

We compared demographics of study participants to patients aged 8 to 21 years who had ureteroscopy or shockwave lithotripsy during the same period at 58 institutions that contribute data to PCORnet. We compared demographics and affected body regions, defined by the Pediatric Medical Complexity algorithm,^32,33^ of patients aged 8 to 21 years who had ureteroscopy or shockwave lithotripsy during the same period at PKIDS sites and non-PKIDS sites in PCORnet.

A 2-sided α = .05 was used for statistical tests. Analyses were performed using R, version 4.4 (R Project for Statistical Computing). Details are provided in the trial protocol and statistical analysis plan in Supplement 2.^34^

## Results

This study included 1142 patients (690 females [60.4%] and 452 males [39.6%]), with a median age of 15.6 years (IQR, 12.6-17.3 years) (Table 1). In terms of race and ethnicity, 15 patients (1.3%) identified as Asian, 41 (3.6%) as Black, 884 (77.4%) as White, 39 (3.4%) as other race, and 53 (4.6%) as multiple races; 130 patients (11.4%) identified as Hispanic and 902 (79.0%) as non-Hispanic. Race and ethnicity were missing or unknown for 110 patients (9.6%). A total of 124 urologists treated 1069 and 197 kidneys or ureters with ureteroscopy and shockwave lithotripsy (n = 953 and 189 patients), respectively (Figure). The median stone size was 6.0 mm (IQR, 4.0-9.0 mm). Of the 1142 patients, 116 (10.2%) received bilateral simultaneous or staged ureteroscopy and 7 (0.6%) received bilateral staged shockwave lithotripsy. A total of 83 of 1069 (7.8%) and 3 of 197 (1.5%) kidneys had ipsilateral repeat ureteroscopy or shockwave lithotripsy after the index surgery, respectively. The top indication for both treatments was pain (Table 1).

**Figure.**
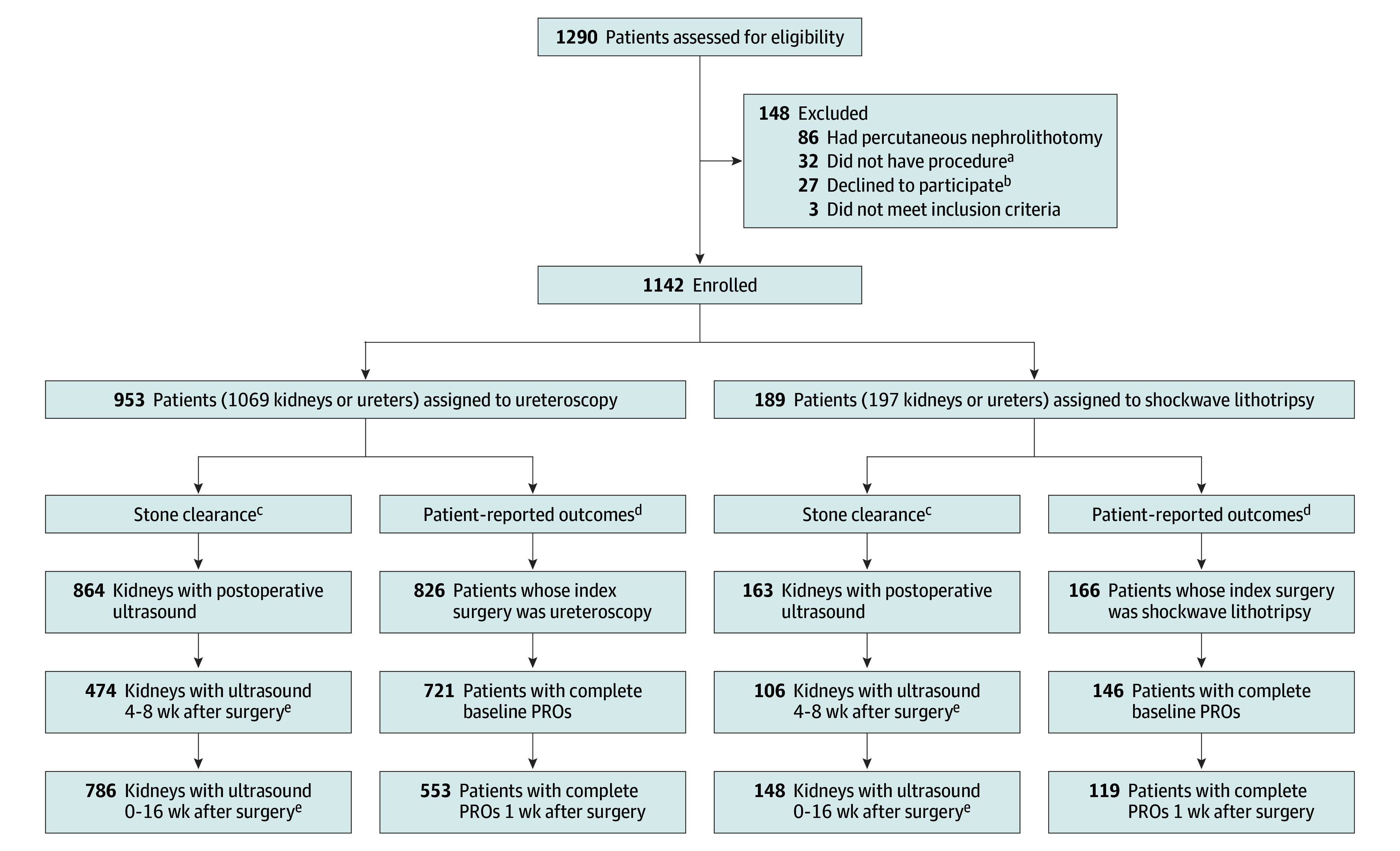
Flow Diagram of Patients Who Underwent Ureteroscopy or Shockwave Lithotripsy for Kidney Stone Removal ^a^Participants did not receive ureteroscopy or shockwave lithotripsy. ^b^Participants who withdrew but allowed data collection remained in the analysis cohort. ^c^Stone clearance evaluated at the kidney level. ^d^Treatment group allocation and patient-reported outcome (PRO) evaluation are at the patient level. ^e^Ultrasonography studies either reported no stone present or had measured stone size.

Surgeon and hospital characteristics are reported in eTables 4 and 5 in Supplement 1. After propensity score weighting, 86 of 88 patient, surgeon, and hospital characteristics had standardized mean differences of less than 0.10 between groups, and all had standardized mean differences of less than 0.25 (eFigure 2 and eTable 4 in Supplement 1).

Of the 109 patients who had a stent placed before ureteroscopy, 78 (71.5%) had stones in the kidney and 31 (28.4%) had stones in the ureter. Ureteral stents were placed at time of index surgery for 841 procedures for 767 of 953 patients (80.4%) receiving ureteroscopy and for 6 procedures for 5 of 189 patients (2.6%) receiving shockwave lithotripsy. The median stent duration was 5 days (IQR, 4-14 days) for ureteroscopy and 21 days (IQR, 10-23 days) for shockwave lithotripsy. For the ureteroscopy group, 527 of 841 participants (62.7%) had a stent on a string removed at home or in the clinic, and 285 of 841 (33.9%) had a stent removed by cystoscopy under general anesthesia. Six participants (100%) receiving shockwave lithotripsy had stents removed under general anesthesia. Overall, 285 of 953 (29.9%) patients who underwent ureteroscopy and 6 of 189 (3.2%) patients who underwent shockwave lithotripsy had secondary procedures under general anesthesia after the index surgery.

In the ureteroscopy and shockwave lithotripsy groups, patients underwent ultrasonography examinations for 474 of 1069 kidneys (44.3%) and 106 of 189 kidneys (56.1%), respectively, 4 to 8 weeks after surgery; in addition, patients had undergone ultrasonography examinations for 786 of 1069 kidneys (73.5%) and 148 of 197 kidneys (75.1%), respectively, by 16 weeks after surgery. The median time after surgery was 6.3 weeks (IQR, 4.7-9.1 weeks). Overall, 547 of 1142 patients (47.9%) and 852 of 1142 patients (74.6%) had undergone ultrasonography 4 to 8 weeks and by 16 weeks after surgery, respectively.

### Primary Outcome: Stone Clearance

Stone clearance occurred in 474 patients who underwent ureteroscopy (71.2% [95% CI, 63.8%-78.5%]) and in 105 patients who underwent shockwave lithotripsy (67.5% [95% CI, 61%-74.1%]), a difference that was not statistically significant (risk difference, 3.6% [95% CI, −6.2% to 13.5%]). On central review of 258 participants, stone clearance for 15 patients (5.8%) differed from local interpretations. The sensitivity (97.0%) and specificity (79.0%) of local ultrasonography interpretation were similar between treatments.

We did not consider stones larger than 15 mm in HTE analyses, as only 46 (4.8%) and 5 (2.6%) patients underwent ureteroscopy or shockwave lithotripsy, respectively, for stones larger than 15 mm. There was no difference in clearance for stones 15 mm or smaller or by stone location (Table 2). When the largest stone size was analyzed as a continuous variable, clearance for shockwave lithotripsy decreased for stones larger than 10 mm, while clearance for ureteroscopy remained stable (eFigure 3 in Supplement 1).

**Table 2.** Kidney Stone Clearance for Ureteroscopy and Shockwave Lithotripsy at 4 to 8 Weeks After Surgery, Overall and by Stone Size and Stone Location^a^

Stone clearance	Ureteroscopy, No. (% [95% CI])	Shockwave lithotripsy, No. (% [95% CI])	Risk difference (95% CI)
Overall	474 (71.2 [63.8-78.5])	105 (67.5 [61.0-74.1])	3.6 (−6.2 to 13.5)
Stone size, mm			
<7	215 (73.8 [54.8-92.9])	32 (79.6 [69.4-89.7])	−5.7 (−25.9 to 14.4)
7-10	137 (68.7 [55.8-81.6])	53 (64.1 [50.1-78.2])	4.6 (−14.6 to 23.8)
>10-15	52 (67.3 [44.3-90.2])	12 (51.7 [32.2-71.1])	15.6 (−12.6 to 43.8)
Stone location			
Lower pole kidney	99 (68.9 [55.4- to 82.5])	31 (67.7 [51.4-84.1])	1.2 (−18.2 to 20.5)
Non–lower pole kidney	120 (65.8 [53.4-78.3])	56 (60.9 [50.6-71.2])	4.9 (−12.0 to 21.8)
Ureter (includes ureteropelvic junction)	223 (84.9 [70.1-99.7])	15 (93.5 [82.4-104.5])	−8.6 (−26.3 to 9.2)

^a^ Results are weighted estimates from the logistic regression model and reflect analyses of imputed data. Variables used to impute missing data included all baseline covariates, treatment variable, and observed outcomes that were used in the analysis. Twenty imputed datasets were created and analyzed separately, and the results were combined using Rubin rules to produce valid statistical inferences. The numbers of patients in each treatment group category indicate the actual number of patients with stone clearance rather than the weighted number of patients.

### Secondary Outcomes

Adjusting for preoperative symptoms, ureteroscopy was associated with higher pain intensity, pain interference, and QUIKSS urinary symptoms at 1 week postoperatively compared with shockwave lithotripsy (Table 3 and eFigure 4 in Supplement 1). There were no statistically significant differences in DVSS values between groups. A total of 238 of 553 patients in the ureteroscopy group (43.0% [95% CI, 36.5%-60.9%]) had QUIKSS urinary symptom scores at 1 week that exceeded 50% of the SD of the baseline scores compared with 33 of 120 in the shockwave lithotripsy group (27.5% [95% CI, 19.5%-35.5%]), a difference of 21.2% (95% CI, 6.6%-35.8%) (eTable 5 in Supplement 1). There were no differences in PROs between treatments after week 1. Patients who received ureteroscopy missed more school (risk difference, 21.3% [95% CI, 9.7%-32.8%]) and caregivers missed more work (risk difference, 23.0% [95% CI, 11.0%-35.0%]) in the week after surgery (Table 4). There were no differences in emergency department visits, hospitalizations, or unanticipated surgery between treatments (eTable 6 in Supplement 1). Ureteroscopy among patients aged 19 to 21 years was associated with worse urinary symptoms at week 1 compared with younger patients undergoing ureteroscopy, an association that did not exist for shockwave lithotripsy (eFigure 5 in Supplement 1). There were no sex differences in PROs between treatments (eFigure 6 in Supplement 1).

**Table 3.** PROs at Baseline and at 1 Week After Surgery^a^

PRO instrument	Weighted mean score (95% CI)				Procedure effect of ureteroscopy vs shockwave lithotripsy, controlling for baseline, β (95% CI)
	Baseline		Postoperative		
	Ureteroscopy (n = 721)	Shockwave lithotripsy (n = 146)	Ureteroscopy (n = 553)	Shockwave lithotripsy (n = 119)	
PROMIS					
Pain intensity	45.4 (43.3-47.6)	41.1 (39.6-42.7)	49.3 (47.1-51.5)	42.8 (41.3-44.2)	3.3 (0.9-5.6)
Pain interference	51.7 (48.9-54.5)	45.4 (43.5-47.3)	58.7 (56-61.5)	48.7 (46.7-50.7)	5.0 (2.3-7.8)
Anxiety	50.9 (48-53.7)	47 (45.1-48.8)	52 (48.6-55.2)	46.1 (44.3-47.9)	1.7 (−0.6 to 4)
Peer relationships	48.3 (46.6-50.1)	48.5 (47-49.9)	46.8 (44.6-48.9)	47.8 (45.9-49.6)	−1.3 (−3.5 to 0.9)
Sleep disturbances	56.6 (54.2-59.1)	52.8 (51.1-54.4)	56.5 (53.7-59.3)	52.4 (50.7-54.1)	1.0 (−0.9 to 3)
Stress experiences	54.6 (52.1-57.1)	51.2 (49.5-52.9)	53.5 (51.1-55.8)	49.3 (47.4-51.2)	0.6 (−1.6 to 2.8)
DVSS value (range, 0-30; urinary symptoms)	7 (6.2-7.8)	6.4 (5.7-7)	7.2 (6.3-8.2)	5.5 (4.9-6.2)	0.8 (−0.4 to 1.95)
QUIKSS score (range, 0-56; urinary symptoms)^b^	13.8 (11.5-16.2)	8.4 (7-9.8)	19.4 (16.9-22)	19.4 (16.8-22)	3.9 (1.2-6.7)

Abbreviations: DVSS, Dysfunctional Voiding Symptom Score; PRO, patient-reported outcome; PROMIS, Patient-Reported Outcomes Measurement Information System; QUIKSS, Questionnaire for Urinary Issues–Kidney Stone Surgery.

^a^ Results reflect analyses of imputed data. Variables used to impute missing data included all baseline covariates, treatment variable, and observed outcomes that were used in the analysis. Twenty imputed datasets were created and analyzed separately, and the results were combined using Rubin rules to produce valid statistical inferences.

^b^ The QUIKSS is composed of 16 items that used a frequency response scale and a 7-day recall period. Each item was scored from never (0) to always (4). The total score was the sum of the items.

**Table 4.** Participants Who Missed School and Caregivers Who Missed Work in the Last Week at 1, 3, and 6 Weeks After Ureteroscopy or Shockwave Lithotripsy

Postoperative follow-up	Ureteroscopy, No. (% [95% CI])	Shockwave lithotripsy, No. (% [95% CI])	Difference (95% CI)
Participant missed school in the last week			
Week 1	407 (85.5 [79.0-92.1])	63 (64.3 [54.8-73.8])	21.3 (9.7-32.8)
Week 3	115 (23.3 [12.9-33.7])	12 (12.1 [5.7-18.6])	11.2 (−1.0 to 23.4)
Week 6	68 (11.9 [5.3-18.5])	15 (14.0 [7.4-20.6])	−2.1 (−11.4 to 7.2)
Caregiver missed work in the last week			
Week 1	338 (58.1 [45.8-70.3])	44 (37.3 [28.6-46])	23.0 (11.0-35.0)
Week 3	72 (14.0 [5.3-22.7])	5 (4.4 [0.6-8.2])	13.4 (1.0-25.8)
Week 6	41 (3.7 [0.9-6.6])	7 (6.1 [1.7-10.6])	−1.3 (−10.9 to 8.4)

### Sensitivity Analyses

Results were similar to primary analyses in complete data analyses (eTables 7 and 8 in Supplement 1) when the primary outcome window was extended to 16 weeks after surgery (eTable 9 in Supplement 1), when missing stone clearance outcomes were assumed to be all not cleared or all cleared (eTable 10 in Supplement 1), and when patients aged 19 to 21 years (eTable 11 in Supplement 1) and patients with kidney anomalies (eTable 12 in Supplement 1) were excluded. When models were weighted using misclassification estimates from central ultrasonography review, stone clearance was higher for ureteroscopy than shockwave lithotripsy for stones larger than 10 to 15 mm (risk difference, 35.5% [95% CI, 0.5%-70.5%]) (eTable 13 in Supplement 1). When missing outcomes were replaced with interval values ranging from 25% lower to 25% higher than complete data estimates, results only changed at the extremes or with assumptions of differential clearance (eFigures 7 and 8 and eTables 14 and 15 in Supplement 1). There was no association between ureteroscopy and greater pain and urinary symptoms among patients who did not have stents (eTable 16 in Supplement 1).

### Assessment of Generalizability

The sex distribution of participants was similar to PCORnet populations. Study participants were younger, and there was a higher proportion of White patients than in PCORnet populations (eFigures 9-12 in Supplement 1). Except for patients at PKIDS sites who had more musculoskeletal and neurologic conditions, affected body systems of patients treated at PKIDS and non-PKIDS sites in PCORnet were similar (eTable 17 in Supplement 1).

## Discussion

This nonrandomized clinical trial embedded in clinical care comparing ureteroscopy and shockwave lithotripsy in children and adolescents generated 4 clinically meaningful findings. First, we did not detect clinically meaningful differences in stone clearance between ureteroscopy and shockwave lithotripsy, despite more patients in the ureteroscopy group having repeat surgery to clear stones. Second, compared with ureteroscopy, shockwave lithotripsy was associated with less pain and lower urinary symptoms during the first week after surgery. Third, patients who received shockwave lithotripsy missed less school and their caregivers missed less work than patients who received ureteroscopy. Fourth, compared with shockwave lithotripsy, 10 times as many patients who underwent ureteroscopy (285 of 953 [29.9%] vs 6 of 189 [3.2%]) required a second procedure under general anesthesia to remove a ureteral stent. This evidence generated from contemporary clinical care calls into question current clinical practice, wherein most children and adolescents with kidney stones receive ureteroscopy.^5^

Stone clearance for ureteroscopy in this study (71.2%) was lower than the 87% reported in a systematic review of primarily retrospective studies of pediatric patients using different imaging modalities.^35^ It is similar to the 68% clearance determined by ultrasonography in a multisite study of adults.^36^ We did not detect statistically significant differences in stone clearance by stone size or location; however, clinically meaningful differences may exist, as this study was not powered to detect HTE. There was some evidence that ureteroscopy was associated with higher clearance of stones larger than 10 mm, which is consistent with reports of lower clearance for stones larger than 10 mm treated with shockwave lithotripsy.^37^ The potential for lower stone clearance with shockwave lithotripsy compared with ureteroscopy should be discussed for patients with stones larger than 10 mm.

Although minimal important change values have not been established for pediatric surgery, differences in pain intensity and pain interference 1 week after ureteroscopy exceeded 3 PROMIS T-score points, a commonly used threshold for minimal important change.^30^ Nearly twice as many patients undergoing ureteroscopy had QUIKSS urinary symptoms scores at 1 week that exceeded 50% of the SD of baseline scores, another definition of minimum important change.^31^ Differences in urinary symptoms were not detected in DVSS values, which were less sensitive after kidney stone surgery compared with scores on the QUIKSS, which has not been validated. The greater pain and urinary symptoms with ureteroscopy were likely driven by ureteral stents,^38^ as the significance of the differences between groups was lost when excluding patients who received a stent, although the power to detect differences was lower due to the smaller sample size. The greater symptoms were particularly pronounced in adolescents who may preferentially benefit from shockwave lithotripsy or from the elimination of stents when feasible if ureteroscopy is selected.

There are several aspects of this study for clinicians to consider when determining how these results should influence their practice. By engaging patients and caregivers in the study design, these results reflect outcomes that are meaningful to patients. The impact of ureteroscopy and shockwave lithotripsy on physical, emotional, and social health and on return to school and work demonstrated in this study was previously unknown. Second, these results reflect the spectrum of children and adolescents with kidney stones treated at medical centers representative of pediatric kidney stone care. These findings can be used to improve selection of shockwave lithotripsy or ureteroscopy and to counsel patients on expected postoperative experiences.

### Limitations

This study has limitations. One limitation is the lack of participant randomization. The PKIDS study was designed as a nonrandomized clinical trial because of the lack of clinical equipoise between ureteroscopy and shockwave lithotripsy, which makes randomization difficult, if not impossible. Although we balanced many patient-, surgeon-, and health system–level confounders, the possibilities of unmeasured confounding and bias remain. Second, 547 of 1142 patients (47.9%) underwent ultrasonography within the 4-to-8-week postoperative window, and 852 of 1142 (74.6%) underwent ultrasonography by 16 weeks after surgery. Postoperative imaging was affected by the COVID-19 pandemic, which began when the study opened. Although other studies have shown that stone clearance increases with greater time from index surgery,^39^ sensitivity analyses considering ultrasonographic images obtained by 16 weeks and different missingness assumptions were similar to the primary analysis except for extreme scenarios. Third, ultrasonography has limited ability to differentiate a cluster of small fragments after surgery from a single larger residual stone. Because residual stones 2 mm or larger are more likely to lead to future surgery and stone growth, misclassification of the size of residual fragments would be clinically meaningful.^40^ Fourth, we did not consider the effect of postoperative analgesics on PROs. Finally, we did not consider intraoperative technical aspects that may impact procedural effectiveness.

## Conclusions

Among patients aged 8 to 21 years with kidney and ureteral stones in this nonrandomized clinical trial, shockwave lithotripsy resulted in no clinically meaningful differences in stone clearance and better lived experiences during the first week after surgery compared with ureteroscopy. However, shockwave lithotripsy was associated with better PROs. These findings raise questions about the preference for ureteroscopy in practice.

## Notes

Supplement 1. **eAppendix 1.** PKIDS Standardized Ultrasonography Protocol **eAppendix 2.** PKIDS Ultrasonography Report **eAppendix 3.** Questionnaire for Urinary Issues—Kidney Stone Surgery (QUIKSS) **eFigure 1.** Pediatric KIDney Stone Care Improvement Network **eFigure 2.** Balance of Patient, Surgeon, and Health System Characteristics Between URS and SWL Treatment Groups Before and After Propensity Score Weighting **eFigure 3.** Difference in Stone Clearance Between URS and SWL With Preoperative Largest Stone Size Estimated as a Continuous Variable **eFigure 4.** Impact of URS and SWL on Recovery of Physical, Emotional, and Social Health **eFigure 5.** Impact of URS and SWL on Patient-Reported Outcomes of Physical, Emotional, and Social Health After Surgery, by Age Group at Baseline (BL) and to 3 Months After Surgery **eFigure 6.** Impact of URS and SWL on Patient-Reported Outcomes of Physical, Emotional, and Social Health After Surgery, by Sex Group at Baseline and to 3 Months After Surgery **eFigure 7.** Results of Multiple Imputation Analyses Replacing Missing Stone Clearance With a Range of Values That Are Nondifferential Across URS and SWL **eFigure 8.** Results of Multiple Imputation Analyses Replacing Missing Stone Clearance With a Range of Values That Are Differential Across URS and SWL **eFigure 9.** Distribution of Age of PKIDS Trial Participants and at PCORnet Sites That Participate in PKIDS and PCORnet Sites That Do Not Participate in PKIDS **eFigure 10.** Distribution of Sex of PKIDS Trial Participants and at PCORnet Sites That Participate in PKIDS and PCORnet Sites That Do Not Participate in PKIDS **eFigure 11.** Distribution of Race of PKIDS Trial Participants and at PCORnet Sites That Participate in PKIDS and PCORnet Sites That Do Not Participate in PKIDS **eFigure 12.** Distribution of Ethnicity of PKIDS Trial Participants and at PCORnet Sites That Participate in PKIDS and PCORnet Sites That Do Not Participate in PKIDS **eTable 1.** Variables Included in the Multiple Imputation Analyses **eTable 2.** Characteristics of Urologists Practicing in the PKIDS Trial **eTable 3.** Characteristics of Medical Centers Participating in the PKIDS Trial **eTable 4.** Standardized Mean Differences of Patient, Surgeon, and Health System Characteristics Between Ureteroscopy and Shockwave Lithotripsy Treatment Groups Before and After Propensity Score Weighting **eTable 5.** Proportion of Participants Undergoing Ureteroscopy and Shockwave Lithotripsy Whose Difference in Score at 1 Week Exceeded 30% and 50% of the SD of the Patient-Reported Outcome Measure Among All Participants at Baseline **eTable 6.** Frequency of Unanticipated Postoperative Health Care Encounters After Ureteroscopy and Shockwave Lithotripsy **eTable 7.** Stone Clearance 4 to 8 Weeks After Ureteroscopy and Shockwave Lithotripsy, Overall and by Stone Size and Stone Location **eTable 8.** Complete Data Analysis of the Impact of Ureteroscopy Compared to Shockwave Lithotripsy on Patient-Reported Outcomes of Physical, Emotional, and Social Health at 1 Week After Surgery **eTable 9.** Stone Clearance Up to 16 Weeks After Ureteroscopy and Shockwave Lithotripsy, Overall and by Stone Size and Stone Location **eTable 10.** Results of Sensitivity Analyses With Complete Data and Replacing Missing Data for Stone Clearance With Fixed Values **eTable 11.** Stone Clearance Excluding Patients Aged 19 to 21 Years **eTable 12.** Stone Clearance Excluding Patients With Renal Anomalies **eTable 13.** Stone Clearance for Ureteroscopy and Shockwave Lithotripsy at 4 to 8 Weeks After Surgery, Overall and by Stone Size and Stone Location, Incorporating the Sensitivity and Specificity of Local Ultrasonography Interpretations Determined by Central Review of a 10% Sample **eTable 14.** Results of Analyses Replacing Missing Stone Clearance With a Range of Values That Are Nondifferential Across Ureteroscopy and Shockwave Lithotripsy **eTable 15.** Results of Analyses Replacing Missing Stone Clearance With a Range of Values That Are Differential Across Ureteroscopy and Shockwave Lithotripsy **eTable 16.** Results of Analyses Comparing Patient-Reported Outcomes Between Ureteroscopy and Shockwave Lithotripsy, Excluding Patients Who Had a Ureteral Stent Placed at Time of Index Surgery **eTable 17.** Characteristics of Demographics and Affected Body Regions, Defined by the Pediatric Medical Complexity Algorithm, of Patients Aged 8 to 21 Years Who Had Ureteroscopy or Shockwave Lithotripsy During the Same Period at PKIDS Sites and Non-PKIDS Sites in PCORnet

Supplement 2.
Trial Protocol and Statistical Analysis Plan

Supplement 3.
Data Sharing Statement

## References

[1] Routh JC, Graham DA, Nelson CP. Epidemiological trends in pediatric urolithiasis at United States freestanding pediatric hospitals. J Urol. 2010;184(3):1100-1104. doi:10.1016/j.juro.2010.05.018 20650479

[2] Tasian GE, Ross ME, Song L, . Annual incidence of nephrolithiasis among children and adults in South Carolina from 1997 to 2012. Clin J Am Soc Nephrol. 2016;11(3):488-496. doi:10.2215/CJN.07610715 26769765 PMC4791823

[3] Routh JC, Graham DA, Nelson CP. Trends in imaging and surgical management of pediatric urolithiasis at American pediatric hospitals. J Urol. 2010;184(4)(suppl):1816-1822. doi:10.1016/j.juro.2010.03.117 20728146

[4] Harper JD, Desai AC, Maalouf NM, . Risk factors for increased stent-associated symptoms following ureteroscopy for urinary stones: results from STENTS. J Urol. 2023;209(5):971-980. doi:10.1097/JU.0000000000003183 36648152 PMC10336697

[5] Tasian GE, Maltenfort MG, Rove K, . Ureteral stent placement prior to definitive stone treatment is associated with higher postoperative emergency department visits and opioid prescriptions for youth having ureteroscopy or shock wave lithotripsy. J Urol. 2023;209(6):1194-1201. doi:10.1097/JU.0000000000003389 36812398

[6] Lovegrove CE, Geraghty RM, Yang B, . Natural history of small asymptomatic kidney and residual stones over a long-term follow-up: systematic review over 25 years. BJU Int. 2022;129(4):442-456. doi:10.1111/bju.15522 34157218

[7] Lombardo R, Tzelves L, Geraghty R, . Follow-up of urolithiasis patients after treatment: an algorithm from the EAU Urolithiasis Panel. World J Urol. 2024;42(1):202. doi:10.1007/s00345-024-04872-y 38546854

[8] Assimos D, Krambeck A, Miller NL, . Surgical management of stones: American Urological Association/Endourological Society guideline, part I. J Urol. 2016;196(4):1153-1160. doi:10.1016/j.juro.2016.05.090 27238616

[9] Türk C, Petřík A, Sarica K, . EAU guidelines on interventional treatment for urolithiasis. Eur Urol. 2016;69(3):475-482. doi:10.1016/j.eururo.2015.07.041 26344917

[10] Barreto L, Jung JH, Abdelrahim A, Ahmed M, Dawkins GPC, Kazmierski M. Medical and surgical interventions for the treatment of urinary stones in children. Cochrane Database Syst Rev. 2019;10(10):CD010784. doi:10.1002/14651858.CD010784.pub3 31596944 PMC6785002

[11] Ward JB, Feinstein L, Pierce C, ; NIDDK Urologic Diseases in America Project. Pediatric urinary stone disease in the United States: the Urologic Diseases in America Project. Urology. 2019;129:180-187. doi:10.1016/j.urology.2019.04.012 31005657 PMC6988134

[12] Freton L, Peyronnet B, Arnaud A, . Extracorporeal shockwave lithotripsy versus flexible ureteroscopy for the management of upper tract urinary stones in children. J Endourol. 2017;31(1):1-6. doi:10.1089/end.2016.0313 27824261

[13] Irwin DE, Gross HE, Stucky BD, . Development of six PROMIS pediatrics proxy-report item banks. Health Qual Life Outcomes. 2012;10:22. doi:10.1186/1477-7525-10-22 22357192 PMC3312870

[14] Hardin AP, Hackell JM; Committee on Practice and Ambulatory Medicine. Age limit of pediatrics. Pediatrics. 2017;140(3):e20172151. doi:10.1542/peds.2017-2151 28827380

[15] Fernandez N, Ellison JS, Wang Z, . Surgeon, and institution characteristics associated surgical preferences in the Pediatric KIDney Stone Care Improvement Network. Urology. 2024;187:64-70. doi:10.1016/j.urology.2024.02.040 38458327

[16] Fazel M, Gubari MIM, Yousefifard M, Hosseini M. Ultrasonography in detection of renal calculi in children: a systematic review and meta-analysis. Arch Acad Emerg Med. 2019;7(1):e66. 32021977 PMC6942918

[17] Grivas N, Thomas K, Drake T, . Imaging modalities and treatment of paediatric upper tract urolithiasis: a systematic review and update on behalf of the EAU Urolithiasis Guidelines Panel. J Pediatr Urol. 2020;16(5):612-624. doi:10.1016/j.jpurol.2020.07.003 32739360

[18] Dunmire B, Lee FC, Hsi RS, . Tools to improve the accuracy of kidney stone sizing with ultrasound. J Endourol. 2015;29(2):147-152. doi:10.1089/end.2014.0332 25105243 PMC4313404

[19] Dunmire B, Harper JD, Cunitz BW, . Use of the acoustic shadow width to determine kidney stone size with ultrasound. J Urol. 2016;195(1):171-177. doi:10.1016/j.juro.2015.05.111 26301788 PMC4821497

[20] Raman JD, Bagrodia A, Gupta A, . Natural history of residual fragments following percutaneous nephrostolithotomy. J Urol. 2009;181(3):1163-1168. doi:10.1016/j.juro.2008.10.162 19152935

[21] Iremashvili V, Li S, Penniston KL, Best SL, Hedican SP, Nakada SY. Role of residual fragments on the risk of repeat surgery after flexible ureteroscopy and laser lithotripsy: single center study. J Urol. 2019;201(2):358-363. doi:10.1016/j.juro.2018.09.053 30273609

[22] Hung M, Stuart AR, Higgins TF, Saltzman CL, Kubiak EN. Computerized adaptive testing using the PROMIS physical function item bank reduces test burden with less ceiling effects compared with the short musculoskeletal function assessment in orthopaedic trauma patients. J Orthop Trauma. 2014;28(8):439-443. doi:10.1097/BOT.0000000000000059 24378399

[23] Revicki DA, Cella DF. Health status assessment for the twenty-first century: item response theory, item banking and computer adaptive testing. Qual Life Res. 1997;6(6):595-600. doi:10.1023/A:1018420418455 9330558

[24] Recommendations by age. Healthmeasures. Accessed March 22, 2024. https://www.healthmeasures.net/explore-measurement-systems/selecting-among-measurement-systems/recommendations-by-age

[25] Varni JW, Stucky BD, Thissen D, . PROMIS Pediatric Pain Interference Scale: an item response theory analysis of the pediatric pain item bank. J Pain. 2010;11(11):1109-1119. doi:10.1016/j.jpain.2010.02.005 20627819 PMC3129595

[26] Cella D, Riley W, Stone A, ; PROMIS Cooperative Group. The Patient-Reported Outcomes Measurement Information System (PROMIS) developed and tested its first wave of adult self-reported health outcome item banks: 2005-2008. J Clin Epidemiol. 2010;63(11):1179-1194. doi:10.1016/j.jclinepi.2010.04.011 20685078 PMC2965562

[27] Farhat W, Bägli DJ, Capolicchio G, . The dysfunctional voiding scoring system: quantitative standardization of dysfunctional voiding symptoms in children. J Urol. 2000;164(3 Pt 2):1011-1015. doi:10.1016/S0022-5347(05)67239-4 10958730

[28] PROMIS instrument development and validation scientific standards, version 2.0. Healthmeasures. Revised May 2013. Accessed July 21, 2025. https://www.healthmeasures.net/images/PROMIS/PROMISStandards_Vers2.0_Final.pdf

[29] Dogan HS, Altan M, Citamak B, Bozaci AC, Karabulut E, Tekgul S. A new nomogram for prediction of outcome of pediatric shock-wave lithotripsy. J Pediatr Urol. 2015;11(2):84.e1-84.e6. doi:10.1016/j.jpurol.2015.01.004 25812469

[30] Terwee CB, Peipert JD, Chapman R, . Minimal important change (MIC): a conceptual clarification and systematic review of MIC estimates of PROMIS measures. Qual Life Res. 2021;30(10):2729-2754. doi:10.1007/s11136-021-02925-y 34247326 PMC8481206

[31] Norman GR, Sloan JA, Wyrwich KW. Interpretation of changes in health-related quality of life: the remarkable universality of half a standard deviation. Med Care. 2003;41(5):582-592. doi:10.1097/01.MLR.0000062554.74615.4C 12719681

[32] Simon TD, Cawthon ML, Stanford S, ; Center of Excellence on Quality of Care Measures for Children with Complex Needs (COE4CCN) Medical Complexity Working Group. Pediatric medical complexity algorithm: a new method to stratify children by medical complexity. Pediatrics. 2014;133(6):e1647-e1654. doi:10.1542/peds.2013-3875 24819580 PMC4035595

[33] Simon TD, Haaland W, Hawley K, Lambka K, Mangione-Smith R. Development and validation of the Pediatric Medical Complexity Algorithm (PMCA) version 3.0. Acad Pediatr. 2018;18(5):577-580. doi:10.1016/j.acap.2018.02.010 29496546 PMC6035108

[34] Ellison JS, Lorenzo M, Beck H, ; Pediatric Kidney Stone Care Improvement Network. Comparative effectiveness of paediatric kidney stone surgery (the PKIDS trial): study protocol for a patient-centred pragmatic clinical trial. BMJ Open. 2022;12(4):e056789. doi:10.1136/bmjopen-2021-056789 35383073 PMC8983998

[35] Ishii H, Griffin S, Somani BK. Ureteroscopy for stone disease in the paediatric population: a systematic review. BJU Int. 2015;115(6):867-873. doi:10.1111/bju.12927 25203925

[36] Kim HJ, Daignault-Newton S, DiBianco JM, ; Michigan Urological Surgery Improvement Collaborative. Real-world practice stone-free rates after ureteroscopy: variation and outcomes in a surgical collaborative. Eur Urol Focus. 2023;9(5):773-780. doi:10.1016/j.euf.2023.03.010 37031097

[37] Elsobky E, Sheir KZ, Madbouly K, Mokhtar AA. Extracorporeal shock wave lithotripsy in children: experience using two second-generation lithotripters. BJU Int. 2000;86(7):851-856. doi:10.1046/j.1464-410x.2000.00899.x 11069413

[38] Harper JD, Desai AC, Antonelli JA, ; NIDDK Urinary Stone Disease Research Network (USDRN). Quality of life impact and recovery after ureteroscopy and stent insertion: insights from daily surveys in STENTS. BMC Urol. 2022;22(1):53. doi:10.1186/s12894-022-01004-9 35387623 PMC8988384

[39] Ahmed AF, Shalaby E, Maarouf A, Badran Y, Eladl M, Ghobish A. Diuresis and inversion therapy to improve clearance of lower caliceal stones after shock wave lithotripsy: A prospective, randomized, controlled, clinical study. Indian J Urol. 2015;31(2):125-131. doi:10.4103/0970-1591.152813 25878414 PMC4397549

[40] Brain E, Geraghty RM, Lovegrove CE, Yang B, Somani BK. Natural history of post-treatment kidney stone fragments: a systematic review and meta-analysis. J Urol. 2021;206(3):526-538. doi:10.1097/JU.0000000000001836 33904756

