# Supplementary material for: Ureteroscopy vs Shockwave Lithotripsy to Remove Kidney Stones in Children and Adolescents: A Nonrandomized Clinical Trial
Source: JAMA Netw Open. 2025 Aug 7;8(8):e2525789. doi: 10.1001/jamanetworkopen.2025.25789 (PMC12332628; doi:10.1001/jamanetworkopen.2025.25789)
Supplement: Supplement 1. — eAppendix 1. PKIDS Standardized Ultrasonography Protocol eAppendix 2. PKIDS Ultrasonography Report eAppendix 3. Questionnaire for Urinary Issues—Kidney Stone Surgery (QUIKSS) eFigure 1. Pediatric KIDney Stone Care Improvement Network eFigure 2. Balance of Patient, Surgeon, and Health System Characteristics Between URS and SWL Treatment Groups Before and After Propensity Score Weighting eFigure 3. Difference in Stone Clearance Between URS and SWL With Preoperative Largest Stone Size Estimated as a Continuous Variable eFigure 4. Impact of URS and SWL on Recovery of Physical, Emotional, and Social Health eFigure 5. Impact of URS and SWL on Patient-Reported Outcomes of Physical, Emotional, and Social Health After Surgery, by Age Group at Baseline (BL) and to 3 Months After Surgery eFigure 6. Impact of URS and SWL on Patient-Reported Outcomes of Physical, Emotional, and Social Health After Surgery, by Sex Group at Baseline and to 3 Months After Surgery eFigure 7. Results of Multiple Imputation Analyses Replacing Missing Stone Clearance With a Range of Values That Are Nondifferential Across URS and SWL eFigure 8. Results of Multiple Imputation Analyses Replacing Missing Stone Clearance With a Range of Values That Are Differential Across URS and SWL eFigure 9. Distribution of Age of PKIDS Trial Participants and at PCORnet Sites That Participate in PKIDS and PCORnet Sites That Do Not Participate in PKIDS eFigure 10. Distribution of Sex of PKIDS Trial Participants and at PCORnet Sites That Participate in PKIDS and PCORnet Sites That Do Not Participate in PKIDS eFigure 11. Distribution of Race of PKIDS Trial Participants and at PCORnet Sites That Participate in PKIDS and PCORnet Sites That Do Not Participate in PKIDS eFigure 12. Distribution of Ethnicity of PKIDS Trial Participants and at PCORnet Sites That Participate in PKIDS and PCORnet Sites That Do Not Participate in PKIDS eTable 1. Variables Included in the Multiple Imputation Analyses eTable 2. Characteristics of Urolo [file jamanetwopen-e2525789-s001.pdf]

Supplemental Online Content

Tasian GE, Chu DI, Nelson CP, et al. Ureteroscopy vs shockwave lithotripsy to remove kidney stones in children and adolescents: a nonrandomized clinical trial. *JAMA Netw Open*. 2025;8(8):e2525789. doi:10.1001/jamanetworkopen.2025.25789

eAppendix 1. PKIDS Standardized Ultrasound Protocol
eAppendix 2. PKIDS Ultrasound Report
eAppendix 3. Questionnaire for Urinary Issues—Kidney Stone Surgery (QUIKSS)
eFigure 1. Pediatric KIDney Stone Care Improvement Network
eFigure 2. Balance of Patient, Surgeon, and Health System Characteristics Between URS and SWL Treatment Groups Before and After Propensity Score Weighting
eFigure 3. Difference in Stone Clearance Between URS and SWL With Preoperative Largest Stone Size Estimated as a Continuous Variable
eFigure 4. Impact of URS and SWL on Recovery of Physical, Emotional, and Social Health
eFigure 5. Impact of URS and SWL on Patient-Reported Outcomes of Physical, Emotional, and Social Health After Surgery, by Age Group at Baseline (BL) and to 3 Months After Surgery
eFigure 6. Impact of URS and SWL on Patient-Reported Outcomes of Physical, Emotional, and Social Health After Surgery, by Sex Group at Baseline and to 3 Months After Surgery
eFigure 7. Results of Multiple Imputation Analyses Replacing Missing Stone Clearance With a Range of Values That Are Nondifferential Across URS and SWL
eFigure 8. Results of Multiple Imputation Analyses Replacing Missing Stone Clearance With a Range of Values That Are Differential Across URS and SWL
eFigure 9. Distribution of Age of PKIDS Trial Participants and at PCORnet Sites That Participate in PKIDS and PCORnet Sites That Do Not Participate in PKIDS
eFigure 10. Distribution of Sex of PKIDS Trial Participants and at PCORnet Sites That Participate in PKIDS and PCORnet Sites That Do Not Participate in PKIDS
eFigure 11. Distribution of Race of PKIDS Trial Participants and at PCORnet Sites That Participate in PKIDS and PCORnet Sites That Do Not Participate in PKIDS
eFigure 12. Distribution of Ethnicity of PKIDS Trial Participants and at PCORnet Sites That Participate in PKIDS and PCORnet Sites That Do Not Participate in PKIDS
eTable 1. Variables Included in the Multiple Imputation Analyses
eTable 2. Characteristics of Urologists Practicing in the PKIDS Trial
eTable 3. Characteristics of Medical Centers Participating in the PKIDS Trial

© 2025 Tasian GE et al. *JAMA Network Open*.

eTable 4. Standardized Mean Differences of Patient, Surgeon, and Health System Characteristics Between Ureteroscopy and Shockwave Lithotripsy Treatment Groups Before and After Propensity Score Weighting

eTable 5. Proportion of Participants Undergoing Ureteroscopy and Shockwave Lithotripsy Whose Difference in Score at 1 Week Exceeded 30% and 50% of the SD of the Patient-Reported Outcome Measure Among All Participants at Baseline

eTable 6. Frequency of Unanticipated Postoperative Health Care Encounters After Ureteroscopy and Shockwave Lithotripsy

eTable 7. Stone Clearance 4 to 8 Weeks After Ureteroscopy and Shockwave Lithotripsy, Overall and by Stone Size and Stone Location

eTable 8. Complete Data Analysis of the Impact of Ureteroscopy Compared to Shockwave Lithotripsy on Patient-Reported Outcomes of Physical, Emotional, and Social Health at 1 Week After Surgery

eTable 9. Stone Clearance Up to 16 Weeks After Ureteroscopy and Shockwave Lithotripsy, Overall and by Stone Size and Stone Location

eTable 10. Results of Sensitivity Analyses With Complete Data and Replacing Missing Data for Stone Clearance With Fixed Values

eTable 11. Stone Clearance Excluding Patients Aged 19 to 21 Years

eTable 12. Stone Clearance Excluding Patients With Renal Anomalies

eTable 13. Stone Clearance for Ureteroscopy and Shockwave Lithotripsy at 4 to 8 Weeks After Surgery, Overall and by Stone Size and Stone Location, Incorporating the Sensitivity and Specificity of Local Ultrasound Interpretations Determined by Central Review of a 10% Sample

eTable 14. Results of Analyses Replacing Missing Stone Clearance With a Range of Values That Are Nondifferential Across Ureteroscopy and Shockwave Lithotripsy

eTable 15. Results of Analyses Replacing Missing Stone Clearance With a Range of Values That Are Differential Across Ureteroscopy and Shockwave Lithotripsy

eTable 16. Results of Analyses Comparing Patient-Reported Outcomes Between Ureteroscopy and Shockwave Lithotripsy, Excluding Patients Who Had a Ureteral Stent Placed at Time of Index Surgery

eTable 17. Characteristics of Demographics and Affected Body Regions, Defined by the Pediatric Medical Complexity Algorithm, of Patients Aged 8 to 21 Years Who Had Ureteroscopy or Shockwave Lithotripsy During the Same Period at PKIDS Sites and Non-PKIDS Sites in PCORnet

This supplemental material has been provided by the authors to give readers additional information about their work.

© 2025 Tasian GE et al. *JAMA Network Open*.

eAppendix 1. PKIDS Standardized Ultrasound Protocol

1. Image acquisition
    a. Imaging specifications
        i. Image acquisition
            1. Static image
            2. Dynamic clips (this is missing but important for quality and follow-up – we will need to train this)
        ii. Patient positioning
            1. Supine
            2. Prone
    b. Scanning for stones
        i. Setting
            1. Mode: harmonic
            2. Depth low without at least a cm soft tissue posterior to the kidney (so it is possible to see dorsal acoustic shadow and also twinkling artifact)
            3. Zoom (maximum without cutting the upper and lower poles
            4. Focus low
        ii. Planes
            1. Longitudinal: anterior, middle, and posterior
            2. Axial: upper, middle, and lower
    c. Stone identification and measurement (if stone identified on scanning)
        i. Settings
            1. Mode: Grayscale and harmonic
            2. Focus moved to stone
            3. Zoom
        ii. Diagnostic criteria (both necessary)
            1. Echogenic focus on grayscale and harmonic
            2. Twinkle artifact with focus on echogenic focus (maximum pulse repetition frequency for each transducer)
        iii. Measurement
            1. Measure at distinct, most echogenic boundaries of stone
            2. Obtain images of the stone with and without measurement
            3. Planes of measurement (have these static images side by side)
                a. Sagittal
                    i. Longitudinal axis of stone (mm)
                    ii. Horizontal axis of stone (mm)
                b. Transverse
                    i. Horizontal axis of stone (mm)

© 2025 Tasian GE et al. *JAMA Network Open*.

eAppendix 2. PKIDS Ultrasound Report

CLINICAL HISTORY: [ ]

EXAMINATION: [ ]

DATE: [ ]

COMPARISON: [ ]

TECHNIQUE: Grayscale sonographic image acquisition was performed of the kidneys and bladder.

FINDINGS:

BLADDER:
Stone visualized at UVJ: [No/Yes]

RIGHT KIDNEY:
Collecting system: [The calyces and pelvis are nondilated.]
Ureter: [The ureter is nondilated.]
Calculi: [No/Yes]
- If calculus present, measure the longest dimension of 3 largest stones

1. Location: [If present, PLEASE DESCRIBE for each measured stone: e.g., upper pole calyx; interpolar calyx; lower pole calyx; renal pelvis; ureteropelvic junction; proximal ureter; distal ureter]
- Size: [ ] mm
- Shadow: [No/Yes]
- Doppler: [Not applicable/Twinkle/No twinkle]
2. Location: [If present, PLEASE DESCRIBE for each measured stone: e.g., upper pole calyx; interpolar calyx; lower pole calyx; renal pelvis; ureteropelvic junction; proximal ureter; distal ureter]
- Size: [ ] mm
- Shadow: [No/Yes]
- Doppler: [Not applicable/Twinkle/No twinkle]
3. Location: [If present, PLEASE DESCRIBE for each measured stone: e.g., upper pole calyx; interpolar calyx; lower pole calyx; renal pelvis; ureteropelvic junction; proximal ureter; distal ureter]
- Size: [ ] mm
- Shadow: [No/Yes]
- Doppler: [Not applicable/Twinkle/No twinkle]

LEFT KIDNEY:
Collecting system: [The calyces and pelvis are nondilated.]
Ureter: [The ureter is nondilated.]
Calculi: [No/Yes]
- If calculus present, measure the longest dimension of 3 largest stones

1. Location: [If present, PLEASE DESCRIBE for each measured stone: e.g., upper pole calyx; interpolar calyx; lower pole calyx; renal pelvis; ureteropelvic junction; proximal ureter; distal ureter]
- Size: [ ] mm
- Shadow: [No/Yes]
- Doppler: [Not applicable/Twinkle/No twinkle]
2. Location: [If present, PLEASE DESCRIBE for each measured stone: e.g., upper pole calyx; interpolar calyx; lower pole calyx; renal pelvis; ureteropelvic junction; proximal ureter; distal ureter]
- Size: [ ] mm
- Shadow: [No/Yes]

© 2025 Tasian GE et al. *JAMA Network Open*.

- Doppler: [Not applicable/Twinkle/No twinkle]
3. Location: [If present, PLEASE DESCRIBE for each measured stone: e.g., upper pole calyx; interpolar calyx; lower pole calyx; renal pelvis; ureteropelvic junction; proximal ureter; distal ureter]
- Size: [ ] mm
- Shadow: [No/Yes]
- Doppler: [Not applicable/Twinkle/No twinkle]

IMPRESSION:

© 2025 Tasian GE et al. *JAMA Network Open*.

eAppendix 3. Questionnaire for Urinary Issues—Kidney Stone Surgery (QUIKSS)

QUIKSS

Please answer the following questions, thinking about your experience over the past 7 days:

	QUIKSS	Never	Almost Never	Sometimes	Often	Almost always
1)	I had blood in my pee.	○	○	○	○	○
2)	It hurt when I peed.	○	○	○	○	○
3)	I peed more than usual.	○	○	○	○	○
4)	I had to pee a lot.	○	○	○	○	○
5)	I rushed to the bathroom because I had to pee.	○	○	○	○	○
6)	I woke up at night to pee.	○	○	○	○	○
7)	I could not pee even when I tried.	○	○	○	○	○
8)	I felt there was more urine in my bladder even after I peed.	○	○	○	○	○
9)	I felt like I had urine left in my bladder after I peed.	○	○	○	○	○
10)	I felt like there was more urine in my bladder after I stopped peeing.	○	○	○	○	○
11)	I peed in my underwear.	○	○	○	○	○
12)	I had trouble peeing.	○	○	○	○	○
13)	I had difficulty peeing.	○	○	○	○	○
14)	I had problems peeing.	○	○	○	○	○

15) Total Score ____________________

© 2025 Tasian GE et al. *JAMA Network Open*.

eFigure 1. Pediatric KIDney Stone Care Improvement Network

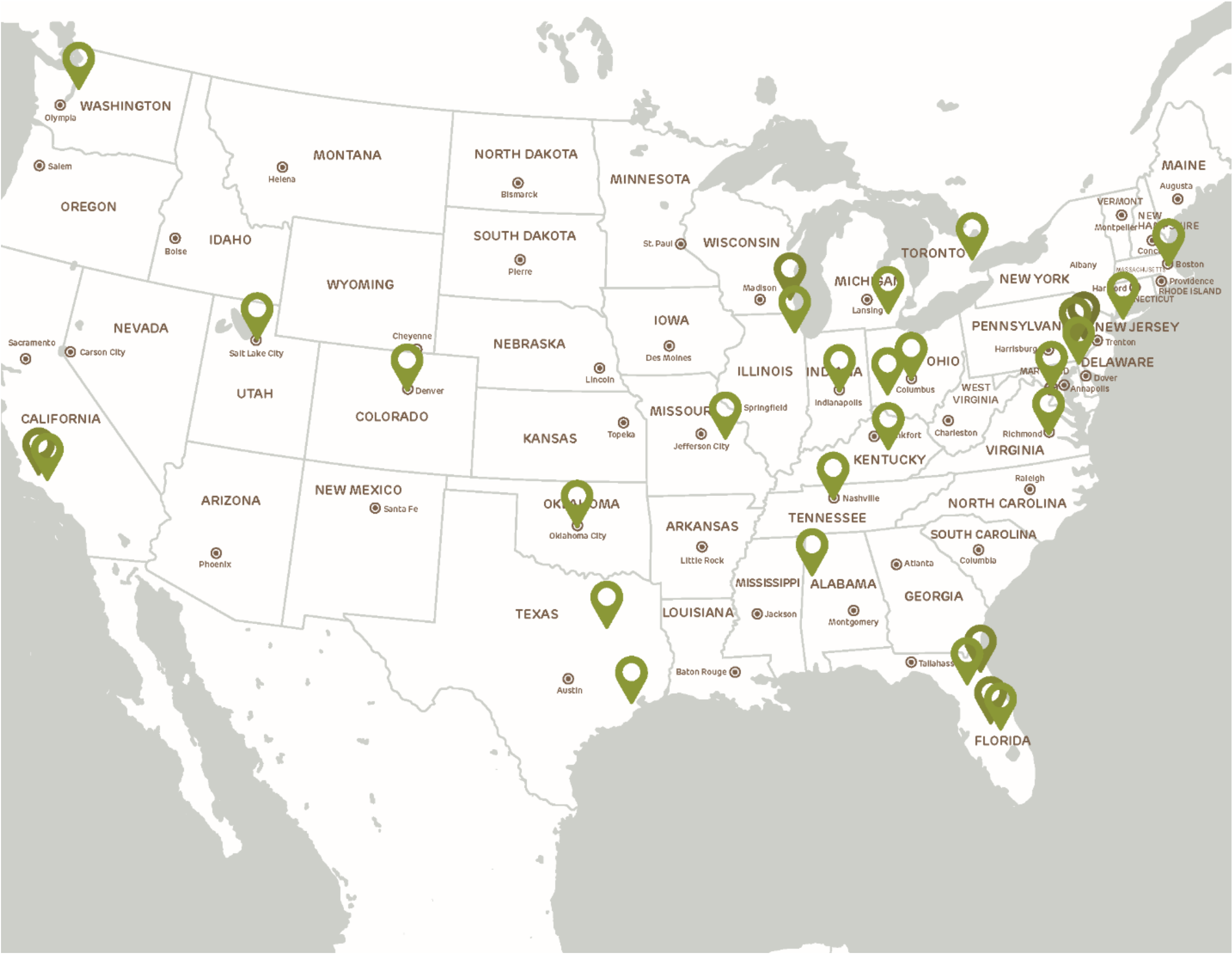

© 2025 Tasian GE et al. *JAMA Network Open*.

eFigure 2. Balance of Patient, Surgeon, And Health System Characteristics Between URS and SWL Treatment Groups Before and After Propensity Score Weighting

The standardized mean differences (SMD) of characteristics are shown before (green) and after (red) propensity score weighting.

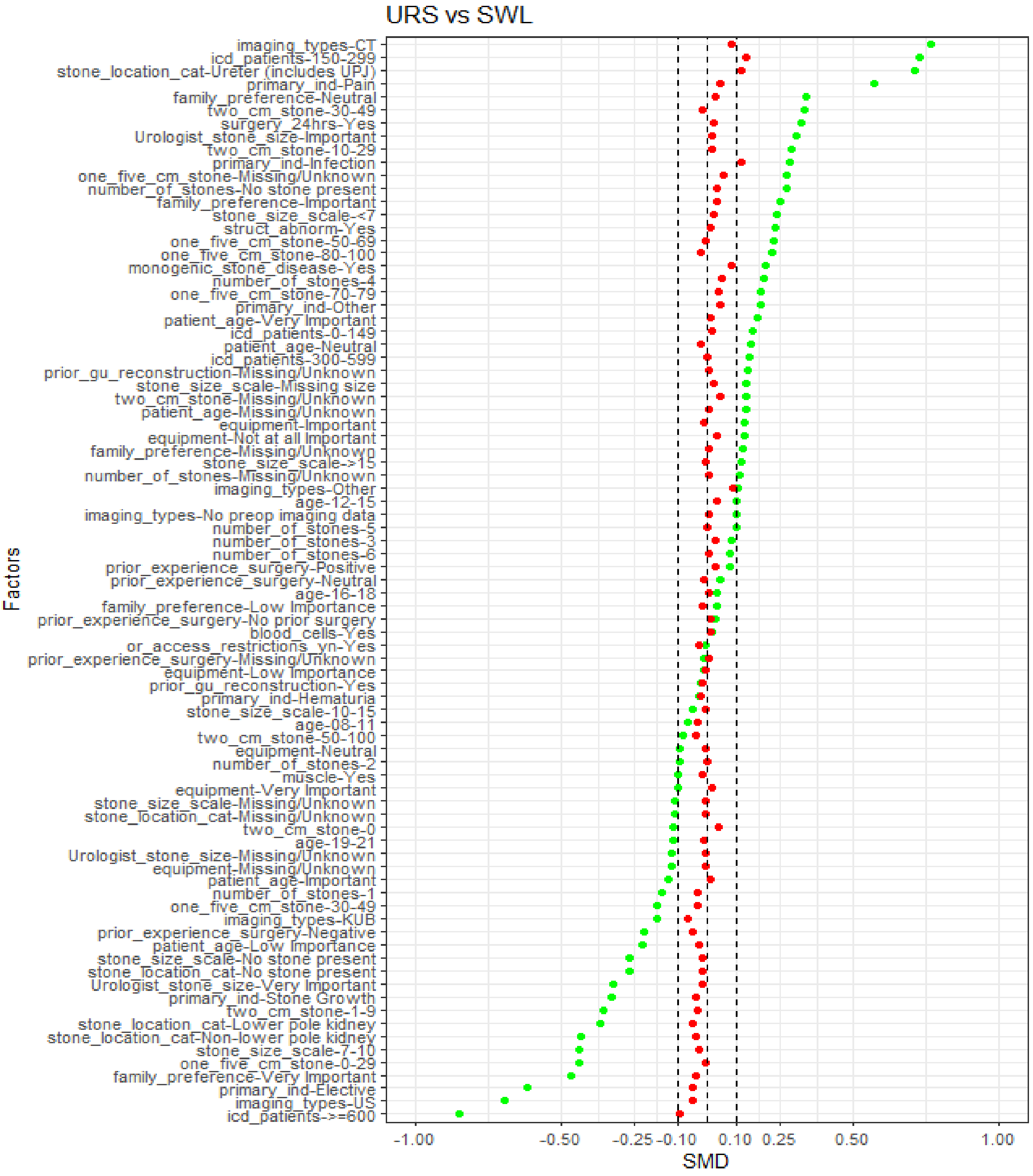

Acronyms: URS; Ureteroscopy, SWL; Shockwave Lithotripsy

© 2025 Tasian GE et al. *JAMA Network Open*.

eFigure 3. Difference in Stone Clearance Between URS and SWL With Preoperative Largest Stone Size Estimated as a Continuous Variable

95% confidence intervals are shaded areas surrounding the point estimates.

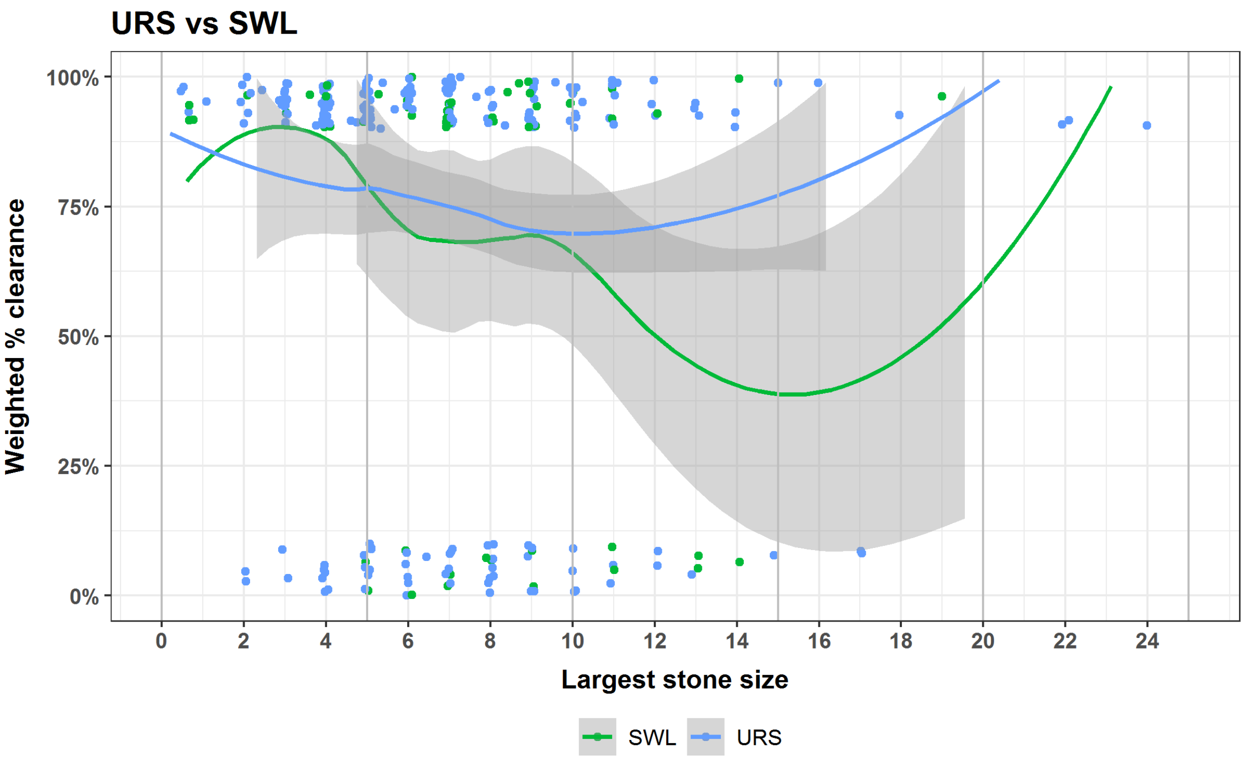

Acronyms: URS; Ureteroscopy, SWL; Shockwave Lithotripsy

© 2025 Tasian GE et al. *JAMA Network Open*.

eFigure 4. Impact of URS and SWL on Recovery of Physical, Emotional, and Social Health

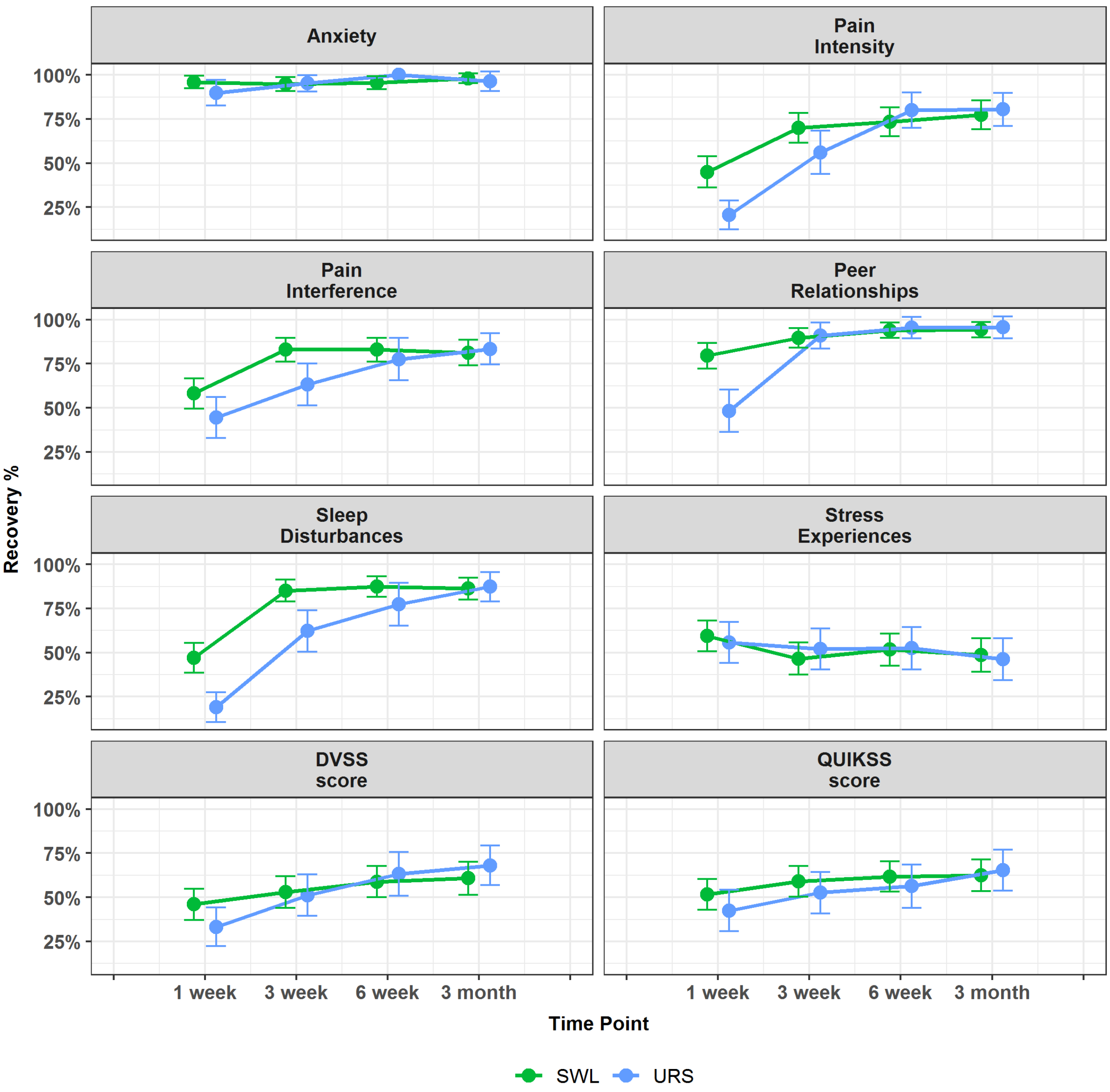

Recovery was defined as the proportion of patients at each time-point whose T-score was 50 or less for PROMIS measures and was equal to or lower than the baseline median for urinary symptoms scores. Acronyms: DVSS, Dysfunctional Voiding Symptoms Score; QUIKSS, Questionnaire for Urinary Issues – Kidney Stone; Surgery, URS; Ureteroscopy, SWL; Shockwave Lithotripsy

© 2025 Tasian GE et al. *JAMA Network Open*.

eFigure 5. Impact of URS and SWL on Patient-Reported Outcomes of Physical, Emotional, and Social Health After Surgery, by Age Group at Baseline (BL) and to 3 Months After Surgery

Individuals aged 19-21 years undergoing URS had worse urinary symptoms at one week after surgery than younger individuals undergoing URS. Higher scores equate to worse experiences, except for peer relationships.

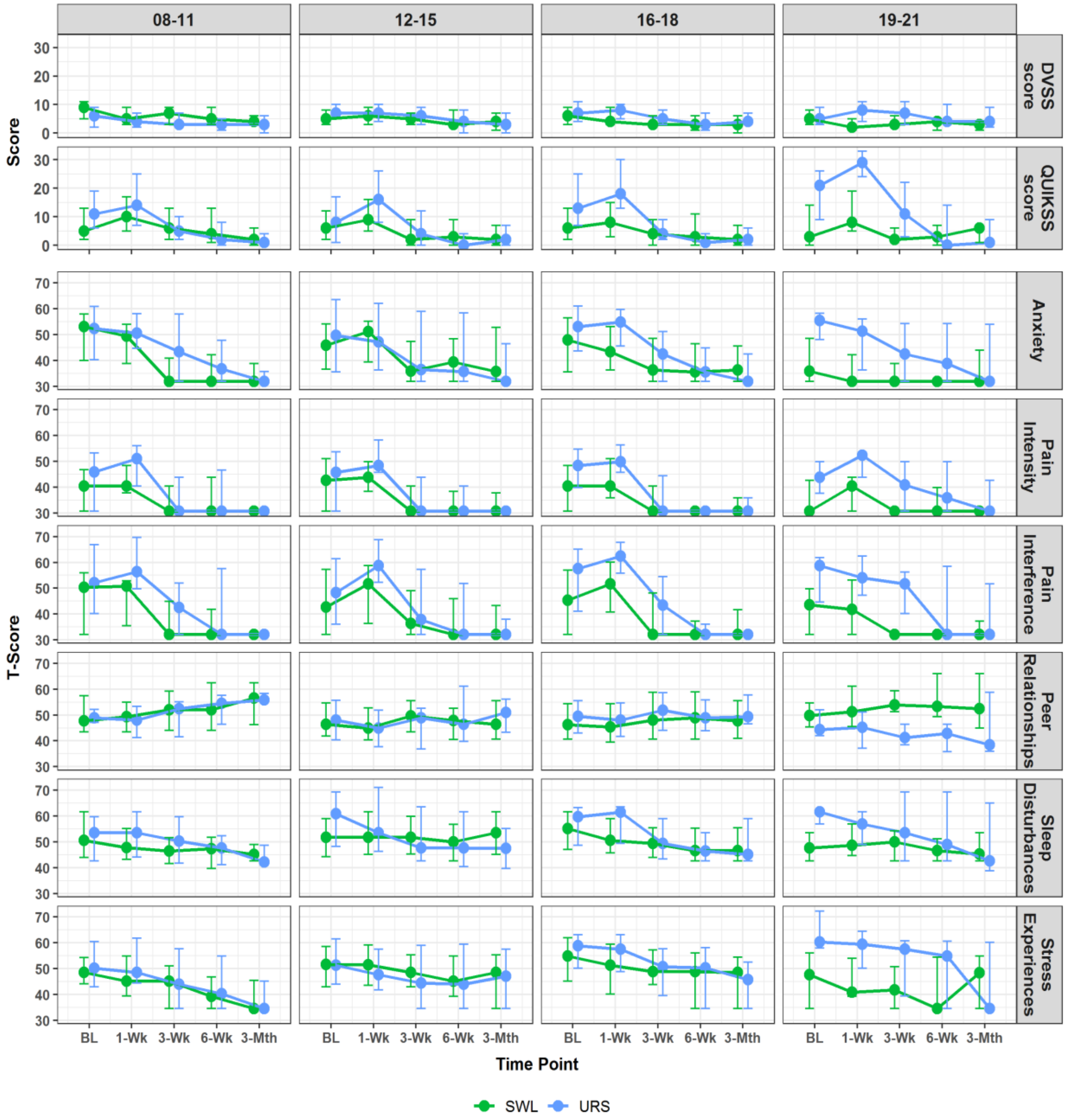

Acronyms: DVSS, Dysfunctional Voiding Symptoms Score; QUIKSS, Questionnaire for Urinary Issues – Kidney Stone; Surgery, URS; Ureteroscopy, SWL; Shockwave Lithotripsy

© 2025 Tasian GE et al. *JAMA Network Open*.

eFigure 6. Impact of URS and SWL on Patient-Reported Outcomes of Physical, Emotional, and Social Health After Surgery, by Sex Group at Baseline and to 3 Months After Surgery

Higher scores equate to worse experiences, except for peer relationships.

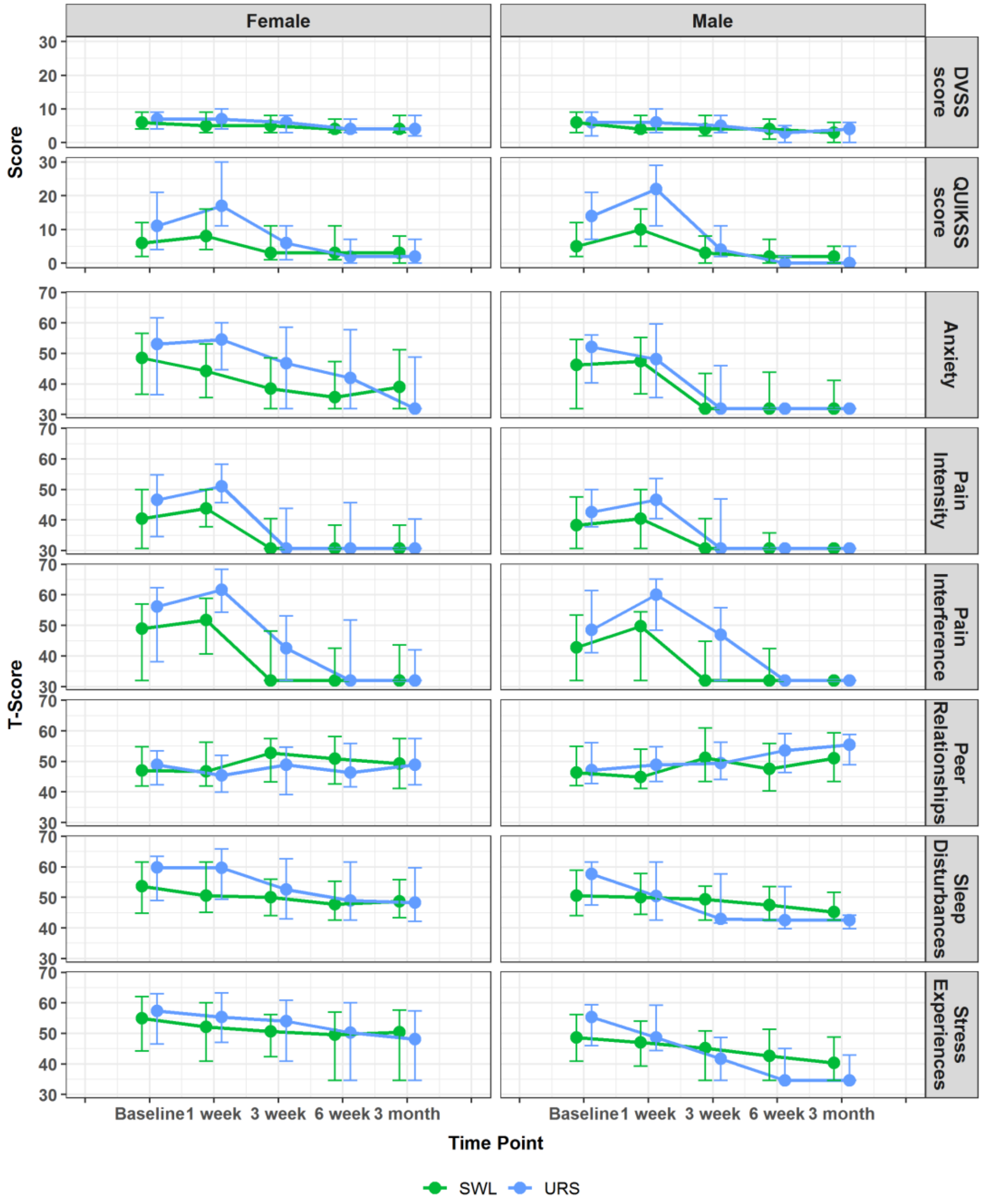

Acronyms: DVSS, Dysfunctional Voiding Symptoms Score; QUIKSS, Questionnaire for Urinary Issues – Kidney Stone; Surgery, URS; Ureteroscopy, SWL; Shockwave Lithotripsy

© 2025 Tasian GE et al. *JAMA Network Open*.

eFigure 7. Results of Multiple Imputation Analyses Replacing Missing Stone Clearance With a Range of Values That Are Nondifferential Across URS and SWL

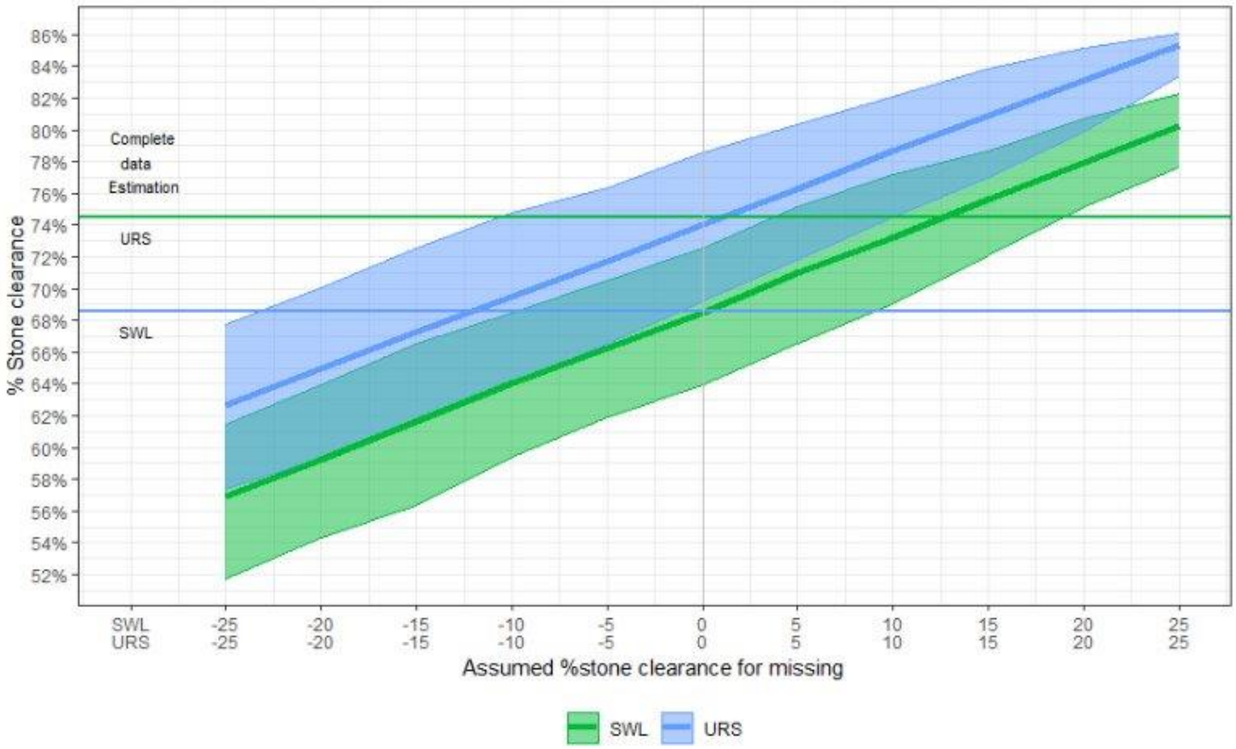

Acronyms: URS; Ureteroscopy, SWL; Shockwave Lithotripsy

eFigure 8. Results of Multiple Imputation Analyses Replacing Missing Stone Clearance With a Range of Values That Are Differential Across URS and SWL

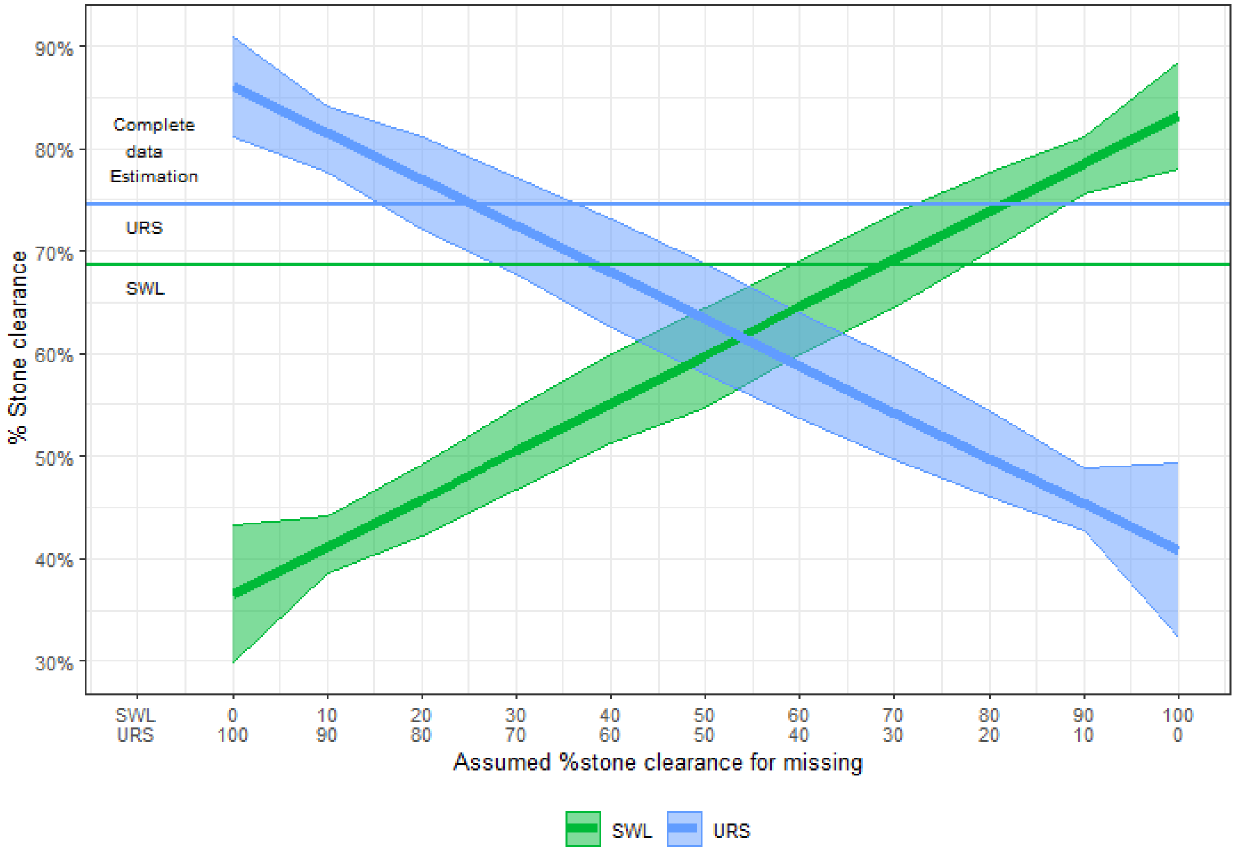

Acronyms: URS, Ureteroscopy; SWL, Shockwave Lithotripsy

© 2025 Tasian GE et al. *JAMA Network Open*.

eFigure 9. Distribution of Age of PKIDS Trial Participants (teal) and at PCORnet sites That Participate in PKIDS (orange; top) and PCORnet Sites That Do Not Participate in PKIDS (orange; bottom)

The p-value reflects the statistical significance of the difference in the distribution of age between patients aged 8 to 21 years who had ureteroscopy or shockwave lithotripsy at PKIDS study and non-PKIDS study sites in PCORnet.

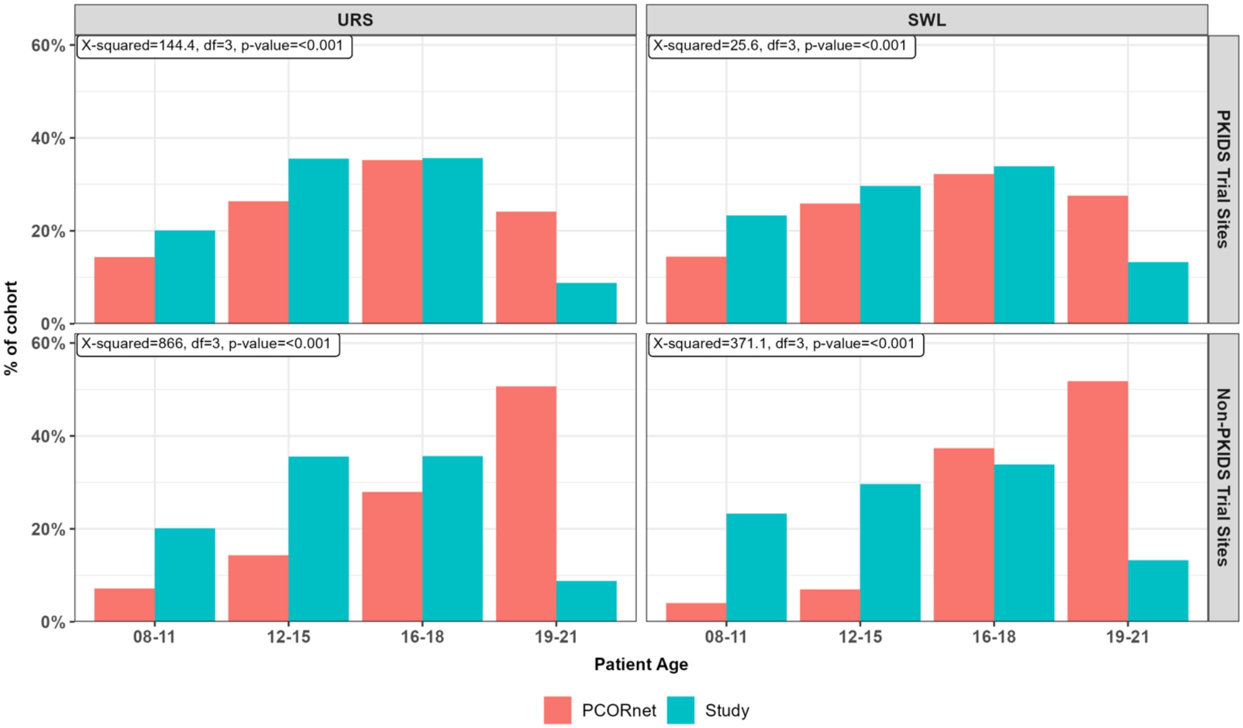

Acronyms: URS, Ureteroscopy; SWL, Shockwave Lithotripsy

© 2025 Tasian GE et al. *JAMA Network Open*.

eFigure 10. Distribution of Sex of PKIDS Trial Participants (teal) and at PCORnet Sites That Participate in PKIDS (orange; top) and PCORnet Sites That Do Not Participate in PKIDS (orange; bottom)

The p-value reflects the statistical significance of the difference in the sex distribution between patients aged 8 to 21 years who had ureteroscopy or shockwave lithotripsy at PKIDS study and non-PKIDS study sites in PCORnet.

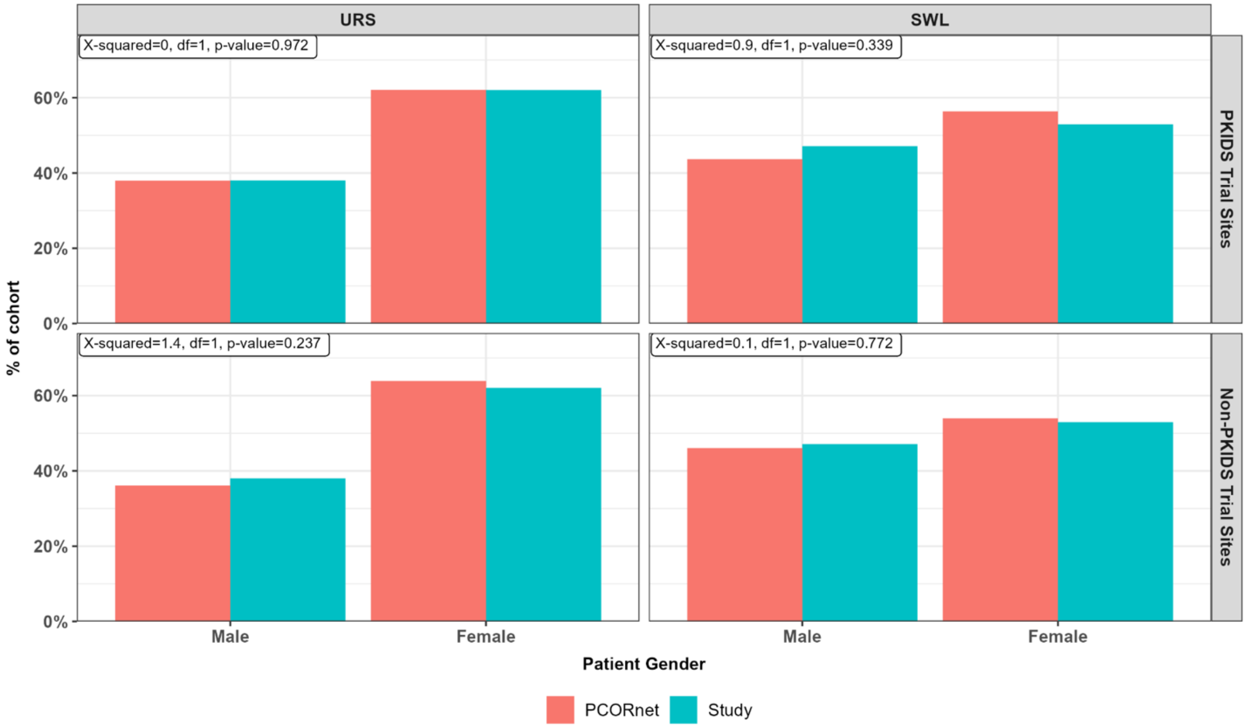

Acronyms: URS, Ureteroscopy; SWL, Shockwave Lithotripsy

© 2025 Tasian GE et al. *JAMA Network Open*.

eFigure 11. Distribution of Race of PKIDS Trial Participants (teal) and at PCORnet Sites That Participate in PKIDS (orange; top) and PCORnet Sites That Do Not Participate in PKIDS (orange; bottom)

The p-value reflects the statistical significance of the difference in the distribution of race between patients aged 8 to 21 years who had ureteroscopy or shockwave lithotripsy at PKIDS study and non-PKIDS study sites in PCORnet.

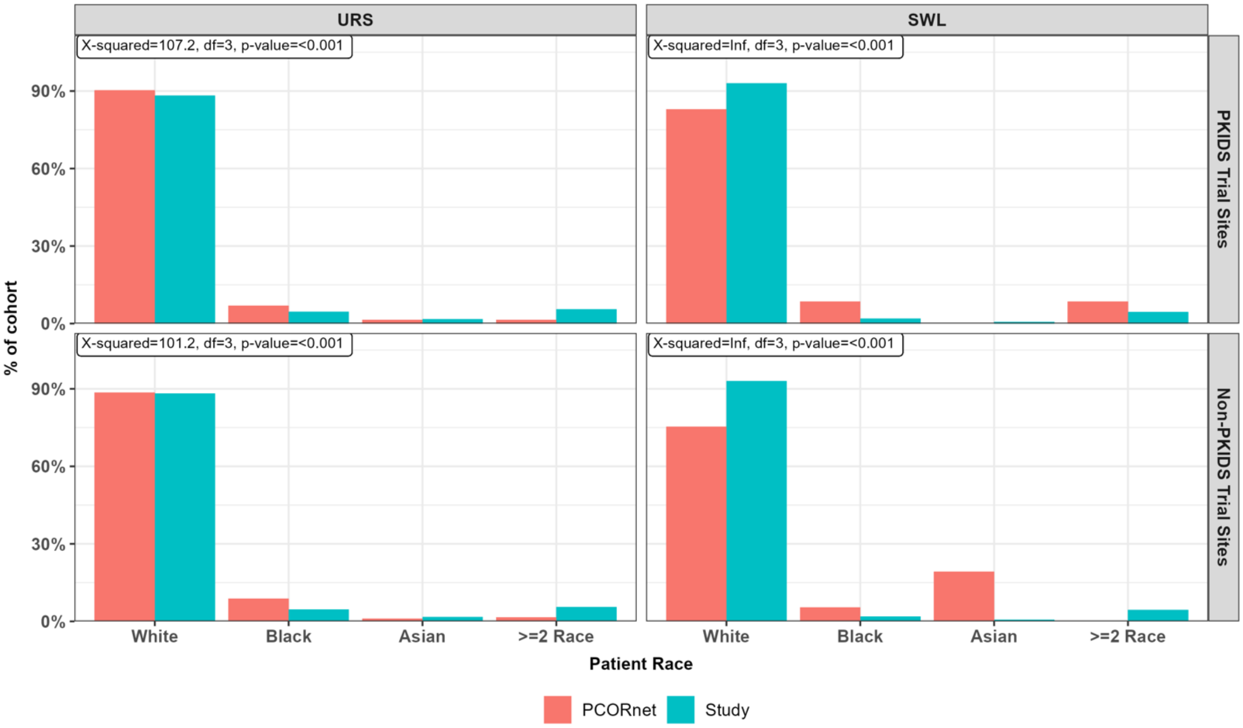

Acronyms: URS, Ureteroscopy; SWL, Shockwave Lithotripsy

© 2025 Tasian GE et al. *JAMA Network Open*.

eFigure 12. Distribution of Ethnicity of PKIDS Trial Participants (teal) and at PCORnet Sites That Participate in PKIDS (orange; top) and PCORnet Sites That Do Not Participate in PKIDS (orange; bottom)

The p-value reflects the statistical significance of the difference in the distribution of Hispanic ethnicity between patients aged 8 to 21 years who had ureteroscopy or shockwave lithotripsy at PKIDS study and non-PKIDS study sites in PCORnet.

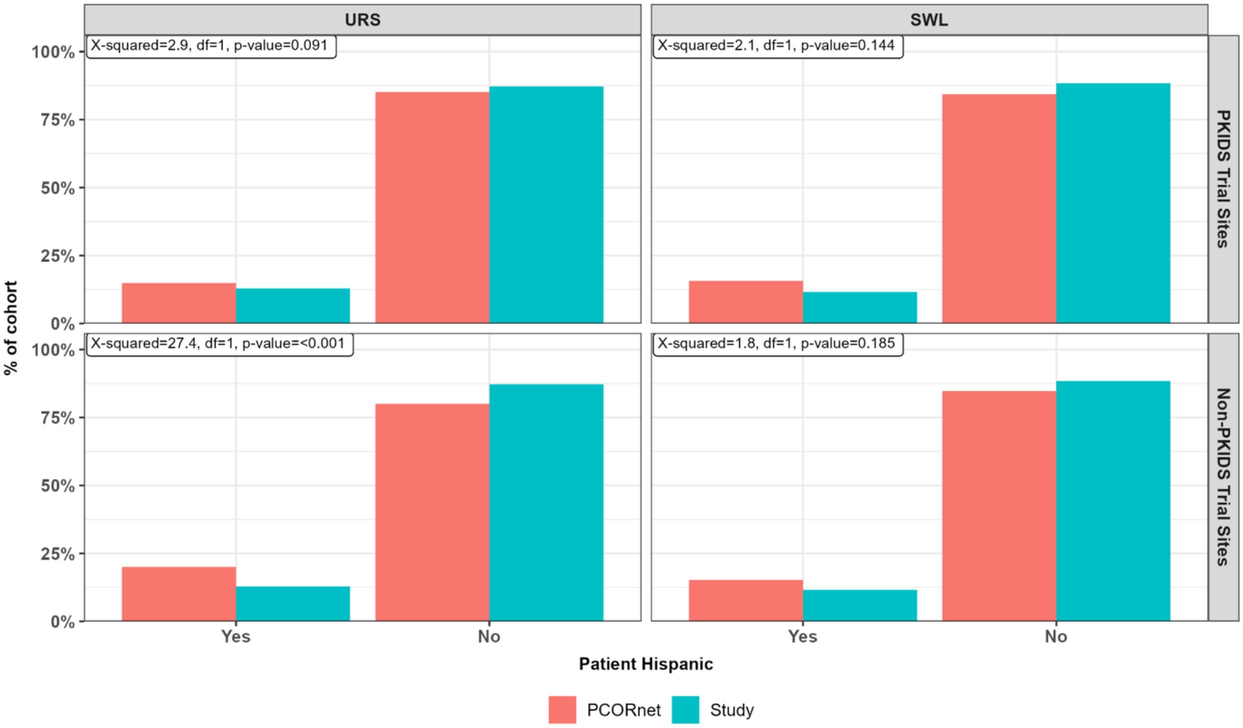

Acronyms: URS, Ureteroscopy; SWL, Shockwave Lithotripsy

© 2025 Tasian GE et al. *JAMA Network Open*.

eTable 1. Variables Included in the Multiple Imputation Analyses

**variable**	**field_label**
itt_surg_proc	patient surgery procdure at each side
pat_age	patient age
pat_gender	Sex assigned at birth
pat_race	What is [patient_firstname]'s race (check all that apply)?
pat_ethnicity	If yes, please specify.
institutional_id	PKIDS Institutional ID
surgeon_id	Surgeon ID
icd_patients	Please provide the number of unique patients with kidney stones evaluated in your Division's outpatient clinic in 2018 and 2019. (ICD-10 code = N20)
patient_age	Age of patient
family_preference	Patient/family preference
Urologist_stone_size	Stone size
equipment	Availability of equipment
one_five_cm_stone	Please indicate the strength of your preference for URS versus PCNL for a 1.5 cm kidney stone.
two_cm_stone	Please indicate the strength of your preference for SWL versus PCNL for a 2 cm kidney stone.
imaging_types	Type of Imaging
number_of_stones	How many kidney stones were visualized? If more than 3 stones visualized in a kidney, report on the THREE largest stones visualized. If stones visualized in both kidneys, report on up to 3 from each kidney.
stone_location_cat	stone location
stone_size_scale	stone size
prior_gu_reconstruction	Prior GU Reconstruction
monogenic_stone_disease	Monogenic Stone Disease
struct_abnorm	Structural Renal Abnormality
primary_ind	Identification of primary clinical indication for surgery
surgery_24hrs	Was surgery performed within 24 hours of evaluation for the patient's current symptoms?
or_access_restrictions_yn	Are there current restrictions to OR access at your institution?

© 2025 Tasian GE et al. *JAMA Network Open*.

variable	field_label
prior_diagnosis	Prior to this episode, has [patient_firstname] ever been diagnosed with a kidney stone before the one for which he/she is currently being treated?
prior_surgery	Prior to this episode, has [patient_firstname] ever required surgery for a kidney stone?
ed_visit	Has [patient_firstname] been to the emergency department or urgent care for this current stone episode?
family_history	Has anyone in [patient_firstname]'s immediate biologic family (i.e. brother/sister/father/mother/half-sibling) had a kidney stone?
family_history_surgery	Has anyone in your family or close to your family undergone kidney stone surgery?
bladder_control	Does [patient_firstname] lack bladder control due to a brain, spinal cord or nerve problem?
move_assistt	Does [patient_firstname] need assistance to ambulate/move (e.g. crutches, wheelchair)?
tube_fed	Does [patient_firstname] receive feeds/nutrition through a tube (e.g. G-tube)?
seizure	Does [patient_firstname] have a seizure disorder (e.g. epilepsy)?
keto	Does [patient_firstname] follow a ketogenic diet?
urine_collection	Has [patient_firstname] ever completed a 24-hour urine collection?
ventilator	Is [patient_firstname] on a ventilator?
blood_cells	Does [patient_firstname] have problems with their blood cells, such as sickle cell disease, thalessiemia, low red blood cell counts (anemia), low platelet counts (thrombocytopenia), low white blood cell counts (neutropenia/leukopenia) or other disease that places them at increased risk for bleeding or blood clots?
muscle	Does [patient_firstname] have a problem with scoliosis, contractures of their arms or legs, decreased muscle tone, or other progressive muscle diseases?
oxygen	Does [patient_firstname] need supplemental oxygen at home?
heart_prob	Does [patient_firstname] have a congenital or acquired heart problem?
brain_tumor	Does [patient_firstname] have a brain tumor or hydrocephalus (too much fluid in the brain)?
develop_delays	Does [patient_firstname] have diagnosed developmental delays?
expulsive_med	Is [patient_firstname] currently taking any expulsive therapies (medications to help stone passage)?

© 2025 Tasian GE et al. *JAMA Network Open*.

variable	field_label
covid_vac	Have you been vaccinated for COVID-19? (if completing questionnaire on behalf of your child, please list their vaccine status)
household_type	What type of household does [patient_firstname] live in?
education	What is your (Parent/Caregiver) highest level of education?
income	What is your household's annual income?
prim_language	Is English the primary language spoken at home?
span_his_lat	Is [patient_firstname] of Spanish/Hispanic/Latino/a origin?
food_insecurity	Within the past 12 months you (Parent/Caregiver) worried whether your food would run out before you got money to buy more.
food_amount	Within the past 12 months the food you (Parent/Caregiver) bought just didn't last and you didn't have money to get more.
insurance_type	What is [patient_firstname]'s insurance type (please select all that apply)?
keto_history	If no; Have they ever?
prior_experience_surgery	What was your experience with prior surgery?

© 2025 Tasian GE et al. *JAMA Network Open*.

eTable 2. Characteristics of Urologists Practicing in the PKIDS Trial

The urologist column reports the frequency and proportion of these characteristics among the urologists who performed surgery in the PKIDS trial. The patient column reports the frequency and proportion of those characteristics for the patients who participated in the PKIDS trial.

**Urologist Characteristics**		**Urologist (n=124)**	**Urologist Characteristics by Patient (n=1142)**
Years in practice following training, median (IQR)		6.0 (2.0, 13.5)	6.0 (3.0, 15.0)
Years in practice following training, No. (%)	0-4	52 (41.9)	398 (34.9)
	5-9	27 (21.8)	344 (30.1)
	10-19	27 (21.8)	220 (19.3)
	>=20	17 (13.7)	174 (15.2)
	Missing/ Unknown	1 (0.8)	6 (0.5)
Current age, median (IQR)		41.00 (37.0, 49.0)	41.0 (38.0, 49.0)
Current age, No. (%)	30-39	55 (44.4)	415 (36.3)
	40-49	40 (32.3)	440 (38.5)
	50-70	28 (22.6)	281 (24.6)
	Missing/ Unknown	1 (0.8)	6 (0.5)
Gender, No. (%)	Female	38 (30.6)	210 (18.4)
	Male	85 (68.5)	926 (81.1)
	Transgender Male	0 (0)	0 (0)
	Transgender Female	0 (0)	0 (0)
	Non-Binary	0 (0)	0 (0)
	Gender-Queer	0 (0)	0 (0)
	Other	0 (0)	0 (0)
	Missing/ Unknown	1 (0.8)	6 (0.5)
Urologists who completed a fellowship in Pediatric Urology, No. (%)		113 (91.1)	1117 (97.8)
Urologists who completed a fellowship in Endourology, No. (%)		8 (6.5)	41 (3.6)
Urologists follow the 2016 Guidelines for the Surgical Management of Urinary Stones in	I don't know the guidelines	0 (0)	0 (0)

© 2025 Tasian GE et al. *JAMA Network Open*.

Urologist Characteristics		Urologist (n=124)	Urologist Characteristics by Patient (n=1142)
Pediatric Patients from the American Urological Association and Endourological Society, No. (%)			
	Never	1 (0.8)	40 (3.5)
	Less than half the time	3 (2.4)	103 (9.0)
	Half the time	4 (3.2)	47 (4.1)
	More than half the time	69 (55.6)	594 (52.0)
	Always	33 (26.6)	233 (20.4)
	Missing/ Unknown	1 (0.8)	6 (0.5)
Importance placed on age of patient when choosing type of surgical treatment for stones, No. (%)	Not at all Important	0 (0)	0 (0)
	Low Importance	8 (6.5)	49 (4.3)
	Neutral	9 (7.3)	80 (7.0)
	Important	68 (54.8)	666 (58.3)
	Very Important	37 (29.8)	340 (29.8)
	Missing/ Unknown	2 (1.6)	7 (0.6)
Importance placed on sex of patient when choosing type of surgical treatment for stones, No. (%)	Not at all Important	34 (27.4)	395 (34.6)
	Low Importance	47 (37.9)	409 (35.8)
	Neutral	28 (22.6)	215 (18.8)
	Important	12 (9.7)	78 (6.8)
	Very Important	2 (1.6)	39 (3.4)
	Missing/ Unknown	1 (0.8)	6 (0.5)
Importance placed on level of patient symptoms when choosing type of surgical treatment for stones, No. (%)	Not at all Important	2 (1.6)	22 (1.9)
	Low Importance	3 (2.4)	20 (1.8)
	Neutral	4 (3.2)	51 (4.5)
	Important	54 (43.5)	581 (50.9)

© 2025 Tasian GE et al. *JAMA Network Open*.

Urologist Characteristics		Urologist (n=124)	Urologist Characteristics by Patient (n=1142)
	Very Important	60 (48.4)	462 (40.5)
	Missing/ Unknown	1 (0.8)	6 (0.5)
Importance placed on history of UTI when choosing type of surgical treatment for stones, No. (%)	Not at all Important	4 (3.2)	39 (3.4)
	Low Importance	6 (4.8)	91 (8.0)
	Neutral	15 (12.1)	144 (12.6)
	Important	61 (49.2)	615 (53.9)
	Very Important	37 (29.8)	247 (21.6)
	Missing/ Unknown	1 (0.8)	6 (0.5)
Importance placed on level of patient comorbidities when choosing type of surgical treatment for stones, No. (%)	Not at all Important	0 (0)	0 (0)
	Low Importance	1 (0.8)	1 (0.1)
	Neutral	2 (1.6)	45 (3.9)
	Important	73 (58.9)	781 (68.4)
	Very Important	47 (37.9)	309 (27.1)
	Missing/ Unknown	1 (0.8)	6 (0.5)
Importance placed on prior stone procedures when choosing type of surgical treatment for stones, No. (%)	Not at all Important	0 (0)	0 (0)
	Low Importance	3 (2.4)	24 (2.1)
	Neutral	15 (12.1)	133 (11.6)
	Important	78 (62.9)	737 (64.5)
	Very Important	27 (21.8)	242 (21.2)
	Missing/ Unknown	1 (0.8)	6 (0.5)
Importance placed on patient/family preference when choosing type of surgical treatment for stones, No. (%)	Not at all Important	0 (0)	0 (0)
	Low Importance	2 (1.6)	8 (0.7)
	Neutral	20	147

© 2025 Tasian GE et al. *JAMA Network Open*.

Urologist Characteristics		Urologist (n=124)	Urologist Characteristics by Patient (n=1142)
		(16.1)	(12.9)
	Important	70 (56.5)	505 (44.2)
	Very Important	31 (25.0)	476 (41.7)
	Missing/ Unknown	1 (0.8)	6 (0.5)
Importance placed stone size when choosing type of surgical treatment for stones, No. (%)	Not at all Important	0 (0)	0 (0)
	Low Importance	0 (0)	0 (0)
	Neutral	0 (0)	0 (0)
	Important	29 (23.4)	279 (24.4)
	Very Important	94 (75.8)	857 (75.0)
	Missing/ Unknown	1 (0.8)	6 (0.5)
Importance placed on stone location when choosing type of surgical treatment for stones, No. (%)	Not at all Important	0 (0)	0 (0)
	Low Importance	0 (0)	0 (0)
	Neutral	3 (2.4)	22 (1.9)
	Important	39 (31.5)	456 (39.9)
	Very Important	81 (65.3)	658 (57.6)
	Missing/ Unknown	1 (0.8)	6 (0.5)
Importance placed on stone density when choosing type of surgical treatment for stones, No. (%)	Not at all Important	1 (0.8)	9 (0.8)
	Low Importance	10 (8.1)	197 (17.3)
	Neutral	30 (24.2)	286 (25.0)
	Important	58 (46.8)	463 (40.5)
	Very Important	24 (19.4)	181 (15.8)
	Missing/ Unknown	1 (0.8)	6 (0.5)
Importance placed on stone number when choosing type of surgical treatment for stones, No. (%)	Not at all Important	0 (0%)	0 (0%)

© 2025 Tasian GE et al. *JAMA Network Open*.

Urologist Characteristics		Urologist (n=124)	Urologist Characteristics by Patient (n=1142)
	Low Importance	1 (0.8)	7 (0.6)
	Neutral	4 (3.2)	43 (3.8)
	Important	70 (56.5)	623 (54.6)
	Very Important	48 (38.7)	463 (40.5)
	Missing/ Unknown	1 (0.8)	6 (0.5)
Importance placed on availability of equipment when choosing type of surgical treatment for stones, No. (%)	Not at all Important	2 (1.6)	16 (1.4)
	Low Importance	9 (7.3)	106 (9.3)
	Neutral	14 (11.3)	130 (11.4)
	Important	51 (41.1)	473 (41.4)
	Very Important	47 (37.9)	411 (36.0)
	Missing/ Unknown	1 (0.8)	6 (0.5)
Importance placed on level of urologist's experience/comfort with the procedure when choosing type of surgical treatment for stones, No. (%)	Not at all Important	1 (0.8)	8 (0.7)
	Low Importance	4 (3.2)	6 (0.5)
	Neutral	12 (9.7)	112 (9.8)
	Important	61 (49.2)	650 (56.9)
	Very Important	45 (36.3)	360 (31.5)
	Missing/ Unknown	1 (0.8)	6 (0.5)
Importance placed on number of anesthetics required when choosing type of surgical treatment for stones, No. (%)	Not at all Important	0 (0)	0 (0)
	Low Importance	6 (4.8)	79 (6.9)
	Neutral	17 (13.7)	163 (14.3)
	Important	66 (53.2)	525 (46.0)
	Very Important	34 (27.4)	369 (32.3)

© 2025 Tasian GE et al. *JAMA Network Open*.

Urologist Characteristics		Urologist (n=124)	Urologist Characteristics by Patient (n=1142)
	Missing/ Unknown	1 (0.8)	6 (0.5)
Importance placed on level of radiation exposure when choosing type of surgical treatment for stones, No. (%)	Not at all Important	1 (0.8)	5 (0.4)
	Low Importance	7 (5.6)	96 (8.4)
	Neutral	26 (21.0)	276 (24.2)
	Important	64 (51.6)	548 (48.0)
	Very Important	25 (20.2)	211 (18.5)
	Missing/ Unknown	1 (0.8)	6 (0.5)
Importance placed on level of post-operative discomfort when choosing type of surgical treatment for stones, No. (%)	Not at all Important	0 (0)	0 (0)
	Low Importance	3 (2.4)	7 (0.6)
	Neutral	17 (13.7)	266 (23.3)
	Important	82 (66.1)	705 (61.7)
	Very Important	21 (16.9)	158 (13.8)
	Missing/ Unknown	1 (0.8)	6 (0.5)
Importance placed on rate of complications/harms when choosing type of surgical treatment for stones, No. (%)	Not at all Important	0 (0)	0 (0)
	Low Importance	0 (0)	0 (0)
	Neutral	3 (2.4)	16 (1.4)
	Important	59 (47.6)	592 (51.8)
	Very Important	61 (49.2)	528 (46.2)
	Missing/ Unknown	1 (0.8)	6 (0.5)
Importance placed on level of reimbursement/RVU when choosing type of surgical treatment for stones, No. (%)	Not at all Important	82 (66.1)	930 (81.4)
	Low Importance	29 (23.4)	168 (14.7)
	Neutral	11 (8.9)	37 (3.2)

© 2025 Tasian GE et al. *JAMA Network Open*.

Urologist Characteristics		Urologist (n=124)	Urologist Characteristics by Patient (n=1142)
	Important	1 (0.8)	1 (0.1)
	Very Important	0 (0)	0 (0)
	Missing/ Unknown	1 (0.8)	6 (0.5)
Strength of urologist preference for URS versus SWL for a 1cm non-lower pole stone, median (IQR)		79.0 (50.0, 99.0)	74.0 (50.0, 90.0)
Strength of urologist preference for URS versus SWL for a 9mm proximal ureteral stone, median (IQR)		90.0 (70.0, 100.0)	90.0 (75.0, 100.0)
Strength of urologist preference for URS versus PCNL for a 1.5 cm kidney stone, median (IQR)		60.0 (28.8, 75.0)	55.0 (23.0, 73.0)
Strength of urologist preference for SWL versus PCNL for a 2 cm kidney stone, median (IQR)		10.0 (0.0, 25.0)	7.0 (0.0, 25.0)
Urologists co-manage kidney stone patients with an Advanced Practice Provider (i.e., Nurse Practitioner and Physician Assistant), No. (%)		44 (35.5)	514 (45.0)
Urologists who get patient referrals for kidney stone surgery from an Advanced Practice Provider without the urologist meeting the patient before surgery, No. (%)	No	79 (63.7)	622 (54.5)
	Yes	11 (8.9)	51 (4.5)
Urologists who participate in a multi-disciplinary kidney stone clinic, No. (%)		54 (43.5)	702 (61.5)
Years urologists participated in a multi-disciplinary kidney stone clinic., No. (%)	>10 years	0 (0)	0 (0)
	5-10 years	14 (11.3)	272 (23.8)
	1-5 years	24 (19.4)	266 (23.3)
	< 1 year	9 (7.3)	72 (6.3)
	Missing/ Unknown	1 (0.8)	6 (0.5)
Urologists who use nomograms or other tools that predict success of kidney stone surgery, No. (%)		12 (9.7)	88 (7.7)
Importance of nomograms or predictive tools in surgical decision making, No. (%)	Not at all important	0 (0)	0 (0)
	Low Importance	0 (0)	0 (0)
	Neutral	6 (4.8)	21 (1.8)
	Important	6 (4.8)	67 (5.9)
	Very Important	0 (0)	0 (0)

© 2025 Tasian GE et al. *JAMA Network Open*.

Urologist Characteristics		Urologist (n=124)	Urologist Characteristics by Patient (n=1142)
	Missing/ Unknown	1 (0.8)	6 (0.5)
Urologists who refer candidates for kidney stone surgery to colleagues, No. (%)		46 (37.1)	275 (24.1)
If so, for which procedures do urologists refer, No. (%)	URS	1 (0.8)	2 (0.2)
	SWL	3 (2.4)	9 (0.8)
	PCNL	20 (16.1)	132 (11.6)
	Robotic/ laparoscopic pyelolithotomy (with or without pyeloplasty)	3 (2.4)	23 (2.0)
	Missing/ Unknown	1 (0.8)	6 (0.5)
Practices of urologists for testing the urine for presence of bacteria or infection before surgery that involves instrumenting the urinary tract, No. (%)	I routinely obtain urine analysis and culture	64 (51.6)	431 (37.7)
	I selectively obtain urine analysis and culture for children felt to be higher risk for peri-operative infection	53 (42.7)	639 (56.0)
	I never obtain pre-operative culture	5 (4.0)	63 (5.5)
	Missing/ Unknown	2 (1.6)	9 (0.8)
Practices of urologists' usage of antibiotic coverage for a patient with no history of recurrent UTI and for whom a pre-op urine culture was negative or not obtained, No. (%)	Pre-operative (started at least one day before surgery)	3 (2.4)	11 (1.0)
	Single pre-procedure dose only	84 (67.7)	736 (64.4)
	Therapeutic post-procedure dose continuing =24 hours after surgery.	0 (0%)	0 (0%)
	Post-operative antibiotic	0 (0)	0 (0)

© 2025 Tasian GE et al. *JAMA Network Open*.

Urologist Characteristics		Urologist (n=124)	Urologist Characteristics by Patient (n=1142)
	prophylaxis > 24 hours if stent/nephrostomy placed		
	Missing/ Unknown	3 (2.4)	10 (0.9)
Frequency of opioid prescriptions for ureteral stent placement, No. (%)	Never	63 (50.8)	673 (58.9)
	Less than half the time	42 (33.9)	320 (28.0)
	Half the time	6 (4.8)	60 (5.3)
	More than half the time	7 (5.6)	65 (5.7)
	Always	5 (4.0)	18 (1.6)
	I don't perform	0 (0)	0 (0)
	Missing/ Unknown	1 (0.8)	6 (0.5)
Frequency of opioid prescriptions for ureteroscopy, No. (%)	Never	35 (28.2)	333 (29.2)
	Less than half the time	46 (37.1)	477 (41.8)
	Half the time	12 (9.7)	79 (6.9)
	More than half the time	19 (15.3)	179 (15.7)
	Always	11 (8.9)	68 (6.0)
Frequency of opioid prescriptions for shockwave lithotripsy, No. (%)	Never	48 (38.7)	643 (56.3)
	Less than half the time	29 (23.4)	149 (13.0)
	Half the time	3 (2.4)	39 (3.4)
	More than half the time	15 (12.1)	131 (11.5)
	Always	8 (6.5)	53 (4.6)
	I don't perform	20 (16.1)	121 (10.6)
	Missing/ Unknown	1 (0.8)	6 (0.5)

Acronyms: Interquartile Range; IQR, Ureteroscopy, URS; Shockwave Lithotripsy, SWL; Percutaneous Nephrolithotomy, PCNL; Ureteropelvic Junction, UPJ; Emergency Department, ED; Urinary Tract Infection, UTI; Relative Value Unit, RVU

© 2025 Tasian GE et al. *JAMA Network Open*.

eTable 3. Characteristics of Medical Centers Participating in the PKIDS Trial

The institution column reports the frequency and proportion of these characteristics among the medical centers who performed surgery in the PKIDS trial. The patient column reports the frequency and proportion of those characteristics for the patients who participated in the PKIDS trial.

**Institution Characteristics**		**Institution (n=31)**	**Medical Center Characteristics by Enrolled Patients (n=1228)**
Hospital setting for pediatric urology, No. (%)	Free-standing children's hospital	23 (74.2)	1116 (90.9)
	Other	2 (6.5)	17 (1.4)
	Pediatric unit within larger hospital	6 (19.4)	95 (7.7)
Number of inpatient beds in hospital, median (IQR)	Median (IQR)	312.0 (245.0, 517.5)	415.0 (298.0, 564.0)
Number of inpatient beds in hospital, No. (%)	100-149	2 (6.5)	57 (4.)
	250-349	16 (51.6)	477 (38.8)
	350-549	5 (16.1)	197 (16.0)
	550-649	3 (9.7)	206 (16.8)
	>=650	5 (16.1)	291 (23.7)
Number of unique patients with kidney stones evaluated in division's outpatient clinic in 2018 and 2019. (ICD-10 code = N20), median (IQR)	Median (IQR)	222.0 (122.0, 414.5)	260.0 (202.0, 626.0)
Number of unique patients with kidney stones evaluated in division's outpatient clinic in 2018 and 2019. (ICD-10 code = N20), no. (%)	<=149	10 (32.3)	237 (19.3)
	150-299	9 (29.0)	393 (32.0)
	350-599	0 (0)	0 (0)
	>=600	5 (16.1)	444 (36.2)
Number of unique patients with kidney stones admitted to the hospital in 2018 and 2019, median (IQR)	Median (IQR)	45.0 (16.5, 124.5)	47.0 (30.0, 165.0)

© 2025 Tasian GE et al. *JAMA Network Open*.

Institution Characteristics		Institution (n=31)	Medical Center Characteristics by Enrolled Patients (n=1228)
Number of unique patients with kidney stones admitted to the hospital in 2018 and 2019, No. (%)	<=24	9 (29.0)	203 (16.5)
	25-49	8 (25.8)	444 (36.2)
	50-149	6 (19.4)	178 (14.5)
	>=150	8 (25.8)	403 (32.8)
Number of unique patients with kidney stones seen in your hospital's ED in 2018 and 2019, median (IQR)	Median (IQR)	94.0 (52.0, 166.0)	111.0 (74.0, 199.0)
Number of unique patients with kidney stones seen in hospital's ED in 2018 and 2019, No. (%)	<=49	6 (19.4)	155 (12.6)
	50-99	11 (35.5)	445 (36.2)
	100-199	9 (29.0)	469 (38.2)
	>=200	5 (16.1)	159 (12.9)
Payor for the majority of patients with kidney stones, No. (%)	Commercial insurer	15 (48.4)	859 (70.0)
	Medicaid	15 (48.4)	362 (29.5)
	Uninsured	1 (3.2)	7 (0.6)
	Missing/Unknown	0 (0)	0 (0)
Have a pediatric urology fellowship program, No. (%)		19 (61.3)	986 (80.3)
Type of facility are pediatric kidney stone cases performed, No. (%)	Children's hospital	21 (67.7)	1020 (83.1)
	Children's hospital Adult hospital	5 (16.1)	91 (7.4)
	Children's hospital Adult hospital Surgery Center	3 (9.7)	50 (4.1)
	Children's hospital Surgery Center	2 (6.)	67 (5.5)

© 2025 Tasian GE et al. *JAMA Network Open*.

Institution Characteristics		Institution (n=31)	Medical Center Characteristics by Enrolled Patients (n=1228)
Type of imaging is used routinely (>75%) at your institution for PCNL access, No. (%)	Fluoroscopy	16 (51.6)	431 (35.1)
	Fluoroscopy Ultrasound	8 (25.8)	310 (25.2)
	Fluoroscopy Ultrasound CT	4 (12.9)	127 (10.3)
	Ultrasound	3 (9.7)	360 (29.3)
Statement that best characterizes laser access at hospital, No. (%)	My hospital owns a holmium laser	23 (74.2)	1017 (82.8)
	My hospital rents a holmium laser.	8 (25.8)	211 (17.2)
Institution provides off-hours access (after 5:00pm on weekday and weekend) to a holmium laser, No. (%)		26 (83.9)	1153 (93.9)
Provides technician/resource personnel (e.g. "laser nurse") for cases that use a holmium laser, No. (%)		21 (67.7)	1012 (82.4)
Statement that best characterizes SWL access at hospital, No. (%)	My hospital owns a shockwave lithotripter.	3 (9.7)	61 (5.0)
	My hospital rents a shockwave lithotripter	2 (90.3)	1167 (95.0)
Provides off-hours access (after 5:00pm on weekday and weekend) to a shockwave lithotripter, No. (%)		3 (9.7)	90 (7.3)
Interventional Radiology available 24 hours a day, 7-days a week at hospital, No. (%)		31 (100)	1228 (100)
Have a multi-disciplinary kidney stone clinic, No. (%)		19 (61.3)	886 (72.1)

Acronyms: Ureteroscopy, URS; Shockwave Lithotripsy, SWL

© 2025 Tasian GE et al. *JAMA Network Open*.

eTable 4. Standardized Mean Differences of Patient, Surgeon, and Health System Characteristics Between Ureteroscopy and Shockwave Lithotripsy Treatment Groups Before and After Propensity Score Weighting

					Standardized Mean Difference	
**Characteristic**		**URS (n=1070)**	**SWL (n=197)**	**P-value***	**Pre-weighted SMD**	**Weighted SMD**
Patient age group, No. (%)	08-11	216 (20.2%)	45 (22.8%)	0.246	-0.06	-0.04
	12-15	377 (35.2%)	60 (30.5%)		0.10	0.03
	16-18	376 (35.1%)	66 (33.5%)		0.03	0.01
	19-21	101 (9.4%)	26 (13.2%)		-0.12	-0.01
Prior genitourinary reconstruction, No. (%)	Yes	54 (5.0%)	11 (5.6%)	0.380	-0.02	-0.02
	Missing/ Unknown	10 (0.9%)	0 (0%)		0.14	0.01
Monogenic Stone Disease, No. (%)	Yes	34 (3.2%)	1 (0.5%)	0.042	0.20	0.08
Patient experience with prior surgery, No. (%)	Positive	134 (12.5%)	20 (10.2%)	0.011	0.07	0.03
	Negative	17 (1.6%)	11 (5.6%)		-0.22	-0.05
	Neutral	41 (3.8%)	6 (3.0%)		0.04	-0.01
	No prior surgery	784 (73.3%)	142 (72.1%)		0.03	0.01
	Missing/ Unknown	94 (8.8%)	18 (9.1%)		-0.01	0.00
Patient has problems with their blood cells, No. (%)	Yes	47 (4.4%)	8 (4.1%)	0.976	0.02	0.01
Patient has scoliosis, contractures of their arms or legs, decreased muscle tone, or other progressive muscle diseases, No. (%)	Yes	141 (13.2%)	33 (16.8%)	0.400	-0.10	-0.01
Structural renal abnormality, No. (%)	Yes	122 (11.4%)	10 (5.1%)	0.011	0.23	0.01
Identification of primary clinical indication for surgery, No. (%)	Pain	702 (65.6%)	75 (38.1%)	<0.001	0.57	0.04

© 2025 Tasian GE et al. *JAMA Network Open*.

					Standardized Mean Difference	
**Characteristic**		**URS (n=1070)**	**SWL (n=197)**	**P-value***	**Pre-weighted SMD**	**Weighted SMD**
	Infection	88 (8.2%)	4 (2.0%)		0.28	0.12
	Hematuria	13 (1.2%)	3 (1.5%)		-0.03	-0.02
	Stone Growth	106 (9.9%)	43 (21.8%)		-0.33	-0.04
	Elective	111 (10.4%)	69 (35.0%)		-0.62	-0.05
	Other	50 (4.7%)	3 (1.5%)		0.18	0.04
Surgery performed within 24 hours of evaluation for the patient's current symptoms, No. (%)	Yes	111 (10.4%)	5 (2.5%)	0.002	0.32	0.02
Current restrictions to OR access at institution, No. (%)	Yes	10 (0.9%)	2 (1.0%)	0.398	-0.01	-0.03
Type of pre-op imaging, No. (%)	CT	444 (41.5%)	20 (10.2%)	<0.001	0.77	0.08
	US	590 (55.1%)	168 (85.3%)		-0.70	-0.05
	KUB	14 (1.3%)	8 (4.1%)		-0.17	-0.07
	Other	6 (0.6%)	0 (0%)		0.11	0.09
	No preop imaging data	16 (1.5%)	1 (0.5%)		0.10	0.01
Number of kidney stones visualized on the procedure side, No. (%)	No stone present	63 (5.9%)	2 (1.0%)	0.009	0.27	0.03
	1	624 (58.3%)	130 (66.0%)		-0.16	-0.03
	2	223 (20.8%)	49 (24.9%)		-0.10	0.00
	3	101 (9.4%)	14 (7.1%)		0.08	0.03
	4	33 (3.1%)	1 (0.5%)		0.19	0.05
	5	5 (0.5%)	0 (0%)		0.10	0.00

© 2025 Tasian GE et al. *JAMA Network Open*.

					Standardized Mean Difference	
**Characteristic**		**URS (n=1070)**	**SWL (n=197)**	**P-value***	**Pre-weighted SMD**	**Weighted SMD**
	6	3 (0.3%)	0 (0%)		0.07	0.01
	Missing/ Unknown	18 (1.7%)	1 (0.5%)		0.11	0.01
Location:	Lower pole kidney	198 (18.5%)	68 (34.5%)	<0.001	-0.37	-0.05
	Non-lower pole kidney	311 (29.1%)	98 (49.7%)		-0.43	-0.04
	Ureter (includes UPJ)	480 (44.9%)	28 (14.2%)		0.71	0.12
	No stone present	63 (5.9%)	2 (1.0%)		-0.27	-0.02
	Missing/ Unknown	18 (1.7%)	1 (0.5%)		-0.11	0.00
Maximum of Longest Dimension, No. (%)	<7	505 (47.2%)	70 (35.5%)	<0.001	0.24	0.02
	7-10	274 (25.6%)	91 (46.2%)		-0.44	-0.03
	10-15	113 (10.6%)	24 (12.2%)		-0.05	-0.01
	>15	50 (4.7%)	5 (2.5%)		0.11	0.00
	Missing size	47 (4.4%)	4 (2.0%)		0.13	0.02
	No stone present	63 (5.9%)	2 (1.0%)		-0.27	-0.02
	Missing/ Unknown	18 (1.7%)	1 (0.5%)		-0.11	0.00
Number of unique patients with kidney stones evaluated in Urology outpatient clinic in 2018 and 2019 (ICD-10 code = N20), No. (%)	0-149	214 (20.0%)	28 (14.2%)	<0.001	0.15	0.01
	150-299	401 (37.5%)	17 (8.6%)		0.73	0.13
	300-599	141 (13.2%)	17 (8.6%)		0.15	0.00
	>=600	314 1 (29.3%)	135 (68.5%)		-0.85	-0.09

© 2025 Tasian GE et al. *JAMA Network Open*.

					Standardized Mean Difference	
**Characteristic**		**URS (n=1070)**	**SWL (n=197)**	**P-value***	**Pre-weighted SMD**	**Weighted SMD**
Importance that urologist places on age of patient when choosing type of surgical treatment for stones, No. (%)	Low Importance	36 (3.4%)	17 (8.6%)	<0.001	-0.22	-0.03
	Neutral	80 (7.5%)	8 (4.1%)		0.15	-0.03
	Important	614 (57.4%)	126 (64.0%)		-0.13	0.01
	Very Important	331 (30.9%)	46 (23.4%)		0.17	0.01
	Missing/ Unknown	9 (0.8%)	0 (0%)		0.13	0.00
Importance that urologist places on patient/family preference when choosing type of surgical treatment for stones, No. (%)	Low Importance	8 (0.7%)	1 (0.5%)	<0.001	0.03	-0.02
	Neutral	153 (14.3%)	9 (4.6%)		0.34	0.03
	Important	488 (45.6%)	66 (33.5%)		0.25	0.03
	Very Important	413 (38.6%)	121 (61.4%)		-0.47	-0.04
	Missing/ Unknown	8 (0.7%)	0 (0%)		0.12	0.00
Importance that urologist places on stone size when choosing type of surgical treatment for stones, No. (%)	Important	282 (26.4%)	28 (14.2%)	<0.001	0.31	0.02
	Very Important	780 (72.9%)	169 (85.8%)		-0.32	-0.02
	Missing/ Unknown	8 (0.7%)	0 (0%)		-0.12	-0.01
Importance that urologist places on availability of equipment when choosing type of surgical treatment for stones, No. (%)	Not at all Important	20 (1.9%)	1 (0.5%)	0.189	0.13	0.04
	Low Importance	99 (9.3%)	19 (9.6%)		-0.01	-0.01

© 2025 Tasian GE et al. *JAMA Network Open*.

					Standardized Mean Difference	
**Characteristic**		**URS (n=1070)**	**SWL (n=197)**	**P-value***	**Pre-weighted SMD**	**Weighted SMD**
	Neutral	119 (11.1%)	28 (14.2%)		-0.09	-0.01
	Important	442 (41.3%)	69 (35.0%)		0.13	-0.01
	Very Important	382 (35.7%)	80 (40.6%)		-0.10	0.01
	Missing/ Unknown	8 (0.7%)	0 (0%)		-0.12	-0.01
Strength of urologist preferences for URS versus PCNL for a 1.5 cm kidney stone, No. (%)	0-29	307 (28.7%)	98 (49.7%)	<0.001	-0.44	-0.01
	30-49	81 (7.6%)	25 (12.7%)		-0.17	-0.04
	50-69	279 (26.1%)	33 (16.8%)		0.23	0.00
	70-79	246 (23.0%)	31 (15.7%)		0.18	0.04
	80-100	119 (11.1%)	10 (5.1%)		0.22	-0.02
	Missing/ Unknown	38 (3.6%)	0 (0%)		0.27	0.05
Strength of urologist preferences for SWL versus PCNL for a 2 cm kidney stone, No. (%)	0	373 (34.9%)	80 (40.6%)	<0.001	-0.12	0.04
	1-9	133 (12.4%)	52 (26.4%)		-0.36	-0.03
	10-29	327 (30.6%)	36 (18.3%)		0.29	0.02
	30-49	124 (11.6%)	6 (3.0%)		0.33	-0.02
	50-100	92 (8.6%)	22 (11.2%)		-0.09	-0.04
	Missing/ Unknown	21 (2.0%)	1 (0.5%)		0.13	0.04

Acronyms: Ureteroscopy, URS; Shockwave Lithotripsy, SWL; Body Mass Index; Ureteropelvic Junction, UPJ; Emergency Department, ED; Central Nervous System, CNS; Urinary Tract Infection, UTI

© 2025 Tasian GE et al. *JAMA Network Open*.

eTable 5. Proportion of Participants Undergoing Ureteroscopy and Shockwave Lithotripsy Whose Difference in Score at 1 Week Exceeded 30% and 50% of the SD of the Patient-Reported Outcome Measure Among All Participants at Baseline

Patient Reported Outcome Instrument	Standard deviation of weighted score of study sample at baseline	The proportion of participants whose difference in the score at 1 week exceeded 30% baseline standard deviation, % (95% CI)			The proportion of participants whose difference in the score at 1 week exceeded 50% baseline standard deviation, % (95% CI)		
		URS	SWL	Difference	URS	SWL	Difference
PROMIS Pain Intensity	10.05	48.9 (36.9,60.9)	41.8 (33, 50.6)	7.1 (-7.7, 22)	43 (30.9,55.1)	35.2 (26.8, 43.7)	7.7 (-7, 22.5)
PROMIS Pain Interference	13.36	50 (38.5,61.5)	41.8 (33.4, 50.1)	8.2 (-6, 22.4)	46.3 (34.7, 57.9)	35.1 (27, 43.2)	11.3 (-2.9, 25.4)
PROMIS Anxiety	12.48	34.5 (22.9,46.1)	32.6 (24.5, 40.6)	2 (-12.2, 16.1)	29.7 (18.3, 41.2)	25.6 (18, 33.1)	4.1 (-9.6, 17.9)
PROMIS Peer Relationships	8.55	30.5 (20.2,40.8)	37.4 (28.8, 46)	-6.9 (-20.3, 6.5)	25.6 (15.8, 35.5)	27.6 (19.7, 35.6)	-2 (-14.7, 10.7)
PROMIS Sleep Disturbances	11.18	32.5 (21.9,43.1)	33.1 (24.8, 41.4)	-0.6 (-14, 12.9)	24 (15.6, 32.4)	24.2 (16.7, 31.7)	-0.2 (-11.5, 11.1)
PROMIS Stress Experiences	11.19	25.9 (15.6,36.1)	24.2 (16.8, 31.6)	1.7 (-11, 14.3)	19.1 (10.8, 27.4)	15.6 (9.3, 21.9)	3.5 (-7, 13.9)
DVSS score† (urinary symptoms)	4.07	41.2 (28.8,53.6)	24 (16.4, 31.6)	17.2 (2.7, 31.8)	30.3 (17.9, 42.7)	16.5 (9.9, 23.2)	13.8 (-0.3, 27.8)
QUIKSS‡ score (urinary symptoms)	10.90	53.4 (41.4,65.4)	38.3 (29.6,47)	15 (0.2, 29.8)	48.7 (36.5, 60.9)	27.5 (19.5, 35.5)	21.2 (6.6, 35.8)

† The range of the Dysfunctional Voiding Symptom Score (DVSS) is 0 to 30.
‡ The range of the Questionnaire for Urinary Issues – Kidney Stone Surgery (QUIKSS) is 0 to 56. QUIKSS is comprised of 16 items that used a frequency response scale and a 7-day recall period. Each item was scored from never (0) to always (4). The total score was the sum of the items.
Results reflect analysis of imputed data. Variables used to impute missing data included all baseline covariates, treatment variable, and observed outcomes that were used in the analysis. Twenty imputed datasets were created and analyzed separately, and the results were combined using Rubin's rules to produce valid statistical inferences.

© 2025 Tasian GE et al. *JAMA Network Open*.

eTable 6. Frequency of Unanticipated Postoperative Healthcare Encounters After Ureteroscopy and Shockwave Lithotripsy

**Unanticipated post-operative healthcare encounters**	**Weighted**		
	**Ureteroscopy, % (95% CI)**	**Shockwave Lithotripsy, % (95% CI)**	**Risk Difference between Ureteroscopy and Shockwave Lithotripsy**
Unexpected acute health visits, % (95% CI)	17.3 (10.1, 24.6)	10.1 (5.8, 14.3)	7.3 (-1.2, 15.7)
Emergency department and urgent care visits, % (95% CI)	13.7 (7.3, 20.1)	9 (4.9, 13.1)	4.7 (-2.9, 12.3)
Inpatient admissions, % (95% CI)	8.6 (2.7,14.5)	4.2 (1.4,7.1)	4.4 (-2.2, 10.9)
Other acute visits, % (95% CI)	0.3 (-0.2, 0.7)	0 (0, 0)	0.3 (-0.2, 0.7)
Unanticipated procedure, % (95% CI)	3.2 (0.8, 5.7)	2.1 (0.1,4.2)	1.1 (-2.1 ,4.3)

Results reflect analysis of imputed data. Variables used to impute missing data included all baseline covariates, treatment variable, and observed outcomes that were used in the analysis. Twenty imputed datasets were created and analyzed separately, and the results were combined using Rubin's rules to produce valid statistical inferences.

© 2025 Tasian GE et al. *JAMA Network Open*.

eTable 7. Stone Clearance 4 to 8 Weeks After Ureteroscopy and Shockwave Lithotripsy, Overall and by Stone Size and Stone Location

Results reflect analysis of complete data.

		Weighted		
		**Ureteroscopy (95% CI)**	**Shockwave Lithotripsy (95% CI)**	**Ureteroscopy - Shockwave Lithotripsy (95% CI)**
Stone Clearance, % (95% CI)		74.5 (65.7, 83.3)	68.6 (59.7, 77.5)	5.9 (-6.6, 18.5)
Stone size				
	<7mm	77.3 (63.3, 91.3)	80.6 (66.7, 94.6)	-3.3 (-23.1, 16.4)
	7-10mm	71.6 (56.3, 86.9)	69.6 (56.2 ,82.9)	2 (-18.3, 22.3)
	10-15mm	76.4 (56, 96.8)	41.7 (13.5, 69.8)	34.7 (0, 69.4)
	>15mm	51.2 (-4.2, 106.7)	66.7 (12.1, 121.2)	-15.4 (-93.2, 62.3)
Stone location				
	Lower pole kidney	64.7 (47.7, 81.8)	69.2 (51.4, 87)	-4.5 (-29.2, 20.2)
	Non-lower pole kidney	74 (60.5, 87.4)	62.3 (49.2, 75.4)	11.7 (-7, 30.5)
	Ureter (includes UPJ)	85.6 (66.7, 104.6)	100 (100, 100)	-14.4 (-33.3, 4.6)

Acronyms: Confidence Interval, CI; Ureteropelvic Junction, UPJ

© 2025 Tasian GE et al. *JAMA Network Open*.

eTable 8. Complete Data Analysis of the Impact of Ureteroscopy Compared to Shockwave Lithotripsy on Patient-Reported Outcomes of Physical, Emotional, and Social Health at 1 Week After Surgery

Estimates with 95% confidence intervals not covering zero are bolded.

	**Week 1**		**Week 3**		**Week 6**		**Month 3**	
**PRO**	**Baseline (pre-op) effect**	**Procedure effect**	**Baseline (pre-op) effect**	**Procedure effect**	**Baseline (pre-op) effect**	**Procedure effect**	**Baseline (pre-op) effect**	**Procedure effect**
DVSS score (urinary symptoms)	**0.6 (0.4, 0.7)**	**1.0 (0.1, 1.9)**	**0.6 (0.5, 0.7)**	-0.1 (-0.9, 0.8)	**0.5 (0.3, 0.6)**	-0.7 (-1.6, 0.3)	**0.5 (0.3, 0.6)**	-0.2 (-1.1, 0.7)
QUIKSS score (urinary symptoms)	**0.5 (0.4, 0.7)**	**5.9 (3.3, 8.4)**	**0.3 (0.1, 0.4)**	1.0 (-1.7, 3.8)	**0.2 (0.1, 0.3)**	-2.7 (-4.7, - 0.8)	**0.2 (0.1, 0.4)**	-0.5 (-2.2, 1.2)
PROMIS Anxiety	**0.7 (0.6, 0.9)**	2.7 (-0.1, 5.5)	**0.6 (0.5, 0.8)**	2.4 (-0.1, 4.8)	**0.6 (0.4, 0.8)**	-0.3 (-2.9, 2.3)	**0.4 (0.2, 0.6)**	-2.4 (-5.0, 0.3)
PROMIS Pain Intensity	**0.3 (0.1, 0.6)**	**4.8 (1.6, 7.9)**	**0.2 (0.1, 0.4)**	0.1 (-2.5, 2.6)	**0.2 (-0.1, 0.4)**	0.3 (-2.7, 3.3)	**0.1 (0.0, 0.3)**	-0.6 (-2.8, 1.6)
PROMIS Pain Interference	**0.3 (0.2, 0.5)**	**8.1 (4.7, 11.5)**	**0.2 (0.1, 0.4)**	2.7 (-0.6, 5.9)	**0.2 (0.0, 0.4)**	0.1 (-3.4, 3.5)	**0.2 (0.1, 0.3)**	-1.6 (-3.9, 0.7)
PROMIS Peer Relationships	**0.7 (0.5, 0.9)**	-1.22 (-3.6, 1.2)	**6. 0.7 (0.5, 0.9)**	-2.0 (-4.5, 0.5)	**0.8 (0.6, 0.9)**	-1.4 (-3.9, 1.1)	**0.6 (0.5, 0.8)**	1.0 (-1.7, 3.6)
PROMIS Sleep Disturbances	**0.7 (0.5, 0.8)**	1.37 (-1,3.73)	**0.6 (0.5, 0.8)**	-0.5 (-2.6, 1.7)	**0.7 (0.5, 0.8)**	-1.4 (-3.7, 0.8)	**0.6 (0.4, 0.7)**	-3.0 (-5.6, -0.5)

© 2025 Tasian GE et al. *JAMA Network Open*.

	Week 1		Week 3		Week 6		Month 3	
**PRO**	**Baseline (pre-op) effect**	**Procedure effect**	**Baseline (pre-op) effect**	**Procedure effect**	**Baseline (pre-op) effect**	**Procedure effect**	**Baseline (pre-op) effect**	**Procedure effect**
PROMIS Stress Experiences	**0.7 (0.6, 0.8)**	1.42 (-0.6, 3.4)	**0.8 (0.6, 0.9)**	-1.5 (-4.0, 0.9)	**0.7 (0.5, 0.8)**	-1.1 (-3.6, 1.4)	**0.5 (0.4, 0.7)**	-2.7 (-5.6, 0.2)

© 2025 Tasian GE et al. *JAMA Network Open*.

eTable 9. Stone Clearance Up to 16 Weeks After Ureteroscopy and Shockwave Lithotripsy, Overall and by Stone Size and Stone Location

Results reflect analysis of imputed data.

		Weighted		
		**Ureteroscopy (95% CI)**	**Shockwave Lithotripsy (95% CI)**	**Ureteroscopy - Shockwave Lithotripsy (95% CI)**
Stone Clearance, % (95% CI)		69.8 (61.3, 78.3)	68.7 (61.2, 76.2)	1.1 (-10.2, 12.4)
Stone size				
	<7mm	74.7 (60.6, 88.7)	78.3 (66.3, 90.2)	-3.6 (-22, 14.9)
	7-10mm	67.2 (53.3, 81.2)	71 (59.6, 82.3)	-3.7 (-21.7,14.2)
	10-15mm	68.4 (46.6, 90.2)	50 (26.8, 73.2)	18.4 (-13.4,50.3)
Stone location				
	Lower pole kidney	59.2 (41.9, 76.4)	65 (50.2,79.8)	-5.8 (-28.6, 16.9)
	Non-lower pole kidney	66.1 (53.2, 79)	66.2 (54.9, 77.4)	0 (-17.2, 17.1)
	Ureter (includes UPJ)	88.2 (74.9, 101.5)	95.8 (87.8, 103.8)	-7.7 (-23.2, 7.9)

Acronyms: Ureteropelvic Junction, UPJ

© 2025 Tasian GE et al. *JAMA Network Open*.

eTable 10. Results of Sensitivity Analyses With Complete Data and Replacing Missing Data for Stone Clearance With Fixed Values

	**Weighted Stone Clearance (%)**		
**Scenario**	**Ureteroscopy (95% CI)**	**Shockwave Lithotripsy (95% CI)**	**Ureteroscopy - Shockwave Lithotripsy (95% CI)**
Estimates from complete data analysis	74.5 (65.7, 83.3)	68.6 (59.7, 77.5)	5.9 (-6.6, 18.5)
All cleared	86 (81.1, 91)	83.2 (78, 88.5)	2.8 (-4.4, 10)
All not cleared	40.8 (32.4, 49.3)	36.5 (29.8, 43.3)	4.3 (-6.5, 15.1)

Acronyms: Confidence Interval, CI

© 2025 Tasian GE et al. *JAMA Network Open*.

eTable 11. Stone Clearance Excluding Patients Aged 19 to 21 Years

		Weighted Ureteroscopy vs Shockwave Lithotripsy		
		**Ureteroscopy N=440**	**Shockwave Lithotripsy N=86**	**Ureteroscopy - Shockwave Lithotripsy**
Stone clearance, % (95% CI)		74.3 (64.9, 83.7)	70.9 (61.3, 80.6)	3.4 (-10.1,16.8)
Stone size				
	<7mm	77.4 (62.7, 92.2)	84 (69.6, 98.4)	-6.6 (-27.2,14.1)
	7-10mm	69.5 (52.8, 86.3)	66.7 (51.8, 81.5)	2.9 (-19.5, 25.3)
	10-15mm	79.4 (59.3, 99.5)	45.5 (15.8, 75.1)	34 (-1.9, 69.8)
Stone location				
	Lower pole kidney	62.8 (44.1, 81.6)	68.2 (48.6, 87.7)	-5.3 (-32.4, 21.8)
	Non-lower pole kidney	74.6 (60.7, 88.4)	62.8 (48.3, 77.3)	11.8 (-8.3, 31.8)
	Ureter (includes ureteropelvic junction)	83.7 (62.4, 100)	100 (100, 100)	-16.3 (-37.6, 4.9)

Acronyms: Confidence Interval, CI

© 2025 Tasian GE et al. *JAMA Network Open*.

eTable 12. Stone Clearance Excluding Patients With Renal Anomalies

		**Weighted Ureteroscopy vs Shockwave Lithotripsy**		
		**Ureteroscopy N=431**	**Shockwave Lithotripsy N=102**	**Ureteroscopy - Shockwave Lithotripsy**
Stone clearance, % (95% CI)		74.1 (64.9, 83.3)	68.6 (59.6, 77.6)	5.4 (-7.4, 18.3)
Stone size				
	<7mm	79.2 (65.3, 93.2)	80 (65.7, 94.3)	-0.8 (-20.8, 19.2)
	7-10mm	70.5 (54.6, 86.4)	68.9 (55.3, 82.5)	1.6 (-19.3, 22.5)
	10-15mm	75.8 (54.9, 96.8)	41.7 (13.5, 69.8)	34.2 (-0.9, 69.3)
Stone location				
	Lower pole kidney	62.2 (43.9, 80.4)	68 (49.6, 86.4)	-5.8 (-31.7, 20.1)
	Non-lower pole kidney	75.4 (62.1, 88.7)	61.5 (48.3, 74.8)	13.9 (-4.9, 32.7)
	Ureter (includes ureteropelvic junction)	85.5 (66.3, 100)	100 (100, 100)	-14.5 (-33.7, 4.7)

Acronyms: Confidence Interval, CI
Renal anomalies: Horseshoe Kidney, Malrotation, Pelvic Kidney, Chronic Hydronephrosis, Cross-Fused Extopia, Complete Duplication, Partial Duplication, Transplant Kidney, Calyceal Diverticulum

© 2025 Tasian GE et al. *JAMA Network Open*.

eTable 13. Stone Clearance for Ureteroscopy and Shockwave Lithotripsy at 4 to 8 Weeks After Surgery, Overall and by Stone Size and Stone Location, Incorporating the Sensitivity and Specificity of Local Ultrasound Interpretations Determined by Central Review of a 10% Sample

		**Weighted Ureteroscopy vs Shockwave Lithotripsy**		
		**Ureteroscopy N=474**	**Shockwave Lithotripsy N=105**	**Ureteroscopy - Shockwave Lithotripsy**
Stone clearance, % (95% CI)		71.7 (62.2, 81.1)	65.3 (56, 74.7)	6.3 (-7, 19.6)
Stone size				
	<7mm	74.7 (59.6, 89.8)	78.3 (63.1, 93.5)	-3.6 (-25, 17.8)
	7-10mm	68.5 (52.3, 84.7)	66.4 (52.3, 80.4)	2.1 (-19.3, 23.6)
	10-15mm	73.6 (51.7, 95.6)	38.2 (10.9, 65.5)	35.5 (0.5, 70.5)
Stone location				
	Lower pole kidney	61.3 (43.6, 79)	66 (47.3, 84.8)	-4.7 (-30.5, 21.1)
	Non-lower pole kidney	71.1 (56.7, 85.4)	58.8 (45.3, 72.3)	12.3 (-7.4, 32)
	Ureter (includes ureteropelvic junction)	83.8 (62.8, 100)	100 (100, 100)	-16.2 (-37.2, 4.7)

Acronyms: Confidence Interval, CI

© 2025 Tasian GE et al. *JAMA Network Open*.

eTable 14. Results of Analyses Replacing Missing Stone Clearance With a Range of Values That Are Nondifferential Across Ureteroscopy and Shockwave Lithotripsy

Each row is the percent change in stone clearance from the primary analysis for both ureteroscopy and shockwave lithotripsy.

Assumed stone clearance for missing data	Weighted Stone Clearance (%)		
	Ureteroscopy (95% CI)	Shockwave Lithotripsy (95% CI)	Ureteroscopy - Shockwave Lithotripsy (95% CI)
-0.25	62.6 (57.4, 67.7)	56.9 (51.8, 61.4)	5.8 (-1.3, 12.6)
-0.2	65 (59.3, 70.1)	59.3 (54.3, 64)	5.7 (-1.5, 13.2)
-0.15	67.3 (61.6, 72.6)	61.6 (56.3, 66.5)	5.7 (-2, 12.9)
-0.1	69.5 (64, 74.8)	64 (59.4, 68.5)	5.5 (-1.6, 12.4)
-0.05	71.7 (66.6, 76.4)	66.2 (61.9, 70.6)	5.5 (-1.6, 12)
0	74.1 (69.2, 78.5)	68.5 (64, 72.6)	5.6 (-0.4, 11.9)
0.05	76.3 (71.8, 80.4)	70.9 (66.5, 75.1)	5.3 (-1.1, 11.7)
0.1	78.6 (74.5, 82.1)	73.2 (69, 77.2)	5.5 (-0.4, 11.3)
0.15	80.9 (77, 83.8)	75.6 (72.1, 78.7)	5.3 (-0.2, 10)
0.2	83.1 (79.9, 85.1)	78 (75.1, 80.7)	5.1 (1.1, 8.9)
0.25	85.3 (83.4, 86)	80.2 (77.7, 82.2)	5.1 (2.3, 7.8)

Acronyms: Confidence Interval, CI

eTable 15. Results of Analyses Replacing Missing Stone Clearance With a Range of Values That Are Differential Across Ureteroscopy and Shockwave Lithotripsy

Assumed stone clearance for missing data		Weighted Stone Clearance (%)		
**Ureteroscopy**	**Shockwave Lithotripsy**	**Ureteroscopy (95% CI)**	**Shockwave Lithotripsy (95% CI)**	**Ureteroscopy - Shockwave Lithotripsy (95% CI)**
1	0	86 (81.1, 91)	36.5 (29.8, 43.3)	49.5 (41.2, 57.8)
0.9	0.1	81.5 (77.6, 84.2)	41.2 (38.6, 44.2)	40.3 (35.4, 44.3)
0.8	0.2	77 (72.1, 81.1)	45.8 (42.1, 49.2)	31.2 (24.9, 36.6)
0.7	0.3	72.5 (67.7, 77.1)	50.5 (46.7, 54.8)	21.9 (15.9, 28.5)
0.6	0.4	67.9 (62.6, 73.1)	55.2 (51.3, 59.9)	12.8 (5.3, 19.8)
0.5	0.5	63.4 (58.1, 68.9)	59.8 (54.8, 64.5)	3.6 (-3, 10.6)
0.4	0.6	58.8 (53.8, 64)	64.7 (59.9, 69)	-5.9 (-12.4, 1.5)
0.3	0.7	54.2 (49.7, 59.6)	69.2 (64.5, 73.6)	-15 (-21, -8.3)
0.2	0.8	49.7 (46.1, 54.3)	73.9 (70.1, 77.7)	-24.2 (-29.9, -18.1)
0.1	0.9	45.3 (42.8, 48.8)	78.5 (75.6, 81.2)	-33.2 (-37, -28.5)
0	1	40.8 (32.4, 49.3)	83.2 (78, 88.5)	-42.4 (-52.3, -32.5)

Acronyms: Confidence Interval, CI

© 2025 Tasian GE et al. *JAMA Network Open*.

eTable 16. Results of Analyses Comparing Patient-Reported Outcomes Between Ureteroscopy and Shockwave Lithotripsy, Excluding Patients Who Had a Ureteral Stent Placed at Time of Index Surgery

Patient Reported Outcome Instrument	Baseline Mean* score (95% CI)		Post-operative Mean* score (95% CI)		Association of Ureteroscopy compared to Shockwave Lithotripsy, controlling for baseline
	Ureteroscopy	Shockwave lithotripsy	Ureteroscopy	Shockwave lithotripsy	β (95% CI)
PROMIS pain intensity	44.4 (40.4, 48.4)	40.2 (38.6, 41.9)	42.4 (37.7, 47.2)	42.7 (41.2, 44.2)	-2.6 (-7.2, 2.1)
PROMIS pain interference	52.2 (47.3, 57.2)	44.2 (42.1, 46.3)	50.2 (44.9, 55.6)	48.6 (46.5, 50.6)	-1.3 (-6.1, 3.6)
PROMIS anxiety	45 (39.9, 50.1)	46.6 (44.5, 48.7)	45.3 (40, 50.6)	46.1 (44.2, 48)	-0.1 (-4.8, 4.7)
PROMIS peer relationships	51.2 (49.1, 53.2)	47.9 (46.2, 49.5)	48.9 (43.6, 54.2)	47.7 (45.9, 49.5)	-0.6 (-6.3, 5.1)
PROMIS sleep disturbances	52.4 (47.1, 57.7)	52.7 (50.9, 54.5)	51.5 (46.3, 56.8)	52.3 (50.6,54)	-1.7 (-5.3, 1.9)
PROMIS stress experiences	49.8 (44.5, 55.1)	50.6 (48.6, 52.6)	50.5 (46.7, 54.3)	49.3 (47.4, 51.2)	1.2 (-1.7, 4.1)
DVSS score† (urinary symptoms)	5.3 (3.7, 6.8)	5.7 (5.1, 6.4)	4.9 (3.7, 6)	5.5 (4.8, 6.1)	-0.5 (-1.5, 0.6)
QUIKSS‡ score (urinary symptoms)	11.5 (7.4, 15.7)	8.4 (6.9, 10)	11.5 (7.6, 15.4)	10.7 (9.4, 12)	-1 (-4.3, 2.3)

Weighted means with 95% CI.

† The range of the Dysfunctional Voiding Symptom Score (DVSS) is 0 to 30.

‡ The range of the Questionnaire for Urinary Issues – Kidney Stone Surgery (QUIKSS) is 0 to 56. QUIKSS is comprised of 16 items that used a frequency response scale and a 7-day recall period. Each item was scored from never (0) to always (4). The total score was the sum of the items.

© 2025 Tasian GE et al. *JAMA Network Open*.

eTable 17. Characteristics of Demographics and Affected Body Regions, Defined by the Pediatric Medical Complexity Algorithm, of Patients Aged 8 to 21 Years Who Had Ureteroscopy or Shockwave Lithotripsy During the Same Period at PKIDS Sites and Non-PKIDS Sites in PCORnet

	**PKIDS Responding Sites**		**Non-PKIDS Responding Sites**	
**Characteristics**	**Ureteroscopy No. (%)**	**Lithotripsy No. (%)**	**Ureteroscopy No. (%)**	**Lithotripsy No. (%)**
Number of unique Patients	1,252	211	2,008	227
Age at surgery				
Mean, Std Dev	15.5 (3.2)	15.7 (3.4)	17.5 (2.9)	17.8 (2.6)
8 - 11	194 (15)	32 (15)	143 (7)	11 (5)
12 - 15	343 (27)	54 (26)	297 (15)	21 (9)
16 - 18	433 (35)	66 (31)	582 (29)	85 (37)
19 - 21	282 (23)	59 (28)	986 (49)	110 (48)
**Sex**				
Male	479 (38)	93 (44)	724 (36)	103 (45)
Female	772 (62)	118 (66)	1282 (64)	124 (55)
**Hispanic**				
No	1018 (81)	175 (83)	1565 (78)	185 (81)
Yes	174 (14)	<30 (<14)	384 (19)	31 (14)
Other	60 (5)	<11 (masked)	59 (3)	11 (5)
**Race**				
African American or Black	67 (5)	<11 (masked)	159 (8)	11 (5)
Alaska Native or American Indian	<11 (masked)	<11 (masked)	<11 (masked)	0 (0)
Asian	17 (1)	<11 (masked)	17 (1)	<11 (masked)
Multiple Races	19 (2)	<11 (masked)	24 (1)	0 (0)
Native Hawaiian or Other Pacific Islander	<11 (masked)	0 (0)	<11 (masked)	<11 (masked)
White	979 (78)	170 (81)	1559 (78)	178 (78)
Other	162 (13)	25 (12)	238 (12)	36 (16)
**PMCA Body Region**				
Cardiac	157 (13)	20 (9)	243 (12)	13 (6)
Craniofacial	12 (1)	<11 (masked)	11 (1)	0 (0)
Dermatological	57 (5)	<11 (masked)	61 (3)	<11 (masked)
Endocrinological	131 (10)	25 (12)	196 (10)	14 (6)
Gastrointestinal	193 (15)	29 (14)	215 (11)	14 (6)
Genetic	65 (5)	<11 (masked)	45 (2)	<11 (masked)
Genitourinary	149 (12)	19 (9)	168 (8)	13 (6)
Hematological	95 (8)	14 (7)	87 (4)	<11 (masked)

© 2025 Tasian GE et al. *JAMA Network Open*.

	PKIDS Responding Sites		Non-PKIDS Responding Sites	
**Characteristics**	**Ureteroscopy No. (%)**	**Lithotripsy No. (%)**	**Ureteroscopy No. (%)**	**Lithotripsy No. (%)**
Immunological	79 (6)	<11 (masked)	83 (4)	<11 (masked)
Malignancy	39 (3)	<11 (masked)	64 (3)	<11 (masked)
Mental health	342 (27)	51 (24)	513 (26)	49 (22)
Metabolic	225 (18)	36 (17)	279 (14)	21 (9)
Musculoskeletal	227 (18)	39 (18)	265 (13)	25 (11)
Neurological	267 (21)	40 (19)	283 (14)	24 (11)
Ophthalmological	125 (10)	18 (9)	112 (6)	<11 (masked)
Otolaryngological	<11 (masked)	<11 (masked)	<11 (masked)	0 (0)
Otologic	61 (5)	16 (8)	59 (3)	<11 (masked)
Pulmonary	293 (23)	41 (19)	385 (19)	32 (14)
Renal	782 (62)	85 (40)	1301 (65)	110 (48)

Acronyms: Standard Deviation, Std Dev
Race and ethnicity were self-reported by participants

© 2025 Tasian GE et al. *JAMA Network Open*.
