# Supplementary material for: Ureteroscopy vs Shockwave Lithotripsy to Remove Kidney Stones in Children and Adolescents: A Nonrandomized Clinical Trial
Source: JAMA Netw Open. 2025 Aug 7;8(8):e2525789. doi: 10.1001/jamanetworkopen.2025.25789 (PMC12332628; doi:10.1001/jamanetworkopen.2025.25789)
Supplement: Supplement 2. — Trial Protocol and Statistical Analysis Plan [file jamanetwopen-e2525789-s002.pdf]

Title:	**Pediatric KIDney Stone (PKIDS) Care Improvement Network**
Short Title	PKIDS
Sponsor:	Patient – Centered Outcomes Research Institute (PCORI)
eIRB Number:	19-016832
Protocol Date:	November 26, 2019

Project Principal Investigator

Gregory Tasian, MD, MSc, MSCE
The Children's Hospital of Philadelphia
Division of Urology, 3rd Floor Wood Building
Philadelphia, PA 19104
Phone 215.590.2754
email: tasiang@chop.edu

Site Principal Investigator
Gregory Tasian, MD, MSc, MSCE
The Children's Hospital of Philadelphia
Division of Urology, 3rd Floor Wood Building
Philadelphia, PA 19104
Phone 215.590.2754
email: tasiang@chop.edu

TABLE OF CONTENTS

Table of Contents .... **ii**
Abbreviations and Definitions of Terms .... **iv**
Abstract .... **v**
Table 1: Schedule of Study Procedures Patient Participant * .... **vi**
Table 2: Schedule of Study Procedures Surgeon Participant * .... **vi**
Figure 1: PKIDS Organizational CHART .... **vii**
Figure 2: Patient Participant Flow........**viii**

1 BACKGROUND INFORMATION AND RATIONALE **1**
1.1 INTRODUCTION 1
1.2 RELEVANT LITERATURE AND DATA 1
1.3 COMPLIANCE STATEMENT 3

2 STUDY OBJECTIVES **4**
2.1 PRIMARY OBJECTIVE (OR AIM) 4
2.2 SECONDARY OBJECTIVES (OR AIM) 4
2.3 SECONDARY OBJECTIVES (OR AIM) 4

3 INVESTIGATIONAL PLAN **4**
3.1 GENERAL SCHEMA OF STUDY DESIGN 5
3.2 STUDY DURATION, ENROLLMENT AND NUMBER OF SITES 5
3.2.1 Total Number of Study Sites/Total Number of Subjects Projected *5*
3.2.2 Duration of Study Participation *5*
3.2.3 Total Number of Study Sites/Total Number of Subjects Projected *5*
3.3 STUDY POPULATION 6
3.3.1 Inclusion Criteria *6*
3.3.2 Exclusion Criteria *6*
3.4 EXECUTIVE COMMITTEE 7
3.5 STEERING COMMITTEE 7
3.6 PATIENT AND FAMILY PARTNERS (PFP) 7
3.7 PKIDS ENGAGEMENT CORE 7
3.8 CLINICIAN COUNCIL 7
3.9 PRIVATE SECTOR ADVISORY GROUP 8

4 STUDY PROCEDURES **9**
4.1 SCREENING VISIT 9
4.2 CONSENT 9
4.3
PARTICIPANT QUESTIONNAIRES AND ULTRASOUND 9
4.3.1 Questionnaires *9*
4.3.2 Ultrasound *10*
4.4 OPERATIVE SUMMARY 10
4.5 EHR SURVEILLANCE 10
4.6 UNSCHEDULED VISITS 10
4.7 SUBJECT COMPLETION/WITHDRAWAL 10
4.7.1 Early Termination Study Visit *10*

5 STUDY EVALUATIONS AND MEASUREMENTS **12**
5.1 MONITORING EVALUATIONS AND MEASUREMENTS 12
5.1.1 Screening *12*
5.1.2 Medical Record Review (Baseline) and EHR Surveillance (during duration of follow-up at each site) *12*

5.2 PARTICIPANT QUESTIONNAIRES 12
5.3 OPERATIVE SUMMARY 13
5.4 ULTRASOUND 13
5.5 SURGEON AND SITE CHARACTERISTICS 14

6 STATISTICAL CONSIDERATIONS 15

6.1 PRIMARY ENDPOINT 15
6.2 SECONDARY ENDPOINTS 15
6.3 CONTROL OF BIAS AND CONFOUNDING 15
6.3.1 Baseline Data 16
6.3.2 Analysis of Primary Outcome of Interest 16
6.4 SAMPLE SIZE AND POWER 17

7 SAFETY MANAGEMENT 18

7.1 CLINICAL ADVERSE EVENTS 18
7.2 ADVERSE EVENT REPORTING 18
7.3 DATA COLLECTION AND MANAGEMENT 18
7.3.1 Arcus 19
7.3.2 PEDSnet 20
7.4 CONFIDENTIALITY 21
7.5 REGULATORY AND ETHICAL CONSIDERATIONS 21
7.5.1 Data and Safety Monitoring Plan 21
7.5.2 Risk Assessment 22
7.5.3 Potential Benefits of Study Participation 22
7.5.4 Risk-Benefit Assessment 22
7.6 RECRUITMENT STRATEGY 23
7.7 INFORMED CONSENT/ASSENT AND HIPAA AUTHORIZATION 23
7.7.1 Waiver of Consent 24
7.7.2 Waiver of Assent 24
7.7.3 Alteration of HIPAA Authorization to Obtain Verbal Authorization 24
7.7.4 Waiver of HIPAA Authorization 24
7.8 PAYMENT TO SUBJECTS/FAMILIES 24
7.8.1 Payments to subject for time, effort and inconvenience (i.e. compensation) 24

8 PUBLICATION 25

9 REFERENCES 26

Appendix 30

ABBREVIATIONS AND DEFINITIONS OF TERMS

PKIDS	Pediatric KIDney Stone Care Improvement Network
URS	Ureteroscopy
SWL	Shockwave lithotripsy
PCNL	Percutaneous nephrolithotomy
PROMIS	Patient-Reported Outcomes Measurement Information System
PROs	Patient Reported Outcomes
AE	Adverse event
HTE	Heterogeneity of Treatment Effect
DCC	Data Coordinating Center
ED	Emergency Department
CI	Confidence Interval
SEM	Standard Error of the Mean

ABSTRACT

Context:

The rapid increase in the incidence of kidney stones among youth has resulted in a large population of patients who require surgery to removes stones but for whom little evidence exists to guide clinical care. Proper selection of surgical treatment options, which is directed by patient-specific factors and individual treatment goals, is the greatest determinant of successful outcomes.

Objectives:

Specific Aim 1: To compare stone clearance, retreatment, and unplanned healthcare encounters for ureteroscopy, shockwave lithotripsy, and percutaneous nephrolithotomy.

Specific Aim 2: To compare patients' experiences after ureteroscopy, shockwave lithotripsy, and percutaneous nephrolithotomy.

Study Design:

Prospective cohort study.

Setting/Participants:

This study will be conducted by the Pediatric KIDney Stone (PKIDS) Care Improvement Network, which includes 20 pediatric healthcare systems (22 sites) in the United States.

Patient participants include patients aged 8 to 21 years who undergo a surgical intervention for kidney stones as part of their clinical care. The surgical interventions include ureteroscopy, shockwave lithotripsy, and percutaneous nephrolithotomy, all of which would be performed as part of routine clinical care.

Surgeon participants include practicing urologists who will perform the patient participant surgery.

Study Interventions and Measures:

Stone clearance will be assessed with ultrasound (obtained as part of clinical care) 6 weeks after surgery, which is the standard of care at all PKIDS sites. Retreatment and unplanned healthcare encounters will be assessed at 3 months after surgery. Patients' experiences will be measured through questionnaires within 1 week of surgery, 3 weeks after surgery, and 3 months after surgery (**Table 1**).

Surgeon Participant Questionnaire **(Table 2).**

TABLE 1: SCHEDULE OF PROCEDURES PATIENT PARTICIPANT *

**Study Phase**	**Pre-op**		**Patient-Reported Outcomes**				**Clinic Visit**
**Visit Number**		**Base-line**	**1 week postop**	**3 weeks post op**	**6 weeks postop**	**3 months postop**	**6 weeks**
**Study Days**							
Informed Consent/Assent	X						
Review Eligibility Criteria	X						
Demographics/ Medical History	X						
Participant questionnaires		X	X	X	X	X	
Ultrasound							X
Adverse Event Assessment			X	X	X	X	X

* All procedures, including surgical intervention and ultrasound, will be performed as part of standard clinical care. The only study procedures that are not part of clinical care is the review of medical records and Patient-Reported Outcomes assessments.

TABLE 2: SCHEDULE OF PROCEDURES SURGEON PARTICIPANT*

**Study Phase**	**Pre-op**
Review Eligibility Criteria	X
Consent/Assent<br>(Waiver of Documentation Informed Consent)	X
Surgeon Participant Questionnaire	X

FIGURE 1: PKIDS ORGANIZATIONAL CHART

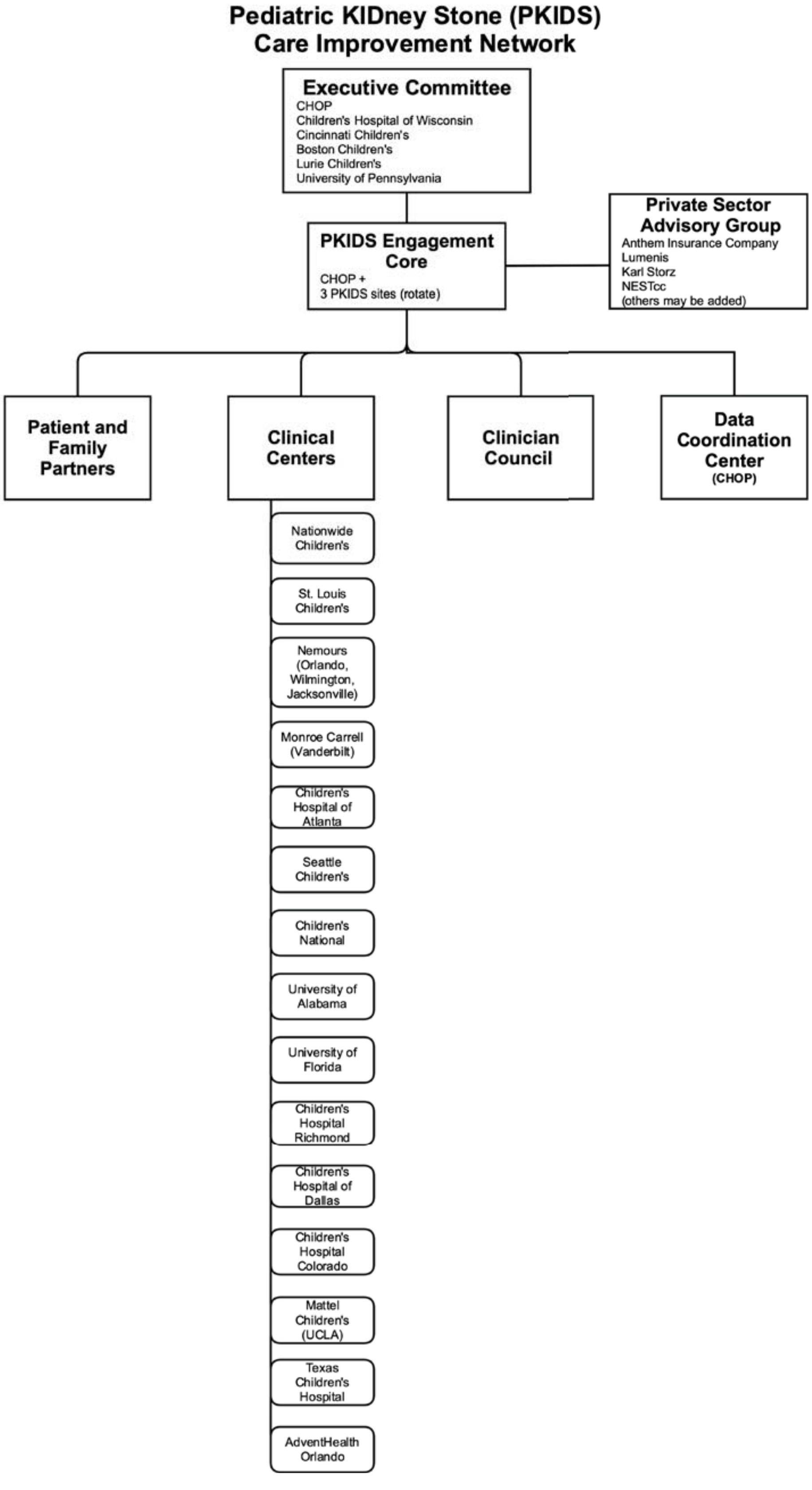

FIGURE 2: PATIENT PARTICIPANT FLOW

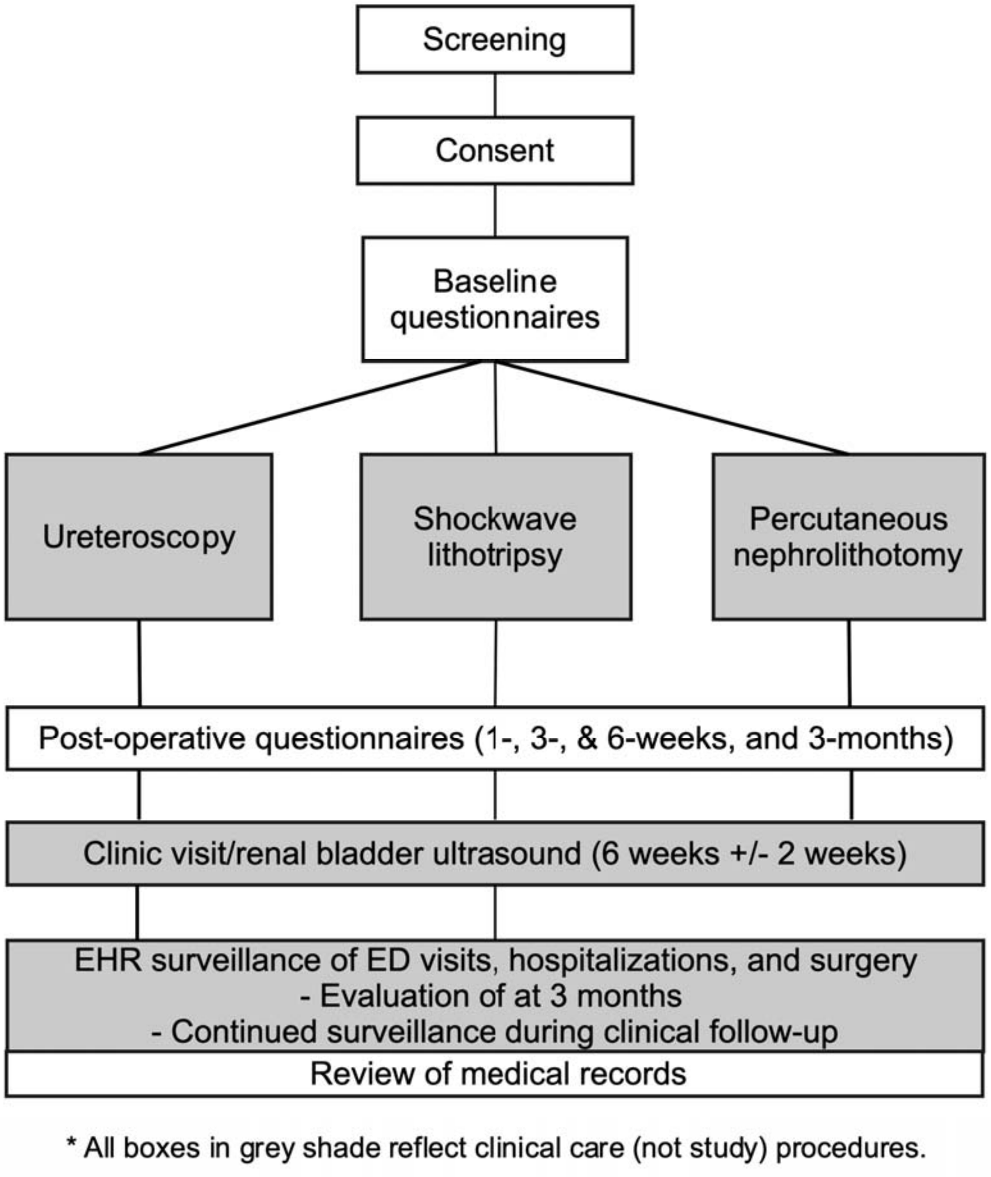

1 BACKGROUND INFORMATION AND RATIONALE

1.1 Introduction

The rapid increase in the prevalence of kidney stones among youth has resulted in a large population of patients for whom little evidence exists to guide clinical care. This proposal arises from patients' expressed need to better understand the effectiveness of different surgical approaches to remove kidney stones, the impact of these surgeries on their lives, and how long it takes to recover. To address this critical knowledge gap, this proposal leverages the Pediatric KIDney Stone (PKIDS) Care Improvement Network, which is a community of patients, caregivers, and clinicians who perform collaborative studies to generate knowledge about kidney stone disease. PKIDS arose out of the 8 institutions that comprise PEDSnet, a member of the PCORI-funded PCORnet, and now includes 20 pediatric healthcare systems (22 sites) in the United States. PKIDS leverages the PEDSnet infrastructure to efficiently perform studies and translate the findings into clinical practice. The long-term goal of PKIDS is to generate and apply knowledge that improves the lives of pediatric patients with kidney stones. The immediate objective of the proposed study is to compare stone clearance and patients' experiences for the three existing surgical treatments to remove stones: ureteroscopy (URS), shockwave lithotripsy (SWL), and percutaneous nephrolithotomy (PCNL). The resultant knowledge of these patient-centered outcomes will improve pre- and intra-operative decision-making about the surgical management of stones. In the proposed prospective cohort study, we apply a conceptual framework for the examination of procedural interventions that accounts for the patient, surgeon, technical, hospital, and access factors that influence the choice of surgery and outcomes for patients 8 to 21 years of age.[1] Accordingly, we will prospectively measure characteristics that impact the real-world delivery of surgical care and its outcomes in 20 pediatric healthcare systems and examine how application of knowledge generated improves outcomes for patients.

1.2 Relevant Literature and Data

Kidney stones (nephrolithiasis) are painful, affect 9% of the United States (US) population,[2] and result in annual healthcare costs of over $10 billion,[3] exceeding treatment costs for prostate cancer and urinary tract infection (UTI) in women by more than 3 and 5 billion dollars, respectively.[4] Kidney stones decrease quality of life[5-7] and are associated with low bone mineral density and fracture,[8,9] chronic kidney disease,[10] and heart disease.[11] The prevalence of kidney stones in the US increased from 5% (1988 to 1994) to 9% (2007 to 2010).[2,12] Among all age groups, the greatest increase has been in adolescents;[13-15] the annual incidence of stones among girls aged 15 to 19 years increased from 75 per 100,000 in 1997 to 120 per 100,000 in 2012.[16] The prevalence of kidney stones among children and adolescents is greater than that of pediatric diabetes and has increased at a higher rate.[17] This rising incidence has resulted in more surgeries, hospitalizations, and emergency department (ED) visits,[14,18] which caused a 53% increase in healthcare expenditures for pediatric patients between 1997 and 2012.[19,20] The true economic

burden is higher when imaging, outpatient visits, medications, and missed work/lost wages by caregivers are considered. Pediatric kidney stones have a high recurrence risk. Approximately 50% of children develop recurrent stones within 3 years,[21,22] which is higher than rates reported for adults.[23,24] Compared to adult-onset nephrolithiasis, children with kidney stones have a greater lifetime risk of needing surgery and its associated morbidity.

Up to 60% of children with kidney or ureteral stones (hereafter referred to as kidney stones) require surgery.[25-27] As for adults, surgery is indicated when a ureteral stone does not pass and for non-obstructing kidney stones that are too large to pass, cause infection, pain, or hematuria, and when patients choose treatment over surveillance. Surgical options include ureteroscopy (URS), shockwave lithotripsy (SWL), and percutaneous nephrolithotomy (PCNL), all of which use an energy source to break stones and require anesthesia.[27] URS is an outpatient endoscopic surgery that accesses the ureters and kidneys per the urethra to fragment and remove the stones. SWL is a non-invasive outpatient procedure that targets stones in the kidney or ureter with x-ray or ultrasound and uses shockwaves generated outside the body to fragment them; the fragments pass down the ureter and the patient expels them in the urine. PCNL is a minimally invasive surgery in which a ~1cm flank incision is made, and a scope is passed through a tubular sheath into the kidney to fragment and remove the stone. There is typically a 1- to 2-night hospital stay. Many patients who undergo URS, SWL, or PCNL also have temporary tubes placed after the surgery to facilitate drainage, either internally (a stent) or externally (a nephrostomy tube). Proper treatment selection, which is directed by patient-specific factors and individual treatment goals, is the greatest determinant of successful outcomes.

Stone clearance is a surgical outcome that is important to patients because residual stone fragments lead to painful stone passage, ED visits, and surgical retreatment.[28-32] The comparative effectiveness of URS, SWL, and PCNL with respect to stone clearance for pediatric patients is unclear because of five characteristics of prior studies.

- Biased measurement of stone clearance due to use of multiple imaging modalities with different sensitivities.
- Combining patients who had repeated treatments with those who had one treatment in a single group.
- Heterogeneity of the size of residual stone fragments after surgery considered “clinically insignificant”.
- No consideration of factors impacting treatment choice and outcomes (*e.g.,* surgeon characteristics).
- Preponderance of studies arising in areas of endemic stone disease (*i.e.,* Turkey and China) that reflect a high institutional and surgeon experience. It is unclear whether these results are generalizable to the US, where nephrolithiasis among children has become more common only recently.

This weak evidence base regarding stone clearance is reflected in the 2016

American Urological Association and Endourological Society guidelines for the surgical management of urinary stones, which were based on a systematic review of the literature. Of the 90 recommendations, only eight addressed pediatric patients.[33,34] Of these eight recommendations, four were based only on expert opinion (no evidence).

Understanding pediatric patients' experiences after procedures to remove stones would improve the ability of patients and their caregivers to select treatment options. Indeed, the 2016 guidelines recommend including "patient preferences and personal goals" in choosing a management strategy.[33,34] It is clear from the collective clinical experience of urologists in PKIDS, from adult studies, and from conversations with patients that the impact of URS, SWL, and PCNL on physical, social, and mental health differs.[35] However, *how, to what degree, and for how long* these three treatment modalities differ is unknown. Unless there is adequate evidence on post-operative experiences, pediatric patients and their caregivers will not be able to make decisions that take into account outcomes that are most important to them.

Fewer RCTs have been published for the management of kidney stones than for any other renal disease, demonstrating the difficulty in completing RCTs for nephrolithiasis and the need for high-quality evidence to inform treatment selection.[36] Furthermore, the incomplete knowledge of stone clearance and lived experiences is compounded by unique considerations of pediatric patients. These include more ancillary procedures (*e.g.,* greater need for ureteral stent placement), anatomic characteristics that may alter the efficacy of stone removal (*e.g.*, acute intrarenal angles, smaller body mass), and psychosocial dynamics that make pediatric experiences different than adults. In response, the American Urological Association specifically called for high-quality studies that compare stone surgery outcomes for pediatric patients.[37]

1.3 Compliance Statement

This study will be conducted in full accordance all applicable Children's Hospital of Philadelphia Research Policies and Procedures and all applicable Federal and state laws and regulations including 45 CFR 46. All episodes of noncompliance will be documented.

The investigators will perform the study in accordance with this protocol, will obtain consent and assent, and will report unanticipated problems involving risks to subjects or others in accordance with The Children's Hospital of Philadelphia IRB Policies and Procedures and all federal requirements. Collection, recording, and reporting of data will be accurate and will ensure the privacy, health, and welfare of research subjects during and after the study.

2 STUDY OBJECTIVES

The goals of this research are to improve the ability of pediatric patients and their caregivers to select surgical treatment options for kidney stones and to enable urologists to use techniques that result in the best outcomes for these surgeries.

2.1 Primary Objective (or Aim)

The primary objective is to compare stone clearance for URS, SWL, and PCNL. We will examine heterogeneity of treatment effect (HTE) by clinical status (stone size and stone location). We will also identify modifiable surgical techniques associated with higher stone clearance for each surgery.

2.2 Secondary Objectives (or Aim)

The secondary objective is to compare patients' experiences after URS, SWL, and PCNL. We will assess the immediate (within 7-days +/- 3 days) and delayed (21-days +/- 7-days, 45-days +/- 7-days, and 90 days +/- 30 days) impact of surgery on patient-reported outcomes (PROs) selected by PKIDS patient partners. The PROs include the PROMIS, other questionnaires commonly used in studying the impact of kidney stone disease on health states, and urinary tract symptoms. We will examine HTE by age and sex and identify modifiable surgical techniques that impact these experiences for each surgery. We will also determine unplanned hospitalizations, ED visits, and repeat surgeries for the 3 months following each procedure.

2.3 Tertiary Objectives (or Aim)

We will address the following secondary objectives

1. To assess the relative diffusion, application, and impact of derived best practices from study results across all patients with kidney stones in the PKIDS site network.
2. To determine the association between findings on post-operative renal ultrasound and clinical outcomes including ED visits and other unplanned healthcare encounters.
3. To determine the long-term health care experience of patients with kidney stone disease after surgery.

3 INVESTIGATIONAL PLAN

The proposed prospective cohort study will compare stone clearance and the lived experiences for pediatric patients after URS, SWL, and PCNL while also measuring the longitudinal impact of kidney stone disease. HTE will be examined by clinical status (stone size, stone location) and patient characteristics (age and sex). The results will provide information that helps patients make individualized decisions about the best surgical approach. Surgical techniques specific to URS, SWL, and PCNL that improve stone clearance and patients' experiences for each procedure will also be identified.

3.1 General Schema of Study Design

The proposed project will generate knowledge about alternative surgical treatments to remove kidney stones in patients 8 to 21[1] years of age (at time of enrollment) at the 22 sites in the PKIDS Care Improvement Network. This prospective follow-up cohort study compares stone clearance at 6 weeks (Aim 1) and patients' experiences at 3 months (Aim 2) after URS, SWL, and PCNL, examining for HTE by clinical status and patient characteristics. We will also identify modifiable techniques that improve stone clearance and patients' experiences for each type of surgery. We will follow patient participants through EHR surveillance for the duration of their clinical care at each institution to understand the long-term health care experience of patients with kidney stone disease after surgery.

3.2 Study Duration, Enrollment and Number of Sites

3.2.1 Total Number of Study Sites/Total Number of Subjects Projected

Participants will be enrolled at 20 pediatric healthcare systems (22 sites including 3 sites in the Nemours healthcare system). The target sample size is 1200 participants in the initial comparative effectiveness study (Aims 1 & 2). It is projected that approximately 1600 patients will be screened and approximately 1600 of those patients will be eligible, of whom approximately 1290 would be enrolled. The final sample size of evaluable patient participants who have completed all outcome measures is approximately 1120. The final sample size of evaluable surgeon participants is approximately 60. Surgeon and patient participants will continue to be enrolled during the knowledge implementation phase, which is when the knowledge of best practices generated by the comparative effectiveness study is integrated into clinical care.

3.2.2 Duration of Study Participation

The duration of the study for the enrolled patient participants is the time of consent to the end of follow-up within PKIDS. Patient participants will complete questionnaires before surgery (baseline), within 7 days of surgery (+/- 3 days), 21-days +/- 7-days, 45-days +/- 7-days, and 90 days +/- 30 days after surgery. Ultrasound (as part of clinical care) will be obtained 6 weeks (+/- 2 weeks) after surgery. EHR surveillance of ED visits, clinic visits, hospitalizations, and surgeries will occur during the duration of clinical follow-up in PKIDS. Surgeon participants will complete one-time questionnaires assessing individual and institution characteristics.

3.2.3 Total Number of Study Sites/Total Number of Subjects Projected

The study will be conducted at 20 healthcare systems in the United States. One of the healthcare systems, Nemours, has three geographically distinct sites in Wilmington, DE; Orlando, FL; and Jacksonville, FL. No human subjects research activities will be conducted at other study sites until an IRB reliance agreement is executed.

Accounting for a 10% dropout prior to study completion, we expect to approach approximately 1600 individuals to ensure we have approximately 1120 patient participants with evaluable data at the end of the comparative effectiveness aims. We will continue to enroll participants as knowledge generated from the comparative effectiveness arm (i.e. best practices) is integrated into clinical care. This aim does not have a set number of projected participants as its focus is continuous quality improvement.

3.3 Study Population

3.3.1 Inclusion Criteria

1) Patient Participant
    a. Males or females, 8-21 years of age, undergoing planned URS, SWL, or PCNL for the removal of at least one kidney and/or ureteral stone.

    b. Parental/guardian or participant (if ≥ 18 years old) permission (informed consent), and if appropriate, child assent
        i. Individuals who are not able to provide consent/assent (whether ≥18 or < 18 years) and/or not willing or able to complete questionnaires are eligible for participation for the stone clearance assessment and EHR surveillance if the legal guardian consents for study participation.
        ii. Individuals for whom native-language questionnaires are not available can also participate in stone clearance assessment and EHR surveillance.
2) Surgeon Participant
    a. Practicing urologist who will perform the patient participant surgery

3.3.2 Exclusion Criteria for patient participants

1) Patients for whom conducting informed consent and baseline study procedures would confer additional risk (e.g. obstructing ureteral stone with fever requiring emergency surgery) and delay necessary immediate clinical care.

2) Parent/guardians or patients, who, in the opinion of the Investigator, may be non-compliant with study schedules or procedures

Individuals who had a ureteral stent and/or nephrostomy tube placed at an outside institution and plan to have URS, SWL, or PCNL at a PKIDS site are eligible for inclusion. Individuals who have a ureteral stent placed at the time of planned URS due to inability to access the ureter (i.e. pre-stenting) are eligible for inclusion and would have PROs administered in the time frame described in section 4.3.1 after the “pre-stenting” and definitive surgical procedure. It is not necessary for a patient participant to have diagnostic imaging (e.g. ultrasound) obtained at the PKIDS site before surgery.

Any deviations from these criteria must be reported in accordance with IRB Policies and Procedures.

3.4 Executive Committee

The Executive Committee is comprised of the Principal Investigators at CHOP, Boston Children's, Cincinnati Children's, Lurie Children's, Children's Hospital of Wisconsin, and the University of Pennsylvania. The responsibilities of the Executive Committee are: 1) To ensure that operations are executed in an effective and timely fashion; 2) To keep all activities focused on the central mission of PKIDS; and 3) To create a culture of respectful collaboration where all partners and stakeholders feel empowered to freely share ideas.

3.5 Steering Committee

The PKIDS Steering Committee is comprised of the Principal Investigators at each of the clinical centers in PKIDS. The Steering Committee will be responsible for recruitment and engagement at each of the PKIDS sites and, along with the Executive Committee, be responsible for PKIDS operations.

3.6 Patient and Family Partners (PFP)

The PFP is a group of patients who had kidney stone surgery and their caregivers who represent the broad spectrum of patients and families served by PKIDS institutions. The PFP will ensure the co-production of all study activities, including revising and approving the protocol and manual of procedures. PFP members will receive Partnership-Based Research Training, will meet regularly, and will be paid consultant fees.

3.7 PKIDS Engagement Core

The PKIDS Engagement Core will establish recruitment and membership criteria for patients to participate as members of the PKIDS PFP. The members of the PKIDS PFP are not research participants, but rather partners in research. The PKIDS Engagement Core will develop and implement the strategic plan to increase patient and caregiver partners and stakeholder engagement in PKIDS, including: 1) recruiting PFP members at other PKIDS sites that accurately represent the broad spectrum of patients and families served by these institutions and 2) leveraging ongoing patient/participant engagement efforts at each PKIDS site to recruit PFP members. The PKIDS Engagement Core will consist of PKIDS team members from four organizations: CHOP and 3 sites that rotate yearly.

3.8 Clinician Council

The Clinician Council is a group of providers who care for patients with stones (for example, urology, radiology, nephrology) and represent the geographic locations, provider ages, and experience levels in PKIDS. The Council will provide strategic and tactical input about the study from front-line clinicians, review PKIDS site

practice patterns, and serve as experts in the engagement with clinicians in the community of practice.

3.9 Private Sector Advisory Group

The Private Sector Advisory Group includes, for example, payers and companies that make equipment used for stone surgery. This Advisory Group will continue to identify areas of common interest and offer suggestions for how to work more effectively with one another to improve patient care. The members of this group may expand as the study progresses.

4 STUDY PROCEDURES

Please see Table 1 and 2 (p. vi).

4.1 Screening Visit

Review of the electronic health record for potentially eligible patient participants will occur before consent is obtained.

- Electronic health record review
  - Date of birth
  - Past medical history
  - Past surgical history
  - Comorbidities
  - Clinician notes

4.2 Consent

a. Patients participants who are not hospitalized will be contacted before or at a clinic visit or on the day of surgery to inform them about the study. When feasible, patients who present to the ED will be included. Screened patient participants may be contacted by telephone or in-person to obtain consent. The informed consent process for in-person consent may occur at ambulatory clinic visits, inpatient hospitalizations, in the ED, or on the telephone. Patient participants can be enrolled either in-person or via telephone up to and including the day of surgery before the procedure. Only patients with a sufficient amount of time to consider study participation will be approached. On day of surgery no pre-medications which may impact their ability to consent or assent will be distributed prior to consent.
b. Surgeon participants will be approached before identifying patient participants. They will provide consent/assent (waiver of documentation) via REDCap by clicking on an “I Agree” button to then continue to complete the questionnaire.

4.3 Participant Questionnaires

All study participants will complete questionnaires.

4.3.1 Questionnaires

Patient participants will complete a baseline questionnaire that assesses individual characteristics, including but not limited to past medical history and family history, with particular emphasis on stone factors. Patient participants will complete questionnaires, including PROMIS measures and a urinary symptom questionnaire, before surgery (baseline), within 7 days of surgery (+/- 3 days), 21-days +/- 7-days, 45-days +/- 7-days, and 90 days +/- 30 days after surgery. The questionnaires include, but are not limited to, PROMIS instruments (pain intensity, pain interference, anxiety, psychological stress experiences) and a urinary symptom questionnaire. Questionnaires will be administered electronically and recorded in secure electronic databases including OnCore and REDCap, enabling completion at

home. For patients who do not have mobile devices or a computer, a member of the research team can administer the questionnaires over the phone or on paper.

Surgeon participants will complete one time questionnaires assessing individual and institution characteristics via REDCap.

4.3.2 Ultrasound (as part of clinical care)

As part of routine clinical care, patient participants will undergo an ultrasound 6 weeks (+/- 2 weeks) after surgery. The results of the ultrasound will be obtained via medical records/imaging review.

4.4 Operative Summary

At the end of the surgical case, the attending surgeon will complete an operative summary. The summary will contain details about the stone and technical details of the procedure and may be administered electronically or on paper. The results of the summary will be recorded in OnCore and/or REDCap by a member of the study team.

4.5 EHR Surveillance

Patient participant data from ED visits, hospitalizations, clinic visits, and surgical (re)treatment will be obtained during the duration of clinical follow-up in PKIDS. A member of the research team at each site will manually access the data at the PKIDS site and enter it into the secure electronic database (e.g. OnCore). For the 8 PEDSnet sites, outcomes will also be ascertained by querying the PEDSnet Common Data Model at each PEDSnet site. Patient participants may be contacted to verify information obtained about healthcare encounters that occurred within 3 months of the index surgical procedure.

4.6 Unscheduled Visits

This study does not involve any study visits; all visits are for clinical care. Unscheduled visits that are part of clinical care will be recorded. Unscheduled visits that are specific to the study are not expected. Should one occur, the PI will be notified and will ascertain the reason for the visit and ensure that all questions have been answered. Any AEs, which are not expected, will be noted and reported.

4.7 Subject Completion/Withdrawal

Participants may withdraw from the study at any time without prejudice to their care. The reason for withdrawal will be recorded. It will be documented whether or not each participant completes the clinical study. If investigator(s) become aware of any serious, related adverse events after the participant completes or withdraws from the study, those events will be recorded in the source documents.

4.7.1 Early Termination Study Visit

Participants who withdraw from the study will have all procedures enumerated for the study visit as the early termination visit.

5 STUDY EVALUATIONS AND MEASUREMENTS

5.1 Monitoring Evaluations and Measurements

There are three major data sources that will be used for analysis: (1) EHR of patient participant, (2) patient participant report, and (3) surgeon report.

5.1.1 Screening

Before consent is obtained, a member of the research team will use the EHR to assess for patient eligibility. A diverse population with respect to sex, race, geography, and comorbidities will be included, thereby assuring representation of the full spectrum of patients with stones in the US.

5.1.2 Medical Record Review (Baseline) and EHR Surveillance (during duration of follow-up at each site)

The variables that may be obtained from the medical record at baseline include, but are not limited to, the following:

- Date of birth
- Weight
- Height
- Race and ethnicity
- Sex
- Past medical history, include specialty and acute care utilization
- Past surgical history, including operative reports
- Family history
- Medications
- Diagnoses
- Healthcare payer
- Diagnostic imaging

The EHR will be surveilled for clinic visits, ED visits, unplanned hospitalizations, and surgical retreatment. Data from institutions in PEDSnet may be obtained using an automated data pull. Research team members at each site may manually abstract the data and enter it into REDCap and/or OnCore.

5.2 Participant Questionnaires

Patient participants will complete questionnaires that include PROMIS measures, quality of life instruments, and a urinary symptom questionnaire. These questionnaires will be administered before surgery (baseline), within 7 days of surgery (+/- 3 days), 21-days +/- 7-days, 45-days +/- 7-days, and 90 days +/- 30 days after surgery. The questionnaires may be distributed electronically or administered over the phone or on paper. The baseline questionnaires may be completed at any time between enrolling in the study and the surgery, including the day of surgery before the procedure.

Based on patient interviews, the anticipated PROMIS profile may include pain intensity, pain interference, anxiety, psychological stress experiences, peer relationships, and family relationships.[38] The PFP will approve the final profile. All questionnaires will be administered and recorded in REDCap.

Surgeon participants will complete one time questionnaires via REDCap assessing individual and institution characteristics.

.

5.3 Operative Summary

The attending surgeon will fill out an operative summary that includes a questionnaire regarding the kidney stone and operative techniques used during the kidney stone surgery immediately after the procedure is finished. These items include:

1) Procedure type
2) Lithotripsy method
3) Energy settings, if applicable
4) Ancillary equipment such as stents, catheters, or drains
5) Operative findings
6) Intra-operative imaging parameters, if applicable

The surgeon can complete an electronic or paper version of the summary, and the results will be stored in OnCore. The PKIDS surgeons will be trained on how to use the summary in order to ensure standardization of the reporting. Ultimately, a templated version of the summary could be developed in the EHR that will include information from the checklist.

5.4 Ultrasound

Stone clearance will be assessed with an ultrasound (as part of clinical care) 6 weeks (+/- 2 weeks) after surgery, which is the current standard of clinical care at all sites. Radiologists at PKIDS sites will document findings in radiology reports. Coordinators will abstract standardized radiology reports and record results in OnCore.[52] Ultrasound images coded with a PKIDS ID for all CHOP will be uploaded to Arcus.

To assess and improve inter-observer reliability, a random sample of ultrasounds will be reviewed centrally by a pediatric radiologist blinded to the surgery. Images coded with the PKIDS ID will be uploaded remotely to CHOP's Arcus data research infrastructure. Arcus will provide a secure, cloud-based platform to receive, store, and analyze these images. Please see Section 7 for details about Arcus data security and confidentiality. Centralized review will occur more frequently in the first 6 months of the study to allow for quality assessment and improvement.

Discrepancies between local and central interpretations will be reconciled and the reasons for any discrepancy will be identified to improve ultrasound interpretation across PKIDS.

5.5 Surgeon and site characteristics

In addition to the participant-facing measurements described below, urologists at PKIDS sites will complete questionnaires via REDCap about their surgical training, operative experience, preferences for particular kidney stone surgeries, access to kidney stone surgical equipment, techniques used during surgery, and importance of secondary factors related to surgery for children (e.g. radiation exposure, need for ancillary procedures, hypertension). In addition, they will provide information about the resources and environment of their healthcare system, including ownership of surgical equipment and relationships to lithotripsy centers.

6 STATISTICAL CONSIDERATIONS

6.1 Primary Endpoint

Stone Clearance

6.2 Secondary Endpoints

Patients' Experiences.

6.3 Control of Bias and Confounding

While all patients meeting eligibility criteria may be enrolled, we will restrict analysis to the first kidney stone surgery to avoid introducing clustering at the patient-level for the few (likely more complex) patients who would have multiple stone events over the 21-month enrollment period. Considering the large number of potential confounders, a pre-specified list of characteristics that includes but is not limited to the variables in Table 2 will be ascertained before surgery.

**Patient-level**	**Surgeon-level**
Patient demographics	Years of out of residency
Body mass index	Experience with SWL, URS, and PCNL
Pain at stone presentation	Endourology fellowship
Insurance type	Pediatric urology fellowship
Number of stones	Preferences for URS, SWL, and PCNL
Type of stone	Ownership in SWL collaborative
Single location vs. multicentric	Ownership of surgery center
UTI at presentation or history of UTI	Surgeon gender
Neurogenic bladder	Preferences for particular surgery
Urinary tract anomaly	Importance of factors in surgery choice (radiation, reimbursement, discomfort)
Expectations about surgery	
Prior urinary tract reconstruction	**Hospital-level**
Hydronephrosis	Hospital characteristics (e.g. bed number, nursing staffing)
Co-morbid conditions	Annual number of URS, SWL, PCNL performed at the hospital
Prior kidney stone surgery	Interventional radiology available
Acute/urgent procedure or elective	SWL machine owned by hospital
Prior diversion for the stone episode	Laser ownership
Ambulatory status	Kidney Stone Center
Pre-op imaging type	Trainee involvement in surgery
Pre- and post-operative medications	Free-standing children's hospital

We will construct propensity score models based on these confounders, and use an inverse probability treatment weighting approach for multi-level data to balance covariates across treatments at patient- surgeon- and hospital-levels in evaluation of overall treatment effect and HTE .[62] As we compare three treatments, we will use multinomial logistic regression with random effects to calculate propensity scores.[63-65] The random effects will allow us to account for correlation within surgeon- and hospital-levels. We assume there is overlap among the treatment groups such that every patient could have received each treatment. Under this assumption, the weighted treatment effects yield consistent estimates of the population averaged treatment effects, provided that the commonly assumed assumptions of consistency

and no unmeasured confounders in causal inference also hold.[54,66,67] We will examine overlap of covariates across treatment groups using plots of distributions of the estimated propensity scores across groups, and examine the balance of covariates using standardized difference in mean before and after weighting.[68-70] We will also conduct sensitivity analyses using both simulation-based approaches[71] and propensity score-based sensitivity functions[72] to assess the effect of potential unmeasured confounding on the estimates of the parameters of interest.

Statistical Methods

The statistical methods will be described in detail in the Statistical Analysis Plan.

6.3.1 Baseline Data

Baseline and demographic characteristics will be summarized by standard descriptive summaries (e.g. means and standard deviations for continuous variables such as age and percentages for categorical variables such as gender).

6.3.2 Analysis of Primary Outcome of Interest

Stone Clearance: Overall Treatment Effect The primary analysis of Aim 1 is to test the differences in stone clearance after surgery across treatment options. We will fit weighted logistic regression models for stone clearance measured at 6 weeks after surgery. We will use the likelihood ratio test to test the null hypothesis. We will report point estimates and 95% confidence intervals (CIs) of odds ratios in stone clearance between each pair of treatments.

> **SubAim 1a: Heterogeneity of Treatment Effect (HTE)**. We will add pairwise interactions between the treatment dummy variables and stone size and between treatment and stone location. We will also explore HTE by other patient participant characteristics including, but not limited to, ambulatory status.
>
> **SubAim 1b: Effect modification by procedure-specific techniques.** We will stratify the data by treatments and, within each treatment, we will fit logistic regression models with random intercepts to test whether modifiable techniques improve stone clearance.

Patient Experiences: Overall Treatment Effect.

For PROMIS measures, we will fit weighted linear mixed effects models to test the difference in T-scores after surgery across treatment options for each domain. Separate models will be built for outcomes at 1 week, 3 weeks, 6 weeks, and 3 months after surgery. We will also take advantage of the longitudinal nature of the PROs and fit WLMMs to compare the slopes of the PROs over time and the area under the curve.

Secondary patient experience outcomes, including number of emergency department visits, unplanned hospitalizations, and surgical retreatment will be compared across treatment options using weighted zero-inflated negative binomial regression models.

SubAim 2a: HTE. We will examine for HTE with interaction terms between procedure and patient age and procedure and sex to identify associations that differ for subgroups. We will also explore HTE by other patient participant characteristics including, but not limited to, ambulatory status.

SubAim 2b: Effect modification by procedure-specific techniques. We will stratify the data by treatments and, within each treatment; we will fit linear mixed effect models to test whether modifiable techniques are associated with patients' experiences for each procedure.

We will evaluate the change in outcomes across PKIDS after knowledge of best practices generated by the above analyses are integrated into clinical care. Our regression models will test differences in both the intercept (average value) and slope (change over time) for outcomes.

6.4 Sample Size and Power

Assumptions of sample size calculations. Our sample size calculations are based upon our primary aims – to compare stone clearance and patients' experiences of treatment options to remove kidney stones. We use Bonferroni adjustment to allow for multiple comparisons, which is known to be conservative.[73] The multiple comparisons are: difference in stone clearance across 3 treatment options and mean difference in PROs across 3 treatment options at 1 week, 3 weeks, 6 weeks, and 3-months after surgery. We assume a loss to follow up of 15%. We assume the propensity score model, which accounts for dependence of observations at the hospital and surgeon levels, controls for confounding such that patients whose propensity scores are equal have similar baseline covariates values. In the initial comparative effectiveness study, we anticipate enrolling approximately 750 patients in URS, 350 in SWL, and 100 in PCNL. As defined by Varadhan *et al.*, the goal of the proposed HTE analyses is descriptive: to report treatment effects for prespecified subgroups in accordance with a prospectively specified analytic strategy. Accordingly, descriptive HTE analyses need not be powered to detect heterogeneity because the goal is to report results for future synthesis.[56]

Aim 1: 294 patients in SWL, 294 patients in URS, and 92 patients in PCNL will have 80% power to detect an expected difference of 80%[74] vs. 65%[75] in stone clearance between URS and SWL, and to detect a difference of 90%[76] vs. 65%[75] in stone clearance between PCNL and SWL at α=0.05, accounting for multiple comparisons and loss to follow-up.

Aim 2: Minimal important differences for many PROMIS domains are in the range of 2 to 6. Assuming a 10% loss to follow-up, 39 patients in each group will have 80% power to detect an expected difference of 2 points in each of the 5 PROs across treatment options at α=0.05, adjusting for multiple comparisons.

7 SAFETY MANAGEMENT

7.1 Clinical Adverse Events

Clinical adverse events (AEs) will be monitored throughout the study.

7.2 Adverse Event Reporting

Since the study procedures are not greater than minimal risk, SAEs are not expected. If any unanticipated problems related to the research involving risks to subjects or others happen during the course of this study (including SAEs) these will be reported to the IRB in accordance with CHOP IRB SOP 408: Unanticipated Problems Involving Risks to Subjects. AEs that are not serious but that are notable and could involve risks to subjects will be summarized in narrative or other format and submitted to the IRB at the time of continuing review.

STUDY ADMINISTRATION

7.3 Data Collection and Management

Data Security and Privacy. Computer systems housing PKIDS databases and software are located in the CHOP research datacenters, which provides both physical and network security. Access to these systems is limited to authorized staff only, with credentials maintained via CHOP's central Active Directory system. PKIDS DCC database servers and development systems are connected only to the internal CHOP network, access to which is limited to CHOP staff and collaborators provided VPN access. Individual computer systems used by PKIDS DCC staff comply with CHOP standards for device encryption and security. Logs of other access to CHOP computer systems are maintained in accordance with CHOP information security policies. All research activities that uses PEDSnet core databases will adhere to established PEDSnet policies. All activity in the PEDSnet core database and query portals is logged, and logs are retained for auditing (please see 7.3.2. for PEDSnet-specific data procedures).

We estimate the risk of disclosing identifiable information without a person's consent, and the risk of harm consequent to such disclosure to be very low; no activity undertaken as part of this proposal involves greater than minimal risk to any participant. In addition, we will ensure participant confidentiality and privacy, and data security.

1. **Confidentiality**. Data/CRFs will be stored in OnCore Clinical Trials Management System, a CHOP Research IS secured electronic data capture system, and/or REDCap. When it comes time for analysis, the exported data will be coded in that each subject will be identified only by participant number. All access is restricted to CHOP's network.
2. **Security**. OnCore Clinical Trials Management System and REDCap are secure electronic data capture systems with access controls and a data backup plan. The system is password protected. Only study team members

will have access to subject data and case report forms stored in OnCore and REDCap. Access to the system is monitored and logged for review if needed.

3. **Anonymization, de-identification or destruction**. A coded data set will be used for this study. Data will not be destroyed.

Here, we describe data security and management specific to Arcus and PEDSnet.

7.3.1 Arcus

Ultrasound images from all PKIDS sites stored and analyzed in the Arcus platform. In addition, data collected from participants at CHOP will archived in Arcus. Arcus is a CHOP enterprise strategic initiative to develop a next-generation data platform. The mission of Arcus is to integrate each patient and/or research subject's biological, clinical, research and environmental data for the purposes of supporting collaborative research, innovation, and discovery. Arcus features a de-identified clinical data repository, as well as a research data archives and library-based discovery service that preserves, manages, and makes CHOP's research data discoverable alongside the contextual information and tools required for reusable research.

Investigators and assigned project staff can securely store, access, and process project data in Arcus. The Arcus program is staffed by over twenty archivists, librarians, information analysts, cloud computing engineers, programmers, statisticians, and privacy experts. Research data sets are managed under the oversight of the CHOP Institutional Review Board and access is governed by multiple institutional policies. Clinical and research data are linked through an electronic honest broker and made available to researchers with a CHOP-issued credential. Arcus security configuration and controls are based on the HIPAA Security Rule, and are subject to audit by CHOP's independent Internal Audit department which reports directly to the CHOP Board of Trustees.

Arcus ensures the security of its users and data by addressing the following four topics:

1. Authentication
    a. All users are required to use their CHOP Active Directory credentials to access the Arcus environment
    b. All users will be granted a temporary authentication token upon logging into the Arcus platform
    c. The temporary authentication token will expire after one (1) hour
    d. The authentication request information will be logged. This will include, but is not limited to: username, IP address, time, authentication token, expiration time, etc.
        i. All authentication requests will be logged, monitored, and assessed for anomalous behavior which would result in an alert.
2. Authorization

   a. Users are tied to a role-based model which associates users with the least permissive role needed to authorize their requests
   b. Whenever possible, individual user access will be denied in favor of service accounts
   c. All authorizations will be logged, monitored, and assessed for anomalous behavior which would result in an alert.
3. Access
   a. With a successful combination of Authentication and Authorization, a user, service account, or an administrator will possess an access key.
   b. All access is restricted to CHOP's network
   c. All actions on the Arcus platform are tracked
   d. All data access events or API calls will be logged, monitored, and assessed for anomalous behavior which would result in an alert.
4. Auditability
   a. Besides logging, monitoring, and alerting based on anomalous behavior, all data related to Authentication, Authorization, and Access will be saved permanently in a geo-redundant, versioned storage location.
   b. These data are immutable and can be used to track such use cases as: users actions, events in a certain time frame, the events associated with a certain data, a user's actions in a given workspace, etc.
   c. Governance
      i. Arcus provides and maintain a suite of governance documents which will dictate the terms for use and sharing in an effort to secure the data stored within Arcus, while at the same minimizing the obstacles for future use and maximizing its usefulness (e.g. the Arcus Terms of Use clearly provide for the prohibition of release of any Arcus information.)

7.3.2 PEDSnet

The information below describes the data management and security that will govern the data pull for ED visits, hospitalizations, clinic visits, and surgical (re)treatment by querying the PEDSnet Common Data Model. All PKIDS sites that are members of PEDSnet are HIPAA covered entities, and as such maintain detailed patient privacy policies, which are communicated to patients routinely as part of health care operations. Several additional steps have been taken to reduce risk to individuals' privacy, including:

- Use of HIPAA limited datasets for the core data resource and data sharing. These datasets will not contain external individual identifiers such as name or medical record number. As described below under use of PHI, they do include complete dates and low-level aggregate geographic regions.
- Use of cryptographically strong pseudoidentifiers as unique tags for individuals, and of privacy-protecting record linkage methods to allow connection of information about an individual from different data sources without use of clear text identifiers.

- Adoption of PEDSnet Data Privacy Principles in PKIDS. Consistent with ethical research and current best practices, PEDSnet has formally specified these principles to govern data collection, sharing, and research use. The implementation of practices consistent with these policies is guided by the Privacy and Data Security task force.
- Use where feasible of aggregate data or derived data elements (e.g. ages rather than dates) in analytic datasets produced from the data resource.
- Use where feasible of risk-reducing strategies such as low-count cell (5 or fewer) suppression in executing queries against the data resource.
- Limitation of published results to aggregate data and patterns that do not permit reidentification of individuals. PEDSnet Sites also maintain institutional policies and practices for data security, and PEDSnet work at each site follows these institutional practices. In addition to these institutional policies, and the risk minimization techniques listed above, additional data security measures include:
- Use of a central PEDSnet DCC (at CHOP) to mediate query evaluation, execution, and archiving, which allows for consistent assessment of privacy risks.
- Implementation of secure transport mechanisms for transmission of data between the PEDSnet DCC and data contributors.
- Use of secure transport and strong person-specific authentication for access by users to query tools provided by the PEDSnet DCC.
- Requirement for secure storage of data by research users, and prohibition on redisclosure or dissemination of datasets posing high risk of individual reidentification.

7.4 Confidentiality

All data and records generated during this study will be kept confidential in accordance with CHOP policies and HIPAA on subject privacy. The Investigators and other study personnel will not use such data and records for any purpose other than conducting the study. The breach of confidentiality has been made a minimal risk by assigning participants a unique study identification number, de-identifying the data set, storing data on secure, password protected, computers, and completing all data analyses and one study site. Safeguards are described under Data Collection and Management.

7.5 Regulatory and Ethical Considerations

7.5.1 Data and Safety Monitoring Plan

In accordance with the PCORI guidelines, this study requires a Data Safety Monitoring Plan (DSMP). As there are no risks additional to the delivery of clinical care other than data security and privacy, an external Data Safety Monitoring Board is not proposed. For the proposed research, study coordinators will prepare quarterly assessments of participant burden, including reasons for any dropouts, which will be reviewed by Dr. Tasian. PI Tasian will provide oversight for the study

personnel to ensure that confidentiality is maintained and that any adverse events are identified and reported in a timely manner. The IRB will also monitor safety. The study protocol will be carried out in accordance with OHRP and PCORI guidelines and requirements. In the unlikely event of a serious adverse event during the study protocol, it will be reported immediately to Dr. Tasian, the steering committee, and, if necessary, the PEDSnet DCC. It will also be reported to the IRB, PCORI, and to all members of the research team. With the approval of the patient participants and families, the information will be provided to the care providers as directed.

7.5.2 Risk Assessment

The proposed project involves the collection of information from children, adolescents, and young adult patients. The risk of participation is minimal. The proposed study involves surgeries that are delivered as part of routine clinical care, assessment of stone clearance using ultrasound obtained for clinical purposes, and patient-reported outcomes. Potential risks include: breach of confidentiality of personal health information and the risk of patients feeling uncomfortable completing study measures. The study team has taken steps to mitigate these risks. Please see Section 8.2 for processes the ensure confidentiality. The study team has mitigated the risk of patients feeling uncomfortable with study measures by including language in patient consent stating that all participation is voluntary; patients will be allowed to stop participating in the study at any time. Should any further concerns arise throughout the study, the study team will immediately notify the appropriate parties, including the CHOP IRB (the IRB of record). We expect adverse consequences to be rare and unlikely.

7.5.3 Potential Benefits of Study Participation

There is no direct benefit from study participation. However, the results of this study may assist health care providers and pediatric patients with kidney stones, and their caregivers in shared decision-making regarding surgical options for kidney stones. This model may be replicated in different care settings and other diseases and thus generate generalizable knowledge. In addition, this study generates knowledge that will help caregivers in shared decision-making regarding surgical options for kidney stones. This model may be replicated in different care settings and other diseases and thus generate generalizable knowledge. Further, the information gathered from this study will be disseminated widely, including to peer-reviewed journals, lay publications, and presentations at scientific meetings.

7.5.4 Risk-Benefit Assessment

The potential risks associated with study procedures are minimal. Results gained from this comparative effectiveness study will help in the selection of appropriate surgical treatments for pediatric patients with kidney stone disease and inform the development of comparative effectiveness models for other surgical diseases.

7.6 Recruitment Strategy

Individuals who are undergoing elective URS, SWL, or PCNL for the removal of at least one kidney stone are eligible for inclusion. Patient participants may be recruited from urology and nephrology ambulatory clinics, inpatient hospitalization, the ED, and other points of care through which a patient is referred for surgical interventions. Recruitment methods may vary across PKIDS sites. For example, upcoming clinic schedules and lists of hospitalized patients may be scanned to identify potentially eligible participants. EHR alerts and recruitment materials, such as flyers and videos, may also be used to recruit eligible patient participants.

Surgeon participants will be identified as the urologist performing the procedure for the identified patient participants.

7.7 Informed Consent/Assent and HIPAA Authorization

Potentially eligible patient participants may be contacted prior to in-person or telephone recruitment by multiple methods unique to each site. These may include phone, letter, email, social media, or secure EHR messaging. Their providers may be alerted about plans for recruitment, as appropriate. Patients who are not hospitalized may be called before an upcoming clinic visit to inform them about the study. When feasible, with sufficient amount of time to consider study participation, patients who present to the emergency department will be included. During the initial discussion with the eligible patient participant, the site PI or his/her designee will explain the purpose of the study in language understandable to individuals 8 – 21 years of age. Individuals will be able to enroll in the study up to and including the day of surgery.

There will be multiple options for enrollment to meet the needs of patient participants, including in-person and telephone consent. The informed consent process for an in-person consent may occur at ambulatory clinic visits, inpatient hospitalizations, or in the emergency department. A member of the research team will inform the patient and their family about the study and, if they agree to participate, obtain consent at that time. Participants will be dutifully informed of the requirements for study participation. This includes informing the patient participants that they need to complete at-home questionnaires and return to clinic to have an ultrasound. Patient participants who are being recruited by phone will be given information about the study, the risks and benefits associated with the study, and contact information for the study team. Those who agree to participate will provide verbal consent for their participation. Signed and verbal consents will be maintained by the study coordinator and will be reviewed by the site PI. Assent (or the inability to assent) will be documented on the consent form.

Patient participants who turn 18 years old after initial consent but prior to completing the study procedures will be asked to sign a new consent form to continue in the study. We will use a combined consent-HIPAA authorization document.

7.7.1 Waiver of Documentation of Consent

A waiver of documentation of consent is being requested for recruiting patient participants via telephone. This will allow the coordinator to recruit eligible patient participants via medical record review. This research involves no more than minimal risk to participants and this waiver will not adversely affect the rights of the participants.

A wavier of documentation of consent is also being requested for surgeon participants. A simple consent form will be used on REDCap to obtain consent by clicking the “I Agree” button and the end of the form.

7.7.2 Waiver of Assent

A waiver of assent is being requested for patient participants contacted over the phone because it is anticipated that a child may not be present at the time the parent is providing consent. This will allow eligible patient participants to be enrolled via telephone. This research involves no more than minimal risk to participants and this alteration will not adversely affect the rights of the patient participants.

7.7.3 Alteration of HIPAA Authorization to Obtain Verbal Authorization

An alteration of HIPAA Authorization is being requested for recruiting patient participants via telephone. Since members of the research team may not meet the patient participant they only contact over the phone, it would be impracticable to conduct the research without the alteration to obtain verbal authorization. Participant study identification numbers will be used on all study materials as identifiers. This will remove all patient participant medical identifiers from study materials. This research involves no more than minimal risk to participants and this alteration will not adversely affect the rights of the patient participants.

7.7.4 Waiver of HIPAA Authorization

A waiver of HIPPA Authorization is being requested for medical record review to assess eligibility. Since members of the research team are using the medical records to identify eligible individuals, it would be impracticable to conduct the research without the waiver.

7.8 Payment to Subjects/Families

7.8.1 Payments to subject for time, effort and inconvenience (i.e. compensation)

To compensate for the time, effort, and inconvenience of participating in the study, the patient participant will receive a financial incentive. The specific amount distributed will be determined by each site, but will not exceed $100.

Surgeon participants will not be compensated.

8 PUBLICATION

The foundation of this project is a network of pediatric urologists who are committed to increasing the evidence base for the surgical management of pediatric kidney stones and applying this knowledge to improve patients' lives. Translating this knowledge to other settings outside the PKIDs network will require building a community of practice. A community of practice is a network of professionals with common beliefs, values, and experiences who work towards a mutual goal by leveraging knowledge acquisition/exchange, translation, and application.[77] In this community, transformational learning occurs within a professional context, situated in how its members practice. Practically, a community of practice functions with experts situated at the center and those recently joining on the periphery. As new members participate in knowledge sharing through socialization they increasingly move towards the center as experts.

PKIDS investigators will serve as the experts situated in the center of a community of practice. They will be armed with the best practices in the surgical management of pediatric kidney stones, and have experience modifying their local practices, and the practices of the 22 PKIDS sites. When interacting with peers from other institutions, they will invite them to join the community, and as these new members participate, knowledge will be exchanged and practices will be improved. To operationalize this community of practice and generate participation outside of the PKIDs sites, we will leverage the existing relationships of the investigators. We will open the community to members of the Societies for Pediatric Urology, American Urological Association, and the Endourological Society. We will further our reach via peer-reviewed publications, study website, social media, and presentations to primary care practices.

The infrastructure to support this community of practice will be an online educational hub used to store knowledge, including peer-reviewed articles, visual abstracts summarizing key study findings, educational pamphlets and videos, and cultural change management tools. The Patient and Family Partners and Clinician Council will co-produce this material. Specifically, we will develop parent-oriented pamphlets and videos explaining caregivers' experiences with surgery on their child and child-oriented guides and videos explaining children's experiences with their own surgery. We will develop a model to guide other institutions through the steps necessary to adopt best practices in the surgical management of pediatric kidney stones. The most important aspect of the hub is a virtual portal for clinicians to interact and exchange educational material through open discussion forums and monthly video chats, each moderated by a PKIDS expert, including Patient and Family Partners, to provide feedback and reinforcement. We will further strengthen engagement by extending the community to in-person gatherings at meetings that are widely attended by pediatric urologists. In particular, the study capstone will be a national symposium on the surgical management of pediatric kidney stones. We anticipate that many of members from our community of practice will attend. We will include other stakeholders including patients, healthcare administrators, payers, patient advocates, and industry. The symposium will ensure that all perspectives critical to successfully implementing results into all phases of clinical care are considered.

This symposium will set the stage for future projects that develop surgical learning health systems at PKIDS sites.

9 REFERENCES

1. Hardin AP, Hackell JM, Committee On P, Ambulatory M. Age Limit of Pediatrics. Pediatrics 2017;140.

2. Scales CD, Jr., Smith AC, Hanley JM, Saigal CS, Urologic Diseases in America P. Prevalence of kidney stones in the United States. Eur Urol 2012;62:160-5.

3. Pearle M, Calhoun E, Curhan G. Urologic Diseases in America Project: Urolithiasis. The Journal of urology 2005;173:848-57.

4. Khan SR, Pearle MS, Robertson WG, et al. Kidney stones. Nat Rev Dis Primers 2016;2:16008.

5. Ellison JS, Williams M, Keeley FX, Jr. Patient-Reported Outcomes in Nephrolithiasis: Can We Do Better? J Endourol 2017.

6. Penniston KL, Antonelli JA, Viprakasit DP, et al. Validation and Reliability of the Wisconsin Stone Quality of Life Questionnaire. J Urol 2017;197:1280-8.

7. Penniston KL, Nakada SY. Health related quality of life differs between male and female stone formers. J Urol 2007;178:2435-40; discussion 40.

8. Letavernier E, Traxer O, Daudon M, et al. Determinants of osteopenia in male renal-stone-disease patients with idiopathic hypercalciuria. Clinical journal of the American Society of Nephrology : CJASN 2011;6:1149-54.

9. Asplin JR, Donahue S, Kinder J, Coe FL. Urine calcium excretion predicts bone loss in idiopathic hypercalciuria. Kidney Int 2006;70:1463-7.

10. Denburg MR, Jemielita TO, Tasian GE, et al. Assessing the risk of incident hypertension and chronic kidney disease after exposure to shock wave lithotripsy and ureteroscopy. Kidney Int 2016;89:185-92.

11. Ristau BT, Dudley AG, Casella DP, et al. Tracking of radiation exposure in pediatric stone patients: The time is now. J Pediatr Urol 2015;11:339 e1-5.

12. Stamatelou KK, Francis ME, Jones CA, Nyberg LM, Curhan GC. Time trends in reported prevalence of kidney stones in the United States: 1976-1994. Kidney Int 2003;63:1817-23.

13. Robertson WG. Stone formation in the Middle Eastern Gulf States: A review. ARAB JOURNAL OF UROLOGY 2012:1-8.

14. Routh JC, Graham DA, Nelson CP. Epidemiological trends in pediatric urolithiasis at United States freestanding pediatric hospitals. J Urol 2010;184:1100-4.

15. Sas DJ, Hulsey TC, Shatat IF, Orak JK. Increasing incidence of kidney stones in children evaluated in the emergency department. J Pediatr 2010;157:132-7.

16. Tasian GE, Ross ME, Song L, et al. Annual Incidence of Nephrolithiasis among Children and Adults in South Carolina from 1997 to 2012. Clinical journal of the American Society of Nephrology : CJASN 2016;11:488-96.

17. Dabelea D, Mayer-Davis EJ, Saydah S, et al. Prevalence of type 1 and type 2 diabetes among children and adolescents from 2001 to 2009. JAMA 2014;311:1778-86.

18. Bush NC, Xu L, Brown BJ, et al. Hospitalizations for pediatric stone disease in United States, 2002-2007. J Urol 2010;183:1151-6.

19. Wang HH, Wiener JS, Lipkin ME, Scales CD, Jr., Ross SS, Routh JC. Estimating the nationwide, hospital based economic impact of pediatric urolithiasis. J Urol 2015;193:1855-9.

20. Kusumi K, Becknell B, Schwaderer A. Trends in pediatric urolithiasis: patient characteristics, associated diagnoses, and financial burden. Pediatr Nephrol 2015;30:805-10.

21. Lao M, Kogan BA, White MD, Feustel PJ. High recurrence rate at 5-year followup in children after upper urinary tract stone surgery. J Urol 2014;191:440-4.

22. Tasian GE, Kabarriti AE, Kalmus A, Furth SL. Kidney Stone Recurrence among Children and Adolescents. J Urol 2017;197:246-52.

23. Johnson CM, Wilson DM, O'Fallon WM, Malek RS, Kurland LT. Renal stone epidemiology: a 25-year study in Rochester, Minnesota. Kidney Int 1979;16:624-31.

24. Ljunghall S, Danielson BG. A prospective study of renal stone recurrences. Br J Urol 1984;56:122-4.

25. Dangle P, Ayyash Ot, Shaikh H, 3rd, et al. Predicting Spontaneous Stone Passage in Prepubertal Children: A Single Institution Cohort. J Endourol 2016;30:945-9.

26. Tasian GE, Cost NG, Granberg CF, et al. Tamsulosin and the spontaneous passage of ureteral stones in children: A multi-institutional cohort study. J Urol 2014.

27. Routh JC, Graham DA, Nelson CP. Trends in imaging and surgical management of pediatric urolithiasis at American pediatric hospitals. J Urol 2010;184:1816-22.

28. Iremashvili V, Li S, Penniston KL, Best SL, Hedican SP, Nakada SY. Role of Residual Fragments on the Risk of Repeat Surgery after Flexible Ureteroscopy and Laser Lithotripsy: Single Center Study. J Urol 2019;201:358-63.

29. Zanetti G, Seveso M, Montanari E, et al. Renal stone fragments following shock wave lithotripsy. J Urol 1997;158:352-5.

30. Osman MM, Alfano Y, Kamp S, et al. 5-year-follow-up of patients with clinically insignificant residual fragments after extracorporeal shockwave lithotripsy. Eur Urol 2005;47:860-4.

31. Streem SB, Yost A, Mascha E. Clinical implications of clinically insignificant store fragments after extracorporeal shock wave lithotripsy. J Urol 1996;155:1186-90.

32. Raman JD, Bagrodia A, Gupta A, et al. Natural history of residual fragments following percutaneous nephrostolithotomy. J Urol 2009;181:1163-8.

33. Assimos D, Krambeck A, Miller NL, et al. Surgical Management of Stones: American Urological Association/Endourological Society Guideline, PART I. J Urol 2016;196:1153-60.

34. Assimos D, Krambeck A, Miller NL, et al. Surgical Management of Stones: American Urological Association/Endourological Society Guideline, PART II. J Urol 2016;196:1161-9.

35. Pearle MS, Lingeman JE, Leveillee R, et al. Prospective, randomized trial comparing shock wave lithotripsy and ureteroscopy for lower pole caliceal calculi 1 cm or less. JURO 2005;173:2005-9.

36. Strippoli GF, Craig JC, Schena FP. The number, quality, and coverage of randomized controlled trials in nephrology. J Am Soc Nephrol 2004;15:411-9.

37. National Urology Research Agenda. American Urological Association, 2018. (Accessed January 16, 2018, at http://www.auanet.org/research/resources-and-initiatives/national-urology-research-agenda-(nura).)

38. Forrest CB, Bevans KB, Tucker C, et al. Commentary: the patient-reported outcome measurement information system (PROMIS(R)) for children and youth: application to pediatric psychology. Journal of pediatric psychology 2012;37:614-21.

39. Revicki DA, Cella DF. Health status assessment for the twenty-first century: item response theory, item banking and computer adaptive testing. Qual Life Res 1997;6:595-600.

40. Hung M, Stuart AR, Higgins TF, Saltzman CL, Kubiak EN. Computerized Adaptive Testing Using the PROMIS Physical Function Item Bank Reduces Test Burden With Less Ceiling Effects Compared With the Short Musculoskeletal Function Assessment in Orthopaedic Trauma Patients. J Orthop Trauma 2014;28:439-43.

41. Joshi HB, Newns N, Stainthorpe A, MacDonagh RP, Keeley FX, Jr., Timoney AG. Ureteral stent symptom questionnaire: development and validation of a multidimensional quality of life measure. J Urol 2003;169:1060-4.

42. Joshi HB, Okeke A, Newns N, Keeley FX, Jr., Timoney AG. Characterization of urinary symptoms in patients with ureteral stents. Urology 2002;59:511-6.

43. PROMIS Methodology Standards. (Accessed February 2, 2018, at http://www.healthmeasures.net/explore-measurement-systems/promis/measure-development-research. .)

44. Willis G. Cognitive interviewing: a tool for improving questionnaire design: Sage Publications; 2004.

45. Fortune-Greeley A, Flynn K, Jeffery D, et al. Using Cognitive Interviews to Evaluate Items for Measuring Sexual Functioning Across Cancer Populations: Improvements and Remaining Challenges2009.

46. Irwin DE, Varni JW, Yeatts K, DeWalt DA. Cognitive interviewing methodology in the development of a pediatric item bank: a patient reported outcomes measurement information system (PROMIS) study. Health Qual Life Outcomes 2009;7:3.

47. Masch WR, Cohan RH, Ellis JH, Dillman JR, Rubin JM, Davenport MS. Clinical Effectiveness of Prospectively Reported Sonographic Twinkling Artifact for the Diagnosis of Renal Calculus in Patients Without Known Urolithiasis. AJR Am J Roentgenol 2016;206:326-31.

48. Gliga ML, Chirila CN, Podeanu DM, et al. Twinkle, twinkle little stone: an artifact improves the ultrasound performance! Med Ultrason 2017;19:272-5.

49. Dunmire B, Lee FC, Hsi RS, et al. Tools to improve the accuracy of kidney stone sizing with ultrasound. J Endourol 2015;29:147-52.

50. Dunmire B, Harper JD, Cunitz BW, et al. Use of the Acoustic Shadow Width to Determine Kidney Stone Size with Ultrasound. J Urol 2016;195:171-7.

51. Passerotti C, Chow JS, Silva A, et al. Ultrasound versus computerized tomography for evaluating urolithiasis. J Urol 2009;182:1829-34.

52. Harris PA, Taylor R, Thielke R, Payne J, Gonzalez N, Conde JG. Research electronic data capture (REDCap)--a metadata-driven methodology and workflow process

for providing translational research informatics support. Journal of biomedical informatics 2009;42:377-81.

53. Parzen M, Ghosh S, Lipsitz S, et al. A generalized linear mixed model for longitudinal binary data with a marginal logit link function. Ann Appl Stat 2011;5:449-67.

54. Robins JM, Hern, xe, et al. Marginal Structural Models and Causal Inference in Epidemiology. Epidemiology 2000;11:550-60.

55. Kurth T, Walker AM, Glynn RJ, et al. Results of multivariable logistic regression, propensity matching, propensity adjustment, and propensity-based weighting under conditions of nonuniform effect. Am J Epidemiol 2006;163:262-70.

56. Varadhan R, Segal JB, Boyd CM, Wu AW, Weiss CO. A framework for the analysis of heterogeneity of treatment effect in patient-centered outcomes research. J Clin Epidemiol 2013;66:818-25.

57. Bock RD, Mislevy RJ. Adaptive EAP Estimation of Ability in a Microcomputer Environment. 1982;6:431-44.

58. Varni JW, Stucky BD, Thissen D, et al. PROMIS Pediatric Pain Interference Scale: an item response theory analysis of the pediatric pain item bank. J Pain 2010;11:1109-19.

59. Cella D, Riley W, Stone A, et al. The Patient-Reported Outcomes Measurement Information System (PROMIS) developed and tested its first wave of adult self-reported health outcome item banks: 2005-2008. J Clin Epidemiol 2010;63:1179-94.

60. Wyrwich KW, Tierney WM, Wolinsky FD. Further evidence supporting an SEM-based criterion for identifying meaningful intra-individual changes in health-related quality of life. J Clin Epidemiol 1999;52:861-73.

61. Wyrwich KW, Nienaber NA, Tierney WM, Wolinsky FD. Linking clinical relevance and statistical significance in evaluating intra-individual changes in health-related quality of life. Medical care 1999;37:469-78.

62. Li F, Zaslavsky AM, Landrum MB. Propensity score weighting with multilevel data. Stat Med 2013;32:3373-87.

63. Imbens GW. The Role of the Propensity Score in Estimating Dose-Response Functions. National Bureau of Economic Research Technical Working Paper Series 1999;No. 237.

64. van Dyk DA. Causal Inference With General Treatment Regimes AU - Imai, Kosuke. J Am Stat Assoc 2004;99:854-66.

65. McCaffrey DF, Griffin BA, Almirall D, Slaughter ME, Ramchand R, Burgette LF. A tutorial on propensity score estimation for multiple treatments using generalized boosted models. 2013;32:3388-414.

66. Hernán MA, Robins JM. Estimating causal effects from epidemiological data. 2006;60:578-86.

67. VanderWeele TJ, Hernan MA. Causal Inference Under Multiple Versions of Treatment. J Causal Inference 2013;1:1-20.

68. Rubin DB. On principles for modeling propensity scores in medical research. Pharmacoepidemiol Drug Saf 2004;13:855-7.

69. Austin PC. Balance diagnostics for comparing the distribution of baseline covariates between treatment groups in propensity-score matched samples. Stat Med 2009;28:3083-107.

70. Austin PC. An Introduction to Propensity Score Methods for Reducing the Effects of Confounding in Observational Studies. Multivariate Behav Res 2011;46:399-424.

71. Nannicini T. Simulation-based sensitivity analysis for matching estimators. Stata Journal 2007;7:334-50.

72. Li L, Shen C, Wu AC, Li X. Propensity score-based sensitivity analysis method for uncontrolled confounding. Am J Epidemiol 2011;174:345-53.

73. Miller RG. Simultaneous Statistical Inference: Springer; 1966.

74. Freton L, Peyronnet B, Arnaud A, et al. Extracorporeal Shockwave Lithotripsy Versus Flexible Ureteroscopy for the Management of Upper Tract Urinary Stones in Children. J Endourol 2017;31:1-6.

75. Dogan HS, Altan M, Citamak B, Bozaci AC, Karabulut E, Tekgul S. A new nomogram for prediction of outcome of pediatric shock-wave lithotripsy. J Pediatr Urol 2015;11:84 e1-6.

76. Bilen CY, Koçak B, Kitirci G, Ozkaya O, Sarikaya S. Percutaneous nephrolithotomy in children: lessons learned in 5 years at a single institution. JURO 2007;177:1867-71.

77. Teaching and Learning in Medical Education: How Theory can Inform Practice. Understanding Medical Education.

APPENDIX

Append relevant information.

Protocol Amendments Since Initial Institutional Review Board (IRB) Approval

Version Number	Approved Modifications	Date of IRB Approval
2.0	• Adding of post-op questionnaires time point (9 weeks post-op)<br>• Decrease to enrollment numbers at non-CHOP sites<br>• New recruitment flyers and brochures<br>• Allows sharing of subject contact information for related research<br>• Addition of DSMP<br>• Clarification of timing of subjects' post-operative medication logging<br>• Adding an additional round of PROs for subjects who require staged procedure together with and updated compensation plan<br>• Clarification of timing of procedures<br>• Minor edits to improve consent form readability	28 Feb 2020<br>*(Prior to first participant enrollment)*
2.1	• Clarification of the enrollment breakdown for surgeons vs patients<br>• Clarification that surgeons at all sites are enrolled by CHOP<br>• Providing a Phone Short Form Consent plan	09 Jun 2020
3.0	• Adding of option for REDCap e-consent for patient participants<br>• Increase to enrollment goals for surgeons from 120 to 200<br>• Clarification of compensation and payment method across all sites<br>• Adding of additional COVID-19 question<br>• Adding of Spanish patient-facing questionnaires<br>• Update of recruitment materials<br>• Adding Twilio, Inc to list of entities that may access PHI<br>• Minor updates and clarifications throughout study materials	04 Nov 2020
4.0	• Updated and new recruitment materials (for both CHOP and relying institutions)<br>• Minor edits to the protocol pertaining to documentation for consenting Limited English Proficiency subjects virtually/over the phone	18 Jan 2022
4.1	• Revision of the inclusion/exclusion criteria within the protocol	19 Apr 2022

5.0	• Addition of semi-structured interview component for surgeons regarding result dissemination<br>• Protocol and surgeon consent forms were revised accordingly	16 Jun 2022
5.1	• Addition of Microsoft Word as the method of transcription for Surgeon Interviews	08 Jul 2022

Ureteroscopy versus Shockwave Lithotripsy to Remove Kidney Stones in Children and Adolescents:

Trial Protocol/Statistical Analysis Plan

Statistical Analysis Plan

The study aims to compare the effectiveness of Ureteroscopy (URS) and shockwave lithotripsy (SWL) as surgical treatments for kidney stone removal and patient experience in pediatric patients. The primary outcome is stone clearance, defined as the absence of any stone with a longest dimension greater than 4mm in an ultrasound (US) scan performed within 4-8 weeks post-surgery. If multiple images are available, the one closest to 6 weeks will be used. Secondary outcomes include patient experiences measured by patient-reported outcomes (PROs) using 5 PROMIS domains, DVSS, and QUIKSS at baseline (before surgery), and at 1 week (7 days +/- 3 days), 3 weeks (21 days +/- 7 days), 6 weeks (45 days +/- 7 days), and 3 months (90 days +/- 7 days) after surgery. We used propensity scores and inverse-probability-weighting adjustment to reduce treatment-selection bias when comparing outcomes.

Study unit and assessment of treatment

The primary outcome, stone clearance, is assessed at the kidney level, with the treatment defined as the first surgery performed on each kidney. For the secondary outcomes, the PROs, the study unit is the patient, with the treatment defined as the patient's first surgery.

Propensity score model

A logistic regression model was used to estimate the propensity scores for receiving treatment. The construction of the propensity score model involved selecting confounders from a large pool of candidates related to treatment selection. These candidates included baseline characteristics (37 variables), patient conditions from the pre-op form (12 variables), stone characteristics from the pre-op imaging form (13 variables), surgery-related characteristics from the surgery form (27 variables), and institutional and surgeon demographics (62 variables).
The pool of candidate confounders was pre-screened based on the clinical importance scores provided by domain experts in the Data and Outcomes Committee (DOC), which included the study PI, site PIs, and statisticians. Variables were also assessed for their correlation with other variables. Within each category, variables were ranked based on their clinical importance and correlation scores. Correlation scores were defined by the number of correlated variables with a correlation coefficient greater than 0.3, considered moderate correlation (Cramér's V of 0.3). Variables that were less important or highly correlated with higher-ranked variables were removed. Specifically, we started from the top of the list and removed variables if they had a correlation coefficient exceeding 0.4 with higher-ranked variables, (a Cramér's V of 0.5 is considered strong correlation), while retaining the variable with higher clinical importance. This procedure, conducted without analyzing the outcome data, yielded a list of pre-screened confounders. The pre-screened
confounders were further examined using univariate analysis to evaluate their associations with the

surgical treatment. Variables with a p-value less than 0.2 were selected and reviewed by the DOC. The final list of confounders was determined by the DOC, combining domain knowledge with the estimated strength of association. The list is shown in Table 1.

We verified the performance of the propensity score model by comparing the distribution of confounders between treatment groups both before and after inverse probability weighting (IPW). Confounders with a standardized difference in median (SDM) greater than 0.25 after weighting were included in the outcome models for further adjustment.

Table 1. The final list of confounders used in the propensity score model.

Domains	Variables
Baseline Characteristics	experience with prior surgery, problems with blood cells, muscle problems
Pre-op variables	age, prior GU reconstruction, monogenic stone disease
Surgery variables	structural renal abnormality, primary clinical indication for surgery, surgery within 24 hours of symptom evaluation, restrictions to OR access
Imaging variables	imaging type, number of stones, stone location, stone size
Institutional and surgeon Characteristics	number of kidney stone patients evaluated in outpatient clinic during 2018-2019, the importance of patient age in surgeon’s surgical decision-making, the importance of family’s preference in surgeon’s surgical decision-making, the importance of stone size in surgeon’s surgical decision-making, the importance of availability of equipment in surgeon’s surgical decision-making, surgeon’s preference for URS versus PCNL for a 1.5 cm kidney stone, surgeon’s preference for SWL versus PCNL for a 2 cm kidney stone

Acronyms: Pre-op; Preoperative, GU; Genitourinary, OR; operating room, URS; Ureteroscopy, PCNL; Percutaneous nephrolithotomy, SWL; Shockwave Lithotripsy

Outcome model

Both the primary and secondary outcomes were compared using the IPW approach. The outcomes were compared using regression models with IPW and adjusted for unbalanced confounders after weighting. The analysis of PROs was conducted at 1-week, 3-weeks, 6-weeks, and 3-months after the index surgery adjusting for baseline scores.

Analysis of heterogeneity of treatment (HTE)

HTE was analyzed by both stratified analyses and regression models with interaction terms. HTE of stone clearance was evaluated by stone size (<7mm, 7-10mm, 11-15mm, >15mm) and location (lower pole, non-lower pole, ureter). HTE of PROs was evaluated by patient age group (8-11, 12-15, 16-18, 19-21) and sex. Categories were defined *a priori* by the DOC.

Missing data

We used multiple imputation (MI) to handle missing data. Variables used to impute missing data included all baseline covariates, treatment variable, and observed outcomes that were used in the analysis. Twenty imputed datasets were created and analyzed separately, and the results were combined using Rubin's rules to produce valid statistical inferences. MI was used for the analyses of stone clearance, PROs at week 1, and HTE of both primary and secondary analyses.

Sensitivity analysis

We conducted a complete data analysis assuming the missingness was completely random. In addition, to assess the impact of potential missing not at random mechanisms on the study results for stone clearance, we performed a sensitivity analysis using pattern-mixture models. We assumed that patients with missing outcomes had different stone clearance rates compared to those without missing data. The scenarios included:

1. Patients with missing outcomes had a 100% stone clearance rate, regardless of the surgery received.
2. Patients with missing outcomes had a 0% stone clearance rate, regardless of the surgery received.
3. Patients with missing outcomes had stone clearance rates ranging from a -25% to 25% change from observed rates within each treatment group.
4. Patients with missing outcomes had stone clearance rates changing oppositely between the two treatments, ranging from 0% to 100%.

We also conducted a sensitivity analysis for the assessment window of the stone clearance outcome using ultrasounds obtained between 0 to 16 weeks, selecting those closest to the 6-week post-surgery time-point. Additionally, we performed a central review of 10% randomly selected post-op images. We calculated the sensitivity and specificity of the misclassification of the primary outcome and updated the weights after incorporating them with IPW to correct bias due to misclassification.
