# Supplementary material for: Ureteroscopy vs Shockwave Lithotripsy to Remove Kidney Stones in Children and Adolescents: A Nonrandomized Clinical Trial
Source: JAMA Netw Open. 2025 Aug 7;8(8):e2525789. doi: 10.1001/jamanetworkopen.2025.25789 (PMC12332628; doi:10.1001/jamanetworkopen.2025.25789)
Supplement: Supplement 3. — Data Sharing Statement [file jamanetwopen-e2525789-s003.pdf]

Data Sharing Statement

Tasian. Ureteroscopy vs Shockwave Lithotripsy to Remove Kidney Stones in Children and Adolescents. *JAMA Netw Open*. Published August 07, 2025.
doi:10.1001/jamanetworkopen.2025.25789

Data
Additional Information: ClinicalTrials.gov Identifier: NCT04285658
Data available: Yes
Data types: Deidentified participant data, Data dictionary
How to access data: Investigators may submit proposals for secondary analysis of PKIDS trial data, which will be reviewed by the PKIDS Publications and Study Initiation Committee per the Publications Guidelines. Proposals for data access will be considered from investigators in and outside the PKIDS network. All data requests can be sent to Dr. Tasian at tasiang@chop.edu
When available: beginning date: 10-12-2025

Supporting Documents
Document types: Statistical/analytic code, Informed consent form
How to access documents: Please request statistical analysis code and ICFs from Dr. Tasian at tasiang@chop.edu
When available: With publication

Additional Information
Who can access the data: Researchers whose proposed use of the data has been approved
Types of analyses: Analyses that align with the mission of the Pediatric KIDney Stone (PKIDS) Care Improvement Network, which is to improve the health of children with kidney stone disease.
Mechanisms of data availability: After approval of a proposal and the necessary data use agreements.
